# Reliability and Validity of Risk Assessment Tools for Violent Extremism: A Systematic Review

**DOI:** 10.1002/cl2.70080

**Published:** 2025-12-07

**Authors:** Sébastien Brouillette‐Alarie, Ghayda Hassan, Wynnpaul Varela, Emmanuel Danis, Sarah Ousman, Pablo Madriaza, Inga Lisa Pauls, Deniz Kilinc, David Pickup, Robert Pelzer, Eugene Borokhovski, the CPN‐PREV team

**Affiliations:** ^1^ École de criminologie Université de Montréal Montreal Quebec Canada; ^2^ Department of Psychology Université du Québec à Montréal Montreal Quebec Canada; ^3^ Department of Psychoeducation and Social Work Université du Québec à Trois‐Rivières Trois‐Rivières Quebec Canada; ^4^ Department of Psychology Philipps‐Universität Marburg Marburg Germany; ^5^ Center for the Study of Learning and Performance Concordia University Montreal Quebec Canada; ^6^ Zentrum Technik Und Gesellschaft Technische Universität Berlin Berlin Germany

**Keywords:** ERG22+, MLG‐V2, reliability, risk assessment, systematic review, TRAP‐18, validity, VERA, violent extremism, violent radicalization

## Abstract

Assessment of the risk of engaging in a violent radicalization/extremism trajectory has evolved quickly in the last 10 years. Guided by what has been achieved in psychology and criminology, scholars from the field of preventing violent extremism (PVE) have tried to import key lessons from violence risk assessment and management, while bearing in mind the idiosyncrasies of their particular field. However, risk tools that have been developed in the PVE space are relatively recent, and questions remain as to their level of psychometric validation. Namely, do these tools consistently and accurately assess risk of violent extremist acting out? To answer this question, we systematically reviewed evidence on the reliability and validity of violent extremism risk tools. The main objective of this review was to gather, critically appraise, and synthesize evidence regarding the appropriateness and utility of such tools, as validated with specific populations and contexts. Searches covered studies published up to December 31, 2021. They were performed in English and German across 17 databases, 45 repositories, Google, other literature reviews on violent extremism risk assessment, and references of included studies. Studies in all languages were eligible for inclusion in the review. We included studies with primary data resulting from the quantitative examination of the reliability and validity of tools used to assess the risk of violent extremism. Only tools usable by practitioners and intended to assess an individual's risk were eligible. We did not impose any restrictions on study design, type, method, or population. We followed standard methodological procedures outlined by the Campbell Collaboration for data extraction and analysis. Risk of bias was assessed using a modified version of the COSMIN checklist, and data were synthesized through meta‐analysis when possible. Otherwise, narrative synthesis was used to aggregate the results. Among the 10,859 records found, 19 manuscripts comprising 20 eligible studies were included in the review. These studies focused on the Terrorist Radicalization Assessment Protocol (TRAP‐18), the Extremism Risk Guidance Factors (ERG22+), the Multi‐Level Guidelines (MLG‐V2), the Identifying Vulnerable People guidance (IVP guidance), and the Violent Extremism Risk Assessment (VERA)—all structured professional judgment tools—as well as *Der Screener—Islamismus*, an actuarial scale. Studies mostly involved adult male participants susceptible to violent extremism (*N* = 1106; *M* = 58.21; SD = 55.14). The types of extremist ideologies endorsed by participants varied, and the same was true for ethnicity and country/continent of provenance. Encouraging results were found concerning the inter‐rater agreement of scales in research contexts (kappas between 0.76 and 0.93), but one of the two studies that examined it in a field setting obtained disappointing results (kappas ranging between of 0.47 and 0.80). Content validity studies indicated that PVE risk tools adequately cover the risk factors and offending processes of individuals who go on to commit extremist violence. Construct validity analyses were few and far between, with results indicating that empirical divisions of scales did not match their conceptual divisions. The internal consistency of subscales was lackluster (Cronbach's alphas between 0.19 and 0.85), whereas full scales demonstrated acceptable internal consistency when assessed (0.80 for the ERG22+ and 0.64 for the IVP guidance). Only one study examined convergent validity, and it revealed a lack of convergence, primarily due to particularities of the scale under study (the MLG‐V2). Discriminant validity analyses were exploratory in nature, but suggested that PVE risk tools might not be ideology‐specific and may apply to both group and lone actors. Finally, although the TRAP‐18 showed a relatively strong postdictive effect size (pooled *r* = 0.62 [0.35–0.77], *p* = 0.000), the results were highly heterogeneous (*I* ^2^ = 86%), and all studies used retrospective designs, meaning the outcome was already known at the time of assessment. As such, no included study evaluated true predictive validity (i.e., the ability to forecast future violent extremist outcomes based on prospective risk assessment). This represents a significant evidence gap. Threats to validity were substantial: (a) Many studies were case studies or had very small samples, (b) nearly all samples were constituted through the triangulation of publicly available data, and (c) convenience outcome measures were often used. Although having imperfect data is better than having no data, the current state of empirical validation precludes the recommendation of one tool over another for specific populations and contexts, and calls for higher‐quality validation studies for PVE risk assessment tools. Nevertheless, these tools constitute useful checklists of relevant risk and protective factors that could be taken into account by evaluators who wish to assess the risk of violent extremism and identify intervention targets.

## 1 Plain Language Summary

Risk assessment tools for violent extremism may be useful in some contexts; however, there is a need for higher quality evaluation studies.

### 1.1 The Review in Brief

This systematic review looks at the validation of tools used to assess the risk of violent extremism. It finds that none of these tools currently meet the standards expected in the field of correctional psychology.

### 1.2 What Is This Review About?

Risk assessment helps people in the justice system make decisions about supervision, early release, and who should get certain services. These tools have been used in prisons and probation systems around the world since the 1980s. But in the area of preventing violent extremism (PVE), the tools are newer, and it's unclear if they are accurate and consistent. This review brings together studies that tested whether these tools work as intended. We looked at how well they measure what they claim to measure (validity) and how consistently they give the same results (reliability).

### 1.3 What Is the Aim of This Review?

This Campbell systematic review examines the reliability and validity of risk tools for the assessment of violent extremism. It is based on 20 studies that tested these tools and looked at how strong the research evidence is.

### 1.4 What Are the Main Findings of This Review?

The 20 studies looked at six tools: the Terrorist Radicalization Assessment Protocol (TRAP‐18), the Extremism Risk Guidance (ERG22+), the Multi‐Level Guidelines (MLG‐V2), the Identifying Vulnerable People guidance (IVP guidance), the Violent Extremism Risk Assessment (VERA), and *Der Screener—Islamismus*. Studies mostly comprised adult men who adhered to various extremist ideologies (far right, Islamist, nationalist, incel, etc.) and came from multiple countries and continents.

Many studies had major limitations. Some had very small samples or used publicly available data (like news articles, biographies, or databases). Many also used convenience outcome measures, like whether an attack was stopped or not. Importantly, none of the studies used a “prospective” design—meaning that none tested whether the tools could actually predict future violence based on an assessment done before anything happened. Instead, all the studies looked backward in time, after outcomes were already known. These are called postdictive studies.

There were, however, some positive results. In research settings, different experts often gave similar scores when using the same tool (inter‐rater agreement), though this was not always true in real‐world settings. Studies on content validity found that most tools include risk factors linked to extremist violence. Discriminant validity results suggested that these tools might work for both individuals and groups, and for different types of ideologies. But while some tools showed strong results in postdictive validity studies, there was a lot of variation across studies, and none truly tested predictive validity.

### 1.5 What Do the Findings of This Review Mean?

Right now, we cannot say that one tool is better than another. These tools should not be used as the only source of information to make important security decisions. However, they can still help professionals think about relevant risk and protective factors and plan support or interventions. More high‐quality research is needed to test how well these tools work in real‐world situations.

### 1.6 How Up‐to‐Date Is This Review?

The review includes studies published up to December 31, 2021.

## 2 Introduction

### 2.1 Background

#### 2.1.1 The Problem, Condition, or Issue

Assessment of the risk of engaging in or desisting from a violent radicalization trajectory has evolved quickly in the last 10 years. “Standing on the shoulders of giants” (Logan and Lloyd 2019) of what has been achieved in psychology and criminology over the last 50 years, scholars from the field of PVE have tried to import key lessons from violence risk assessment and management while taking into account the idiosyncrasies of their particular field (e.g., Borum 2015).

The advantages of having reliable and valid tools to anticipate and mitigate the risk of violent extremism cannot be underestimated. Law enforcement and intelligence professionals must assess persons of concern before they become involved in planning or executing attacks (threat assessment), and the criminal justice system must determine when and under what circumstances inmates may be released (risk assessment; Borum 2015). Mental health and psychosocial professionals are now often called to perform violent extremism risk assessments in the same way they would for suicidal or homicidal risks among their clients (Borum 2015; Logan and Lloyd 2019). While PVE risk tools are often positioned as useful for both disruption and rehabilitation purposes (e.g., Meloy 2017), in the broader field of criminology, instruments such as the Level of Service/Case Management Inventory (LS/CMI; Andrews et al. 2004) and the Static‐99R (Helmus et al. 2022) are usually reserved for tertiary prevention spaces—that is, to assess release viability and conditions, and to guide prevention and rehabilitative interventions (Bonta and Andrews 2024; Mullen 2000).

In the field of correctional psychology, rehabilitative approaches emphasize the relevance of risk assessments for correctional intervention and service delivery or, in other words, risk reduction via treatment, capacity building, and social reinsertion (Brouillette‐Alarie and Lussier 2018). Initially dominated by a “nothing works” mindset (Martinson 1974), the fields of psychology and criminology funded research on the determinants of effective correctional programming, leading to the identification of the risk‐need‐responsivity principles^1^ and the development of interventions that are able to reduce the risk of recidivism among judicialized persons (Bonta and Andrews 2024). Meta‐analyses have shown that correctional interventions not based on risk‐need‐responsivity principles are generally ineffective and can sometimes lead to iatrogenic effects (Bonta and Andrews 2024; Hanson et al. 2009). By contrast, interventions that respect all three principles can achieve effect sizes comparable to those of psychological and medical interventions (Bonta and Andrews 2024; Marshall and McGuire 2003). The risk‐need‐responsivity principles of effective correctional intervention are rooted in reliable and valid assessments of the risk posed by judicialized individuals—particularly in the sources or causes of that risk (i.e., individuals' criminogenic needs). Even though the lessons of correctional psychology cannot be imported “as is” in the field of PVE, they nevertheless highlight the importance of effective risk assessment tools to help structure prevention and intervention efforts.

Unfortunately, as of now, there are no gold standards in the risk assessment of violent extremism. Multiple authorities in the field are critical of the viability of risk assessment, especially when it is of the actuarial type^2^ (Borum 2015; Corner and Taylor 2023; Monahan 2012; Sarma 2017). Five obstacles are commonly mentioned: (1) empirical research on risk and protective factors of violent extremism is not sufficiently developed to provide a sound empirical basis for what to include and not include in tools; (2) research on risk factors comes from commonalities between individuals who have committed terrorist attacks, but appropriate validation would require that these characteristics be relatively absent of control groups that have not committed such attacks; (3) the low base rate of recidivism among violent extremist offenders complicates predictive validity analyses and inflates the risk for false positives; (4) violent radicalization trajectories can lead to multiple outcomes (i.e., radicalization of ideas, joining and participating in extremist group activities, committing acts of violence, etc.) and the same predictors might not apply to the same outcomes; and (5) risk of violent extremism might not be cumulative, in contrast to risk of general violence or criminal recidivism (Borum 2015; Conley 2019; Monahan 2012; RTI International 2018; Sarma 2017; van der Heide et al. 2019).

To overcome these limitations, scholars have advocated for the development, validation, and use of structured professional judgment (SPJ) protocols over actuarial scales to assess the risk of violent extremism (Borum 2015; Monahan 2012). At present, most publicly known tools that are designed to assess the risk of violent extremism and that are used operationally by practitioners consist of SPJ protocols (Scarcella et al. 2016): the ERG22+ (National Offender Management Service 2011); the IVP guidance (Egan et al. 2016); the MLG‐V2 (Cook et al. 2013, 2015); the TRAP‐18 (Meloy 2017); and the VERA‐2R (Pressman 2009; Pressman et al. 2016; Pressman and Flockton 2012).

Although these tools are grounded in sound conceptual frameworks, concerns have long been raised about their empirical validation. A key reference in this regard is the systematic review by Scarcella et al. (2016), which reflected the state of the field at the time by highlighting the absence of predictive validity analyses—arguably the most important criterion for evaluating risk assessment tools. While some studies compared the scores of attackers and non‐attackers (e.g., Meloy and Gill 2016), they often relied on convenience samples and retrospective coding of open‐source information rather than prospective designs with real‐world participants. Scarcella et al. (2016) also noted that several widely used tools, including the VERA, had only been evaluated in terms of face/content validity and interrater agreement.

That being said, research in the field of PVE is evolving quickly, making assumptions that were considered “true” 10 years ago not so clear‐cut today. For example, most of the authors critical of violent extremism risk assessment cited the lack of empirical research on risk and protective factors and the differential predictive validity of factors depending on the outcome of interest. On that, a recent meta‐analysis of risk and protective factors of violent radicalization/extremism (Wolfowicz et al. 2020) included aggregated effect sizes separated by the following outcomes: radical attitudes, intention to act, and violent extremist behaviors (e.g., attacks). Results indicated that not only did similar risk and protective factors predict both attitudes and behaviors, but also that sociodemographic characteristics had less explanatory power than psychological‐ and personality traits‐related factors commonly found in violence risk assessment. This finding contradicts many assumptions held in the field, namely that risk factors for general violence alone may be insufficient or irrelevant in cases of violent extremism. Similarly, the assumption that risk of violent extremism is not cumulative has been challenged by studies that found risk and protective factors had incremental validity in the prediction of violent extremist attacks towards persons (Jensen and LaFree 2016). Hence, risk tools for violent extremism that were created 10 years ago might rely on evidence that is now being challenged, as data increasingly accumulates on types of violent radicalization processes beyond Islamist extremism. Similarly, the level of validation of some risk tools, as reviewed by Scarcella et al. (2016), might have significantly evolved since these authors published their systematic review. This rapid evolution of scientific knowledge of tools to assess and monitor factors relevant to risk of violent extremism warrants the production of a new systematic review, which will examine whether currently available tools are sufficiently reliable and valid to recommend their use, depending on the context, type of case assessed, and practitioner conducting the assessment.

#### 2.1.2 Why It Is Important to Do the Review

The current systematic review will be of major relevance for PVE practitioners receptive to the idea of using risk assessment tools but unsure of which to choose and for which context. Surveys of practitioners working in the field have noted that many are open to using tools but uncertain if the available ones were adapted to their sector, setting, or clients (Hassan et al. 2020; Madriaza et al. 2018). Furthermore, the lack of validation of most tools, as well as their significant monetary costs, has led some teams to create internally developed scales or rely purely on professional judgments. Knowing the potential pitfalls of assessing risk using unstructured clinical judgment (Dawes et al. 1989; Grove et al. 2000; Viljoen et al. 2025), it would be wise to answer practitioners' questions concerning risk tools for violent extremism. Our systematic review could also enable assessors to avoid the potential iatrogenic effects associated with the use of tools that are not fit for purpose for certain populations or contexts.

The costs associated with misevaluating risk are numerous. Risk overestimation can lead to more surveillance, stigmatization, unjustified repressive practices, longer than necessary sentences, and waste of funds on interventions that are not only unnecessary but also potentially harmful (Bonta and Andrews 2024; Brouillette‐Alarie and Lussier 2018). Risk underestimation, in turn, can result in premature releases and new victims (Douglas et al. 2017; Gendreau et al. 1996; Hanson 2009). Even though it is unrealistic to assume that each recidivism case could have been prevented with better assessment or decision‐making, it is important for clinicians, practitioners, and evaluators to be able to attest that their decisions were based on empirically validated procedures and high ethical standards in risk assessment.

Potential risk overestimation is especially important in the context of violent extremism because base rates are so low compared to other types of outcomes such as criminal recidivism (Borum 2015; Sarma 2017). This makes prediction especially challenging, as statistical models usually underperform when base rates are very low. Therefore, an investigation of the potential false positives of violent extremism risk tools is paramount.

In sum, the current systematic review could have implications for practitioners and decision‐makers in the field of PVE, as well as for public safety and judicialized persons. Evidence permitting, it will advise practitioners and deciders concerning which tools to use, which tools to avoid, and in which context. It will also potentially ease clinicians' concerns regarding risk tools or, conversely, raise their vigilance towards tools that are problematic. In both cases, the endeavor should contribute to better assessments of risks in the field of PVE.

#### 2.1.3 How This Review Might Inform or Supplement What Is Already Known in This Area

Searches for existing relevant systematic reviews and meta‐analyses were conducted on Google. Five systematic reviews (Desmarais et al. 2017; Gill et al. 2021; Lösel et al. 2018; Misiak et al. 2019; Vergani et al. 2020) and two meta‐analyses (Emmelkamp et al. 2020; Wolfowicz et al. 2020) on risk and protective factors for violent extremism were found. Although these systematic reviews and meta‐analyses were of relevance when examining the face and content validity of risk tools, they were beyond the scope of our systematic review, which focuses on tools rather than individual factors.

Two systematic reviews on tools that assess the risk of violent extremism were found: Scarcella et al. (2016) and Clesle et al. (2025). The first explored the level of psychometric validation of instruments that identify risk factors of terrorism, extremism, radicalization, authoritarianism, and fundamentalism (Scarcella et al. 2016). The authors found four instruments that specifically assess violent extremism risk (e.g., VERA) and 17 research measures/instruments that assess attitudes related to violent extremism (e.g., Religious Fundamentalism Scale [RFS], Right‐Wing Authoritarianism Scale [RWA]; Altemeyer and Hunsberger 1992). The authors concluded that the empirical validation of most scales, especially those designed specifically to assess the risk of violent extremism, was in its infancy. Even though they concluded that more validation was needed, they did not provide recommendations for practitioners who could be looking to use violent extremism risk assessment tools. Moreover, some risk tools (e.g., MLG) were omitted, while others may have been published since then. Considering the fast pace with which the field is evolving (i.e., many studies on violent extremism risk tools have been published in the last 9 years), proposing recommendations based solely on evidence collected in 2016 may no longer be current or reliable. Finally, Scarcella et al. (2016) only briefly discussed criterion‐validity findings (e.g., predictive, concurrent, and convergent validity)—arguably the most important validation criteria for professionals looking for guidelines on which tools to use and with whom.

Clesle et al. (2025) subsequently published a systematic review of violent extremism risk assessment tools at roughly the same time as the present study, reflecting the growing recognition of the need to synthesize evidence in this field. Their review offers a valuable overview of available instruments and highlights several conceptual and practical challenges faced by evaluators in the PVE space. While highly relevant, their synthesis focuses on summarizing the available information associated with each tool individually, including instruments for which no psychometric validation studies were available, explaining why the two reviews do not entirely overlap in the set of tools and studies covered. The present Campbell review, in contrast, applied stricter inclusion criteria, retaining only studies that reported empirical data on the psychometric properties of tools. In addition, their review did not include (or declare) a formal risk of bias assessment or conduct quantitative pooling of results. The present review applies standardized risk of bias procedures and reports meta‐analytic estimates where possible, while adopting a cross‐cutting approach that synthesizes psychometric properties across tools and validation domains.

Finally, although it is neither a systematic review nor a meta‐analysis, the contribution of Logan and Lloyd (2019) must also be noted. In their paper, the authors first contextualize the tasks of assessing, understanding, and managing risk in the field of PVE based on the progress made in the fields of correctional psychology and criminology. Then, they map the risk assessment tools used by PVE practitioners, describe their intended use, and relate studies attesting to their validation. Finally, they suggest guidance on how to ethically conduct risk assessment with individuals on violent radicalization trajectories, appraise the quality of existing evidence concerning the validation of available risk tools, and plan future evaluations of risk tools in the field. A similar effort can be found in the Extremism Risk Assessment Directory (Lloyd 2019), which presents concise, practitioner‐oriented summaries of multiple tools, including their intended use, structure, and supporting evidence. However, like most summaries of violent extremism risk tools (e.g., Conley 2019; RTI International 2018; van der Heide et al. 2019), the methods used to search for relevant literature or collect and analyze data were not systematic. Therefore, the current systematic review aims to improve on their important work by structuring data collection and analysis, as well as ensuring that the literature search is up to date.

### 2.2 Objectives

The main objective of this project was to gather, critically appraise, and synthesize evidence about the psychometric properties of tools used to assess the risk of violent extremism. The specific questions of our systematic review were as follows:

1. What are the tools used to assess the risk of violent extremism?
2. What is the reliability of these tools?
3. What is the validity of these tools?
4. Based on the tools' psychometric properties, as validated with specific populations and in specific contexts, are they fit for purpose for such populations and contexts? In other words, what are the advantages and disadvantages associated with the use of these tools for public safety, practitioners, and individuals on a trajectory towards violent extremism?

## 3 Methodology

This study followed a protocol approved by the Campbell Collaboration (Hassan et al. 2022).

### 3.1 Criteria for Considering Studies in This Review

The inclusion and exclusion criteria that were used to identify eligible studies of risk tools for violent extremism can be found in Table 1.

**Table 1:** Inclusion and exclusion criteria.

Included	Excluded
Risk tools designed to assess the risk of violent extremism	Risk tools from other fields intended for other purposes
Tools operationally usable by clinicians for cases involving individuals	– Research scales not operationally usable by clinicians – Tools for risk that do not involve individuals (e.g., risk of a building being targeted) – Tools requiring large social media databases – Self‐report questionnaires about extremist attitudes (e.g., RWA)
Primary data, including indirect primary data (e.g., triangulation of publicly available data)	Secondary data (i.e., literature reviews, systematic reviews, meta‐analyses—references were checked, however)
Quantitative studies	– Qualitative studies – Non‐empirical papers (e.g., theoretical papers or opinion pieces)
Comprises data on the types of reliability and validity eligible in this systematic review (see the Outcomes section)	Does not comprise data on eligible types of reliability and validity (see the Outcomes section)

We included studies with primary data resulting from the quantitative examination of the reliability and validity of tools used to assess the risk of violent extremism. We initially planned to include qualitative research designs, but an overview of initial search results indicated that very few or none were available in the context of risk tool validation. Furthermore, only tools usable by practitioners aiming to assess the risk of individuals were eligible. This means that the following were ineligible: (a) tools designed to assess the risk of a building being targeted; (b) tools that operate at a group level only; (c) tools that require access to large databases to function; (d) scales developed for theory validation only; and (e) self‐report questionnaires assessing constructs related to violent extremism.

Beyond limiting ourselves to studies comprising primary quantitative data (including the quantitative sections of mixed‐method studies) about the reliability and validity of eligible risk tools, we did not impose any restrictions on study design, type, method, or date (up to December 31, 2021) because the state of the literature is such that doing so could lead to the inclusion of only a small number of studies that do not give a clear picture of what is being done in the field. We excluded manuscripts where the authors reflected on a tool, as such reflections do not comprise primary empirical data. We did, nevertheless, take note of the authors' main conclusions, should they prove relevant for our discussion. The same was done for qualitative studies.

To reduce “publication bias” (Tanguy et al. 2014), our review included not only articles published in peer‐reviewed journals but also gray literature found by searching the Web. Our review excluded systematic and literature reviews, as these constitute secondary data. We did, however, harvest the references of such reviews to ensure that our search strategy found all the relevant studies.

### 3.2 Types of Studies

Eligible studies were categorized into the following groups (in decreasing order of methodological robustness):

1. Prospective data on reliability and validity using samples recruited in clinical and/or prison settings;
2. Retrospective data on reliability and validity using samples recruited in clinical and/or prison settings; or
3. Retrospective data on reliability and validity using samples built by compiling publicly available information about individuals involved in violent extremism (e.g., already existing terrorist databases or datasets put together by compiling journal articles about terrorist cases).

### 3.3 Population and Context

To be eligible for this review, studies needed to be about assessment tools designed to assess the risk of violent extremism. Violent extremism is here defined as the endorsement or use of violence in support of political, ideological, or religious causes (Neumann 2013; Schmid 2013, 2014). While violent extremism is sometimes conflated with radicalization, we distinguish between the two insofar as radicalization refers to a process—often nonlinear and individualized—through which a person may or may not come to justify or support violence (Borum 2011; Neumann 2013; Schmid 2013, 2014). In contrast, violent extremism refers to the potential outcome of that process, manifested through violent behavior (including terrorism; Schmid 2012) or explicit support for its use.

In the context of this systematic review, all included risk tool validation studies used outcomes involving actual or intended acts of extremist violence (e.g., planning, attempts, convictions), rather than the mere presence of radical or extremist attitudes. As such, the validation focus was clearly behavioral. Throughout the review, we aim to use the term “violent extremism” to refer to this behavioral outcome, and reserve “radicalization” for instances where we explicitly discuss processual or attitudinal dimensions. That said, we acknowledge the broader lack of definitional clarity in the field, a challenge repeatedly noted in the literature (Bartlett and Miller 2012; Neumann 2013; Schmid 2013, 2014). The terms violent extremism and radicalization are often used interchangeably in both academic and policy literatures, with terminological choices sometimes shaped more by political context or institutional trends than by analytic precision.

To be eligible, studies needed to comprise samples of individuals evaluated on a PVE risk tool because of their potential or actual involvement in extremist violence. Assessments could be conducted prospectively or retrospectively, by either practitioners or researchers. In the context of our systematic review, “risk” refers to the presence of multiple risk factors and/or the absence of protective factors, as defined by the tool. Participants across all levels of the risk spectrum were eligible, as this variation is necessary to adequately test predictive validity. Since most PVE tools follow a SPJ model, risk could be determined either through summative scores (often used in research) or through professional judgments guided by the tool's framework.

PVE risk tools can be used in tertiary prevention settings, that is, with individuals who have already committed acts of extremist violence or been involved with extremist groups and are now in the process of disengagement and reintegration. In such contexts, the relevant outcome would shift from the occurrence of extremist violence to the risk of recidivism, either in the form of further extremist violence or broader criminal reoffending. However, our review found no validation studies in which this type of outcome was examined using a prospective design. As such, while this use of PVE tools is conceptually possible and practically relevant, it was not reflected in the empirical evidence base available at the time of this review.

Finally, if a study only comprised practitioners or stakeholders rather than clients (e.g., a study asking evaluators about their experience using a tool), it was eligible on the condition that the tool was designed to assess the risk of violent extremism. Other than that, no other exclusion criteria concerning participants and contexts were applied.

### 3.4 Outcomes

In this review, the main outcome of interest was the level of psychometric evidence for each tool. Psychometric evidence was divided into the following dimensions: reliability (interrater agreement, internal consistency) and validity (content, convergent, discriminant, predictive, construct). Readers seeking to familiarize themselves with psychometric principles and their application to risk tool validation can consult Furr (2021) and Hanson (2021), respectively.

**Reliability**: Degree to which the measure of a construct is consistent or dependable.

1. *Interrater agreement*: Measure of consistency between two or more independent raters (e.g., assessor) of the same construct.
2. *Internal consistency*: The extent to which items within a scale are correlated with one another, indicating that they reflect the same underlying construct.

**Validity**: Extent to which a measure adequately represents the underlying construct it is supposed to measure.

1. *Content:* Whether the operationalization of a construct adequately covers its content.
2. *Construct*: Construct validity can be construed as an overarching type of validity that is defined by the extent to which scores on the instrument are indicative of the theoretical construct. In the context of this systematic review, construct validity will mainly encompass examinations of the latent structure of a tool as obtained by, for example, factor analyses.
3. *Convergent*: Closeness with which a measure relates to (or converges on) other measures of the same or closely related constructs.
4. *Discriminant*: Degree to which a measure does not measure (or discriminate from) other constructs it is not supposed to measure. In the context of this systematic review, studies of discriminant validity primarily involved testing group differences in risk scores, such as comparisons between individuals adhering to different ideological motivations or between lone and group‐based actors.
5. *Predictive*: Degree to which a measure successfully predicts a future outcome that it is theoretically expected to predict. *Note*: Several studies in this review used retrospective designs, where the outcome was already known at the time of assessment. While such designs can provide preliminary indications of predictive potential, they do not constitute true predictive validity tests because they cannot demonstrate the capacity of a tool to forecast future outcomes. In line with terminology used by authors, we refer to these as “postdictive” validity studies.

### 3.5 Search Methods for Identification of Studies

In consultation with a library science expert, we developed a search strategy aimed at targeting an array of bibliographic databases and gray literature resources. The bibliographic search was conducted in three phases. The first one was done at the end of 2020 and included studies published up to November 2020. At the time, we did not use proximity indicators and included self‐report questionnaires such as the RWA or the Activism and Radicalism Intention Scales (ARIS; Moskalenko and McCauley 2009).

Second, according to suggestions made by Campbell Collaboration editors and reviewers, modifications were integrated into the search strategy. Proximity indicators were added, some keywords were slightly modified, and self‐report questionnaires were excluded from the review. Most importantly, the search period was extended to include papers published until December 31, 2021.

Third, during the second half of 2022, Public Safety Canada asked our team to translate the search strategy into German to make use of databases specific to the German context. Thus, two researchers from Germany were recruited to our team, translated the search strategy, compiled a list of known German tools on which there might be evaluation research, and conducted official and gray literature searches. The German literature search used the same parameters as the second phase of our literature search, albeit in German and with risk tools that are specific to the German context. The end date remained December 31, 2021.

#### 3.5.1 Electronic Searches

We conducted searches in a variety of bibliographic databases, both subject‐specific databases and general multidisciplinary databases. While the searches employed standard Boolean logic, they were tailored to the features of each database, making use of available controlled vocabulary and employing proximity operators where possible. The databases used are as follows:

- Academic Search Complete (EBSCO).
- Criminal Justice Abstracts (EBSCO).
- Education Source (EBSCO).
- Education Resources Information Center (ERIC; EBSCO).
- Krimdok (https://krimdok.uni-tuebingen.de/Search/Advanced).
- Medline (EBSCO).
- National Criminal Justice Reference Service (NCJRS; ProQuest).
- ProQuest Central (ProQuest).
- ProQuest Dissertations and Theses Global (ProQuest).
- PsycINFO (Ovid).
- Sociological Abstracts (ProQuest).
- Web of Science platform's core collection:
  ◦ Science Citation Index Expanded.
  ◦ Social Sciences Citation Index.
  ◦ Arts & Humanities Citation Index.
  ◦ Conference Proceedings Citation Index – Science.
  ◦ Conference Proceedings Citation Index – Social Science & Humanities.
  ◦ Emerging Sources Citation Index.

Our search strategy is divided into three blocks. The first block is a proximity search for terms that represent “risk” and “assessment,” which needed to be a maximum of three words apart. The second block is the “extremism/radicalization” and accompanying synonyms block. The third block comprises the names of risk tools relevant to the field of PVE that were found in preliminary searches. To be captured by the search, a manuscript needed to satisfy (BLOCK 1 *AND* BLOCK 2) *OR* BLOCK 3.

Searches were conducted in English and German, but no languages were excluded from the results. The search fields included title, abstract, keywords, and subject/indexing. Endnote was used to store the search results. The full search record can be found in Appendix A, along with the detailed logs of all official literature searches (listed by search phase).

#### 3.5.2 Searching Other Resources

The gray literature search strategy was divided into three parts. Part 1 consisted of inputting the following search string in Google: “risk assess AND (radicalization OR radicalization OR extremism) filetype:pdf.” Google was preferred over Google Scholar to ensure a better coverage of manuscripts not published in indexed scientific journals. Search results were scoured until five pages of irrelevant results came up (with 10 results per page). We also conducted Google searches using the names of each violent extremism risk tool, including German tools. An example of a search string for the ERG22+ was: “extremism risk guidance filetype:pdf.”

Part 2 involved searching webpages, research repositories, and non‐indexed journals relevant to violent radicalization/extremism. These sources were examined using variants of the Google search strings, including those containing the names of PVE risk tools. The list of webpages, repositories, and journals was based on another Campbell Collaboration systematic review led by members of this team and on suggestions made by our German colleagues. The list is as follows:

- Alliance for Peacebuilding—P/CVE Digest Resource Library: https://www.allianceforpeacebuilding.org/cve-digest-master-sources-list/category/CVE%20Digest?tag=Reports%20and%20Research.
- American Bar Association—Rule of Law Initiative: https://www.americanbar.org/advocacy/global-programs/what-we-do/resources/.
- Brennan Center for Justice: https://www.brennancenter.org/our-work/research-reports/.
- Campbell Collaboration: https://www.campbellcollaboration.org/evidence/.
- Center for Evidence Based Crime Policy: https://cebcp.org/.
- Center for Strategic and International Studies: https://www.csis.org/analysis?f%5B0%5D=content_type%3Areport&f%5B1%5D=report_type%3A3034.
- Center on International Cooperation: https://cic.nyu.edu/resources/.
- CLEEN Foundation: https://cleen.org/.
- *Comité interministériel de prévention de la délinquance et de la radicalization*: https://www.cipdr.gouv.fr/ressources-pratiques/.
- Council of Europe—Counter‐terrorism: https://www.coe.int/en/web/counter-terrorism.
- COWI: https://www.cowi.com/reports-and-publications/publications/.
- Defense: https://defence.gov.au/.
- Department of Homeland Security: https://www.dhs.gov/publications.
- Educate Against Hate: https://www.educateagainsthate.com/resources/.
- Geneva Center for Security Policy: https://www.gcsp.ch/publications.
- Georgetown Security Studies Review: https://georgetownsecuritystudiesreview.org/.
- Global Center on Cooperative Security: https://globalcenter.org/directory/.
- Global Counterterrorism Forum: https://www.thegctf.org/.
- GOV.UK: https://www.gov.uk/search/research-and-statistics.
- Hedayah: https://hedayah.com/resources/.
- Institute for Security Studies: https://issafrica.org/.
- Institute for Strategic Dialog: https://www.isdglobal.org/isd-publications/.
- International Center for Counter‐Terrorism: https://www.icct.nl/publications.
- International Center for the Study of Radicalization: https://icsr.info/.
- International Crisis Group: https://www.crisisgroup.org/.
- Journal for Deradicalization: https://journals.sfu.ca/jd/index.php/jd/index.
- KrimLit (German): https://allegro.wwwan.de/cgi-bin/krimz/maske.pl?db=krimz&lang=en.
- KrimPub (German): https://krimpub.krimz.de/home.
- *Ministère de l'Intérieur*: https://www.interieur.gouv.fr/Publications.
- Ministry of Foreign Affairs of Denmark: https://um.dk/en.
- National Consortium for the Study of Terrorism and Responses to Terrorism: https://www.start.umd.edu/publications.
- Organization for Security and Co‐operation in Europe: https://www.osce.org/resources/publications.
- PSYNDEX (German): https://psyndex.de/en/.
- Public Safety Canada: https://www.publicsafety.gc.ca/cnt/rsrcs/pblctns/index-en.aspx.
- Publications Office of the European Union: https://op.europa.eu/en/home.
- RAND—Homeland Security and Public Safety: https://www.rand.org/topics/homeland-security-and-public-safety.html.
- Royal United Services Institute: https://rusi.org/explore-our-research/publications.
- Search for Common Ground: https://www.sfcg.org/reports-evaluations-publications/.
- Social Science Open Access Repository: https://www.gesis.org/en/ssoar.
- UNESCO—Preventing Violent Extremism: https://en.unesco.org/preventing-violent-extremism.
- United Nations Development Program: https://www.undp.org/publications.
- United Nations Office on Drugs and Crime—Terrorism Prevention Branch: https://www.unodc.org/unodc/en/terrorism/index.html.
- United States Agency for International Development: https://www.usaid.gov/.
- Violent Extremism Evaluation Measurement Framework: https://www.rand.org/randeurope/research/projects/violent-extremism-evaluation-measurement-framework-veem.html.
- Violence Prevention Network: https://violence-prevention-network.com/tools-resources/.

Part 3 consisted of thoroughly searching the reference sections of literature and systematic reviews of risk assessment tools in the PVE space (Lloyd 2019; Logan and Lloyd 2019; RTI International 2018; Scarcella et al. 2016; van der Heide et al. 2019), as well as the references of all included studies in this systematic review.

The gray literature searches of Phase 1 were conducted in February 2021, those of Phase 2 in November 2023, and those of Phase 3 in February 2023. The eligibility end date for both official and gray literature was December 31, 2021. We did not contact authors or organizations to obtain studies beyond those identified in the aforementioned searches, nor did we contact them to obtain missing or supplementary data.

### 3.6 Data Collection and Analysis

#### 3.6.1 Selection of Studies

Selecting admissible studies was performed by three research assistants who independently screened the abstracts of manuscripts identified by the systematic search. Each manuscript was rated as “no,” “maybe,” or “yes” by at least two research assistants, according to a screening tool based on our inclusion/exclusion criteria (see Hassan et al. 2022). Disagreements (i.e., one research assistant having a different response than the others) were dealt with in a meeting with lead researchers until consensus was reached.

Training was provided to research assistants before the start of the selection process. After the first 400 articles (8.6% of the records identified in the November 2020 search) were coded, the “initial” interrater agreement was computed using Fleiss' (1971) kappa and reached 0.468 (SE = 0.029). Because that result was problematic, another round of training was provided to research assistants before computing the “final” interrater agreement on another set of articles (the Web of Science results, which amounted to 18.5% of the November 2020 search records). The interrater agreement of that round reached a Fleiss's kappa of 0.746 (SE = 0.020), which indicates moderate agreement (Shrout 1998).

All studies rated as “maybe” or “yes” after disagreements were resolved were selected for full‐text screening. Full‐text screening was also conducted independently by two research assistants. During this stage, assistants confirmed that the studies met all the inclusion/exclusion criteria and comprised data relevant to our outcomes of interest—something that is not easy to determine at the initial screening stage. Each full‐text study marked for exclusion was also screened by lead researchers to ensure that no relevant data was left out of the systematic review.

#### 3.6.2 Data Extraction and Management

Each retained study was processed in an Excel coding sheet, based on a coding manual (see Appendix B), in which three research assistants extracted the following information:

1. Document ID, title, authors, year of publication, and place of publication.
2. Relevant risk tools studied in the paper.
3. Data source (meetings with participants, private institutional records, triangulation of publicly available data).
4. Study design (cross‐sectional vs. longitudinal, retrospective vs. prospective).
5. Sample characteristics (*N*, gender, age, country, ethno‐racial group, education, employment status, religious/ideological affiliation).
6. Outcomes
  a. Reliability (interrater agreement, internal consistency).
  b. Validity (content, construct, convergent, discriminant, predictive/postdictive).
7. Recommendations of authors (concerning the tool, for practitioners, for future research).
8. Limitations mentioned by the authors.
9. Coder ID and coding date.

Data extraction was conducted by a single research assistant per study. However, all coding sheets were thoroughly reviewed and verified by lead researchers before being entered into the summary of evidence tables, ensuring consistency and accuracy. If a study assessed the psychometric properties of multiple eligible tools, it was listed in multiple rows (once per tool). If relevant data could not fit in the predetermined structure of the coding sheet, it was added in an “other relevant info” column.

#### 3.6.3 Assessment of Risk of Bias in Included Studies

Risk of bias was assessed through a modified and shortened version of the COSMIN Risk of Bias checklist (Mokkink et al. 2018; Prinsen et al. 2018; Terwee et al. 2018). Because the COSMIN Risk of Bias checklist was created for the medical field, trying to use it “as is” in an emergent field such as that of PVE would make the tool unfit for purpose (i.e., too many items would be rated N/A due to lack of information). Therefore, the lead researchers, who possess substantial expertise in psychometry and quantitative research, conducted a comprehensive review of the full COSMIN Risk of Bias checklist. Through a series of meetings, they systematically evaluated each item in relation to the types of study designs typically found in the validation literature on violent extremism risk assessment tools. Items deemed systematically inapplicable—such as those requiring experimental manipulation, responsiveness testing, or large, randomly sampled cohorts—were excluded from the adapted version. The objective was to retain only those COSMIN items capable of meaningfully differentiating between stronger and weaker evidence, given the methodological constraints and empirical realities of the PVE field. The final checklist emphasized the clarity of the tool's intended use (target population, construct being measured, context of use), followed by general methodological features (sample size adequacy, appropriateness of the study design, suitability of data analysis methods). It then included items related to each type of reliability and validity assessed in this systematic review. The modified version of the COSMIN tool can be found in Appendix C.

The risk of bias checklist used in this systematic review was scored by research assistants under the supervision of lead researchers. Training in psychometry and quantitative research was provided to research assistants beforehand. Research assistants were encouraged to ask for help with any item that might prove difficult to score. When this happened, the lead researchers made sure to review the risk of bias checklist of the study in question.

We did not exclude studies based on their methodological design a priori because one of the main goals of this systematic review was to critically appraise and provide guidance on the level of validation of risk tools in the PVE field. If less robust designs (e.g., retrospective rather than prospective) were to be excluded from this review, it would present a picture of the literature that is artificially optimistic.

#### 3.6.4 Assessment of Certainty of the Evidence

Certainty of the evidence was assessed through both the modified COSMIN Risk of Bias checklist and the overall robustness of the methodological design. Robustness was rated on a 3‐point scale inspired by Helmus and Babchishin (2017) and the types of studies we anticipated to find in our search: (1) below average (retrospective data obtained in convenience samples), (2) average (retrospective data obtained in non‐convenience samples), or (3) robust (prospective data obtained in non‐convenience samples). Conclusions of studies were discussed according to the strength of the evidence presented. COSMIN scores for all eligible studies can be found in Appendix C. Assessment of the certainty of the evidence was rated by the lead researchers.

#### 3.6.5 Measures of Psychometric Qualities

This review focused on synthesizing psychometric indicators of reliability and validity. For predictive/postdictive validity, studies reported a range of effect size metrics, including Cohen's *d*, area under the ROC curve (AUC), and raw group differences (means and standard errors) between individuals who did and did not engage in violent extremist behavior. To allow for comparability and meta‐analysis, we transformed all predictive/postdictive validity estimates into point‐biserial correlations (*r*) because they offer interpretability, comparability across study designs, and compatibility with the Comprehensive Meta‐Analysis (CMA) software used in this review. Effect sizes conversions followed Rice and Harris's (2005) guidelines for recidivism studies. When only group means and standard errors were available, we first calculated Cohen's *d* and then converted it to *r*.

We intended to meta‐analyze interrater agreement following Sun's (2011) guidelines for Cohen's kappa, but the lack of standard error/percent of agreement reporting in kappa studies made this analysis impossible. No other psychometric properties were meta‐analyzable in this review.

#### 3.6.6 Unit of Analysis Issues and Determination of Independent Findings

In this review, the unit of analysis was a single predictive/postdictive validity estimate per study—specifically, the association between the total score of the risk assessment tool and the outcome of interest. Most estimates were derived from postdictive analyses (i.e., outcomes had occurred before assessment). We did not identify studies with multiple effects for an outcome, so no statistical adjustments (e.g., robust variance estimation) were necessary.

To maintain statistical independence, we excluded estimates based on dimensional or subscale scores, even when available, as including them alongside total scores would have introduced multiple effect sizes from the same sample. Although we would have considered subgroup‐specific estimates (e.g., by gender or ideology), none were reported—likely due to limited sample sizes in the original studies.

We also remained attentive to potential secondary reports of the same study. However, no overlapping reports were identified among the included studies. Had such secondary reports been found, we would have retained all versions for coding and documentation while selecting a single effect size per sample for synthesis based on completeness and methodological rigor.

#### 3.6.7 Dealing With Missing Data

We did not contact study authors to obtain missing or supplementary data, as outlined in our protocol and justified based on feasibility and resource constraints. When critical information (e.g., effect sizes, standard errors, or sample sizes) was missing and could not be derived from available statistics (e.g., means, group comparisons, or *p*‐values), the study was excluded from quantitative synthesis but retained in the narrative synthesis when relevant. No imputation procedures were applied.

#### 3.6.8 Assessment of Heterogeneity

Statistical heterogeneity among studies included in the meta‐analysis was assessed using the *Q*‐test, the *I*² statistic, and Tau‐squared (*τ*²). *I*² values of 25%, 50%, and 75% were interpreted as indicative of low, moderate, and high heterogeneity, respectively, following the guidelines of Higgins (2003). Tau‐squared provided an estimate of the between‐study variance in effect sizes and was included to complement *I*² by quantifying absolute heterogeneity. All heterogeneity statistics were computed using random‐effects models.

#### 3.6.9 Assessment of Reporting Biases

Due to the limited number of studies included in the meta‐analysis, we did not conduct formal statistical tests of publication bias, such as funnel plots or Egger's regression (Sterne et al. 2011), as these methods are underpowered and unreliable with fewer than 10 studies (Ioannidis and Trikalinos 2007). However, we qualitatively considered the potential for reporting biases by examining author affiliations/study funding and the presence of selectively reported outcomes.

#### 3.6.10 Data Synthesis

All quantitative syntheses were conducted using the CMA Version 4 software (Borenstein et al. 2022a). The analyses relied on each study's effect size (expressed as a point‐biserial correlation, *r*) and its corresponding sample size (*N*), which allowed for the calculation of standard errors and confidence intervals. Meta‐analyses were conducted only when two or more independent studies were available for the same tool and psychometric property.

A random‐effects model was used throughout, reflecting the assumption that effect sizes may vary across studies due to differences in design, populations, tools, and implementation contexts. This model accounts for both within‐ and between‐study variance and is recommended when methodological and contextual heterogeneity is expected (Borenstein et al. 2022b). For each meta‐analysis, 95% confidence intervals, standard errors, and heterogeneity statistics were computed. Forest plots were produced to visually display the results, including the individual study estimates and the pooled effect size.

In cases where studies reported perfect predictive/postdictive accuracy (e.g., AUC = 1.00), a continuity correction was applied to allow inclusion in the meta‐analysis. This involved slightly reducing the effect size (e.g., to AUC = 0.98) to avoid zero variance, which can produce infinite weights in random‐effects models (Sweeting et al. 2004). If the effect size metric was not already a correlation, the corrected value was then translated into a point‐biserial correlation using the procedures outlined by Rice and Harris (2005).

#### 3.6.11 Subgroup Analysis and Investigation of Heterogeneity

Our original plan was to use meta‐regression to investigate potential sources of heterogeneity, such as client ideology or evaluation setting. However, due to the limited number of studies, these analyses could not be conducted. As recommended by Borenstein (2019), meta‐regression generally requires at least 10 studies to yield reliable results—a threshold we did not meet.

#### 3.6.12 Sensitivity Analysis

We conducted a sensitivity analysis using CMA's “one study removed” procedure to detect and examine possible outliers. This method assesses whether the overall effect size is unduly influenced by any single study (Borenstein et al. 2022b). It requires at least three studies and was therefore applied only to the meta‐analysis of the TRAP‐18's postdictive validity. We inspected the results to determine whether the removal of any single study altered the statistical significance or substantially changed the magnitude of the pooled effect.

#### 3.6.13 Narrative Synthesis

When meta‐analysis was not feasible—typically due to only one eligible study per tool or incomplete statistical reporting—a narrative synthesis was conducted. In these cases, results were described qualitatively, highlighting reported effect sizes, methodological strengths or limitations, and relevance to the review's objectives. This approach allowed us to include valuable information from studies that could not be aggregated quantitatively.

#### 3.6.14 Treatment of Qualitative Evidence

This review did not include qualitative studies, in accordance with editorial guidance and the scope defined in the registered protocol. The focus was limited to studies reporting quantitative data on the psychometric properties of PVE risk assessment tools. As such, no qualitative evidence was synthesized or appraised.

## 4 Results

### 4.1 Results of the Search

**Figure 1:**
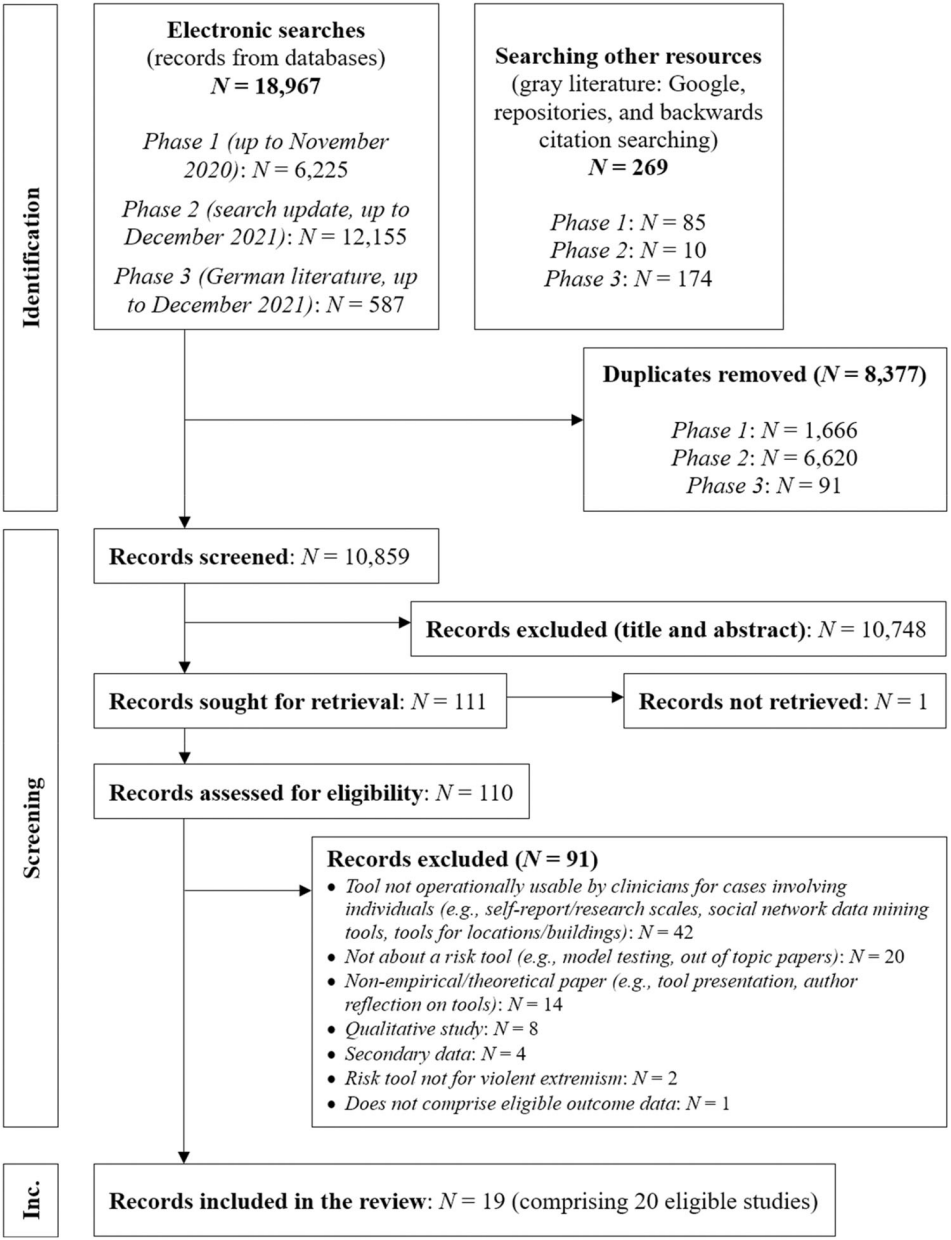
PRISMA statement.

The three phases of the search strategy led to the identification of 10,859 records. Of these, 10,748 were excluded following title and abstract screening. Of the 110 documents available for full‐text screening, one could not be retrieved using available library resources, 91 were excluded for not meeting one or more of the inclusion criteria established in our protocol. This led to a final selection of 19 documents comprising 20 studies, as the Hart et al. (2017) report contained two eligible studies (see Figure 1). All but one of these eligible were written in English; one was written in German.

### 4.2 Included Studies

The list of retained studies, along with their general characteristics, can be found in Table 2. These studies were published between 2013 and 2023. Manuscripts with a final publication date of 2022 or 2023 were in advance online publication status in December 2021 and, thus, eligible.

**Table 2:** Summary of the evidence.

Tools	Study	Data source	Sample (Adults)	Outcomes (Reliability)	Outcomes (Validity)	Postdictive validity criterion	Statistical analyses	Design robustness^a^
*Der Screener—Islamismus*	Böckler et al. (2017)	Official records	*N* = 31 (Islamist VE)	Interrater (*N* = 8 practitioners)	Content^b^	—	Gwet's AC1, number of items present per case, user satisfaction (Likert)	Average
ERG22+	Powis et al. (2019)	Official records	*N* = 50 (various ideologies but mostly Islamist VE)	Interrater (*N* = 35; 2 researchers, 33 practitioners)	—	—	Percent agreement, Cohen's weighted kappa, Fleiss' kappa, intraclass correlation	Average
ERG22+	Powis et al. (2021)	Interviews + official records	*N* = 171 (Islamist VE)	Internal consistency	Construct	—	Exploratory factor analysis, multidimensional scaling analysis, Cronbach's alpha	Average
IVP guidance	Egan et al. (2016)	Publicly available data	*N* = 182 (various ideologies)	Internal consistency, interrater (*N* = 2)	Discriminant	—	Cronbach's alpha, Cohen's kappa, ANOVA + post hoc, AUC, sensitivity/specificity	Below average
MLG‐V2	Hart et al. (2017) – Study 1	Publicly available data	*N* = 5 (various ideologies)^c^	Interrater (*N* = 4)	Convergent	—	Intraclass correlation, Pearson correlation	Below average
MLG‐V2	Hart et al. (2017) – Study 2	N/A	N/A	—	Convergent (*N* = 3 raters)	—	Degree of overlap between MLG‐V2 and VERA 2 items	N/A
TRAP‐18	Böckler et al. (2020)	Publicly available data	*N* = 80 (Islamist VE)	—	Postdictive	Committing extremist violence	Chi^2^, *t*‐test, ANOVA + post hoc, AUC	Below average
TRAP‐18	Brugh et al. (2023)	Publicly available data	*N* = 77 (Islamist VE)	Interrater (*N* unspecified)	Content, discriminant	—	Krippendorff's alpha, percent of present/absent/missing items, Chi^2^, *t*‐test	Below average
TRAP‐18	Challacombe and Lucas (2019)	Publicly available data	*N* = 58 (far right)	Interrater (*N* = 2)	Postdictive	Committing extremist violence	Cohen's kappa, Chi^2^, Cohen's *d*, logistic regression	Below average
TRAP‐18	Collins and Clark (2021)	Publicly available data	*N* = 1 (incel)	—	Content	—	Number of items present	Below average
TRAP‐18	Dmitrieva and Meloy (2022)	Publicly available data	*N* = 1 (far right)	—	Content	—	Number of items present	Below average
TRAP‐18	Erlandsson and Reid Meloy (2018)	Publicly available data	*N* = 1 (far right)	—	Content	—	Number of items present	Below average
TRAP‐18	Fernández García‐Andrade et al. (2019)	Interviews + official records	*N* = 44 (homeless persons with severe mental illness)	—	Postdictive	Committing extremist violence	Chi^2^, *t*‐test/Mann–Whitney's *U*, AUC	Average
TRAP‐18	Goodwill and Meloy (2019)	Publicly available data	*N* = 56 (various ideologies)^d^	—	Construct, postdictive	Committing extremist violence	Multidimensional scaling analysis, centroid analysis, *t*‐test	Below average
TRAP‐18	Kupper and Meloy (2021)	Publicly available data	*N* = 30 (various ideologies)	—	Content	—	Percent of present items, Chi^2^	Below average
TRAP‐18	Meloy and Gill (2016)	Publicly available data	*N* = 111 (various ideologies)	—	Discriminant, postdictive	Attack thwarted versus carried out	Percent of present items, Chi^2^	Below average
TRAP‐18	Meloy et al. (2015)	Publicly available data	*N* = 22 (various ideologies)	Interrater (*N* = 2)	Content, discriminant	—	Cohen's kappa, percent of present items, Chi^2^	Below average
TRAP‐18	Meloy et al. (2019)	Publicly available data	*N* = 56 (various ideologies)^d^	—	Postdictive	Committing extremist violence	Chi^2^, Odds ratio	Below average
TRAP‐18	Meloy et al. (2021)	Publicly available data	*N* = 125 (various ideologies)	—	Content (time sequencing)	—	Proximity coefficients	Below average
VERA	Beardsley and Beech (2013)	Publicly available data	*N* = 5 (various ideologies)^c^	Interrater (*N* = 2)	Content	—	Cohen's kappa, number of items present per case	Below average

*Note:* The “*N*” next to “interrater” indicates the number of raters that contributed to the interrater agreement analysis.

^a^ Overall robustness of the methodological design is rated as follows: robust (prospective data obtained in non‐convenience samples), average (retrospective data obtained in non‐convenience samples), or below average (retrospective data obtained in convenience samples).

^b^ Böckler et al. (2017) assessed content validity through item fit in 31 radicalization cases and practitioner‐rated face validity via a satisfaction survey.

^c^ Hart et al. (2017) rely on the same cases as Beardsley and Beech (2013).

^d^ Goodwill and Meloy (2019) and Meloy et al. (2019) rely on the same sample.

All of the studied tools but one were SPJ tools, with the sole actuarial tool being *Der Screener—Islamismus* (Böckler et al. 2017). There were 13 studies on the TRAP‐18, two studies about the ERG22+, two studies on the MLG‐V2, one study on the IVP guidance, one study on the VERA, and one study on *Der Screener—Islamismus*. The tools varied in length: the TRAP‐18 includes 18 items, the ERG22+ includes 22, the MLG‐V2 includes 16, the IVP guidance includes 16, the VERA includes 28, and *Der Screener—Islamismus* includes 13.

The mean number of participants in studies was 58.21 (SD = 55.14), with a total of 1106 participants across all studies. The largest sample size was 182 (Egan et al. 2016), and the lowest one was 1, as found in three case studies of the TRAP‐18 (Collins and Clark 2021; Dmitrieva and Meloy 2022; Erlandsson and Reid Meloy 2018). All studies focused on adult individuals susceptible to violent extremism, with no samples of children or adolescents. Seven studies included a small percentage (2%–14%) of female participants (Böckler et al. 2020; Challacombe and Lucas 2019; Egan et al. 2016; Goodwill and Meloy 2019; Meloy et al. 2015, 2019, 2021), but the rest comprised only men.

The ideologies represented across studies were surprisingly varied. In the case of the TRAP‐18, for example, there were approximately as many studies focused on religiously inspired violent radicalization as there were on far‐right extremism. However, an emphasis on jihadist extremism remains evident in the validation of certain tools—most notably the ERG22+ and *Der Screener—Islamismus*. In the case of the latter, this is to be expected: *Der Screener—Islamismus* is the only ideology‐specific tool documented in this systematic review, and its validation study inevitably involved individuals with jihadist backgrounds (Böckler et al. 2017). Finally, one study was about homeless persons with severe mental illness at risk of extremist violence (Fernández García‐Andrade et al. 2019).

All studies but three formed their sample through the triangulation of publicly available data, which has multiple implications regarding the risk of bias and certainty of the evidence. Only the ERG22+ studies (Powis et al. 2019, 2021), one TRAP‐18 study (Fernández García‐Andrade et al. 2019), and the *Der Screener—Islamismus* study (Böckler et al. 2017) used official records or interview data. Furthermore, none of the studies used prospective research designs. According to the criteria we outlined in the Certainty of the Evidence section, this means that no studies met the threshold for what we pre‐defined as a robust methodological design. Even though we anticipated such studies to be the exception rather than the rule, finding none in our review was notable and has numerous ramifications for PVE risk assessment practice, policy, and future research.

### 4.3 Excluded Studies

A total of 91 full‐text studies were excluded during the review process, based on predefined eligibility criteria. Reasons for exclusion are summarized in Figure 1 and detailed in Appendix D, which lists all excluded studies along with the primary reason for exclusion.

Four observations stemmed from these exclusions. First, many tools that were included in Block 3 of our search strategy did not have eligible quantitative studies attesting to their psychometric validation. Some of the evidence about these tools is known to be obscured from the public (i.e., internal publications; for an overview, see Lloyd 2019). In other cases, the tool was simply never assessed empirically. Second, a considerable portion of the excluded literature focused on self‐report scales such as the ARIS, RWA, or RFS, which aim to measure attitudes toward radicalization, authoritarianism, and fundamentalism, respectively. These studies often featured advanced psychometric work, including factor analyses, tests of convergent and cultural validity, and were commonly used in explanatory models testing correlates and predictors of violent extremism (e.g., Bhui et al. 2014). In fact, the psychometric literature on these research‐oriented scales may be more developed than that for some of the applied risk tools included in this review. However, since these instruments are not intended for use by practitioners conducting individual risk assessments, they were excluded.

Third, many excluded studies were tool presentation papers (e.g., Pressman 2016)—often falling into the nonempirical or secondary‐data categories—and nearly matched the number of included studies in this review. Such papers are notably documented in Clesle et al.'s (2025) systematic review. This reflects a broader trend in the PVE space, where the quantity of theoretical frameworks and systematic reviews often rivals the number of original empirical studies. Similar trends have been observed in research on PVE programming, where reviews frequently highlight the lack of robust primary data and call for stronger empirical foundations rather than additional syntheses drawing from the same limited evidence base (e.g., Brouillette‐Alarie et al. 2022, 2025).

Fourth, we believe that most studies were excluded because they fell outside the scope of the review rather than due to overly strict methodological standards. We adopted a deliberately inclusive approach for empirical studies—even those with notable design limitations—to capture the breadth of available evidence. Thus, the exclusions presented here do not distort the overall state of knowledge of PVE risk tools, but rather reflect the field's current limitations in terms of validated, practitioner‐oriented instruments.

### 4.4 Risk of Bias in the Included Studies

All included studies (*k* = 20) were appraised for risk of bias using a modified version of the COSMIN checklist (see Appendix C). This framework covered key aspects such as clarity in tool description, adequacy of sample size, and the appropriateness of study design and analytical methods. Because most tools (aside from the TRAP‐18) were represented by only one or two studies, comparisons of COSMIN scores across tools were not considered meaningful. Nor were domain‐level scores calculated, as most domains contained only one or two items, and many items were rated as not applicable (i.e., missing), resulting in domain scores that would have been nearly indistinguishable from individual item ratings. Nevertheless, we provide descriptive statistics (i.e., success rates) for each COSMIN item in Table 3, offering insight into common methodological limitations and areas for improvement in future psychometric evaluations of PVE risk assessment tools.

**Table 3:** Descriptive statistics of COSMIN checklist items and total score.

Domains/Items	Valid *N*	Percent of items succeeded or *M* (SD)
Risk tool presentation		
(1) Is a clear description provided of the construct assessed by the tool?	20	100.0%
(2) Is a clear description provided of the target population for which the tool was developed?	20	95.0%
(3) Is a clear description provided of the tool's context of use?	20	100.0%
Data analysis (general)		
(4) Was an appropriate approach used to analyze the data?	20	95.0%
(5) Was the sample size appropriate?	20	50.0%
(6) Were the design and statistical methodology of the study free of any significant flaws?	20	30.0%
If there were inter‐rater reliability analyses		
(7) For dichotomous/nominal/ordinal scores: Was kappa calculated?	8	87.5%
(8) For ordinal scores: Was a weighted kappa calculated?	3	66.7%
If there were internal consistency analyses		
(9) Was an internal consistency statistic calculated for each unidimensional scale or subscale separately?	2	50.0%
(10) For continuous scores: Was Cronbach's alpha or omega calculated?	1	100.0%
(11) For dichotomous scores: Was Cronbach's alpha or KR‐20 calculated?	1	100.0%
If there were face/content validity analyses		
(12) Was each item tested in an appropriate number of participants?	4	50.0%
(13) Was an appropriate method used to ask participants about the relevance of each item?	2	100.0%
(14) Was an appropriate method used to ask participants about the tool's comprehensiveness?	1	100.0%
(15) Was an appropriate method used to ask participants about the comprehensibility of the tool's instructions and items?	1	100.0%
If there were convergent validity analyses		
(16) Is it clear what the comparator instrument(s) measure(s)?	2	100.0%
(17) Was the statistical method appropriate for the hypotheses to be tested?	2	50.0%
If there were comparisons between groups (except cross‐cultural validity)		
(18) Was an adequate description provided of important characteristics of the subgroups?	8	75.0%
(19) Was the statistical method appropriate for the hypotheses to be tested?	8	100.0%
If there were concurrent/predictive validity analyses		
(20) For continuous scores: Were correlations or the area under the receiver operating curve calculated?	7	71.4%
(21) For dichotomous scores: Were sensitivity and specificity determined?	5	60.0%
If there were factor analyses		
(22) Was an exploratory or confirmatory factor analysis performed?	1	100.0%
(23) Was the sample size appropriate for factor analysis?	1	100.0%
If there were cross‐cultural (external) validity analyses		
(24) Were the samples similar for relevant characteristics except for the group variable?	1	100.0%
COSMIN total score (/100)		78.85 (14.67)

Items most consistently rated as “Yes” were those relating to the clarity of the construct assessed (Item 1), the identification of the target population (Item 2), and the definition of the tool's intended context of use (Item 3). These conceptual components were reported clearly in the majority of studies. In contrast, several items were frequently failed. The most commonly failed item was Item 6 (design/statistical flaws), which was rated “No” in 14 out of 20 studies (70%). Item 5 (sample size adequacy) was also problematic, failing in 10 studies (50%), underscoring a widespread issue with underpowered designs.

Low COSMIN total scores were most often attributable to small sample sizes. Sample size considerations appear across multiple COSMIN domains—including construct validity, reliability, and internal consistency—such that studies with limited samples often incurred repeated penalties. Detailed information about the risk of bias is discussed in each outcome section below, so that it may be contextualized by type of psychometric validation, for which appropriate versus inappropriate methods and designs differ.

### 4.5 Synthesis of Results

#### 4.5.1 Reliability

##### 4.5.1.1 Interrater Agreement

Results concerning interrater agreement can be found in Table 4. Meta‐analysis could not be performed because it requires both the kappa value and the standard error/percent of agreement (Sun 2011). In the context of this systematic review, only the TRAP‐18 had interrater agreement values coming from multiple studies, and those failed to provide standard errors or percentages of agreement.

**Table 4:** Summary of the evidence concerning interrater agreement.

Tools	Study	Context	Results^a^	Effect size qualification^b^
*Der Screener—Islamismus*	Böckler et al. (2017)	Eight practitioners with varying levels of experience blind‐coded six anonymized case summaries involving jihadist extremists from German‐speaking countries.	Mean Gwet's AC1 of 0.80 (can be interpreted in the same way as a Cohen's kappa).	Moderate
ERG22+	Powis et al. (2019)	1. Two researchers with 5+ years' experience in the use of the ERG22+ coded 50 cases. 2. 33 practitioners trained in the ERG22+ (divided between experienced and routine) coded two cases.	1. Mean “research” Cohen's weighted kappa of 0.93. 2. Mean “field” Fleiss' kappa of 0.47 for case 1 and 0.59 for case 2. Experienced clinicians had higher kappas for case 1 (0.55 vs. 0.29).	1. Substantial 2. Fair
IVP guidance	Egan et al. (2016)	Two raters blind‐coded 30 of the 182 cases (16%).	Mean Cohen's kappa of 0.80.	Moderate
MLG‐V2	Hart et al. (2017) – Study 1	Two raters coded the five cases.	Median intraclass correlation of 0.95.	Substantial
TRAP‐18	Brugh et al. (2023)	Raters coded 20 of the 77 cases (26%).	Krippendorff's alpha of 0.95.	Substantial
TRAP‐18	Challacombe and Lucas (2019)	Two raters (the lead author and a graduate student) coded the 58 cases.	Mean Cohen's kappa of 0.76.	Moderate
TRAP‐18	Meloy et al. (2015)	Two raters expert in the field coded the 22 cases.	Mean Cohen's kappa of 0.90.	Substantial
VERA	Beardsley and Beech (2013)	Two raters coded the five cases (the second rater was trained by the first).	Cohen's kappas were of 0.76 or greater.	Moderate to substantial

^a^ Kappas are reported for items, not dimensional or total scores. Some studies provided kappas for dimensional/total scores: Hart et al. (2017) and Powis et al. (2019).

^b^ According to Shrout (1998).

Interrater agreement was in the moderate to substantial range when coding was done in a research context by authors, research assistants, or experts in the field. Two studies assessed interrater agreement in conditions approximating routine use: Böckler et al. (2017) and Powis et al. (2019). In the latter, practitioners trained on the ERG22+ showed unsatisfactory agreement (below 0.60), raising concerns about its reliability in practice. By contrast, Böckler et al. (2017) found moderate (0.80) interrater agreement for *Der Screener—Islamismus* among users with varying levels of training and expertise. Therefore, while most studies report good interrater reliability for PVE risk tools, uncertainty remains as to whether these results generalize to routine, non‐research settings.

##### 4.5.1.2 Internal Consistency

This type of reliability was scarcely studied in violent extremism risk tools, even though most of them comprise items organized into dimensions, which usually warrant such examinations. No meta‐analytic synthesis was conducted, as only two studies on two different tools reported internal consistency statistics. Powis et al. (2021) conducted a multidimensional scaling analysis of the ERG22+ and reported the internal consistency of the extracted subscales, as well as that of the original subscales and the full tool. The Cronbach's alpha for the full scale was 0.80, and those of the original or extracted subscales varied between 0.19 and 0.85. For the IVP guidance, Cronbach's alpha was computed for the whole scale using all participants as well as subtypes of extremist groups (Egan et al. 2016). The alpha for all participants was 0.64 and varied substantially between groups (*α* = 0.32 to 0.84). It was especially low for individuals involved in animal rights groups (*α* = 0.32) or individuals who committed school shootings (*α* = 0.38). Authors generally agree that alphas below 0.70 are problematic (Tavakol and Dennick 2011). As such, the internal consistency of the IVP guidance could be considered lackluster; the same is true for many ERG22+ subscales. However, we have to keep in mind that risk tools, by design, do not operate like psychometric scales (Helmus and Babchishin 2017). Because risk tools try to minimize the redundancy of and variance shared by their items, they should not be held to the same internal consistency standards as personality scales such as the NEO‐PI (Costa and McCrae 2008).

#### 4.5.2 Validity

##### 4.5.2.1 Content

In the context of our systematic review, content validity studies most often corresponded to case studies (*n* = 1) of known terrorists on which a risk tool was retrospectively applied to assess for item fit and item relevance. Three such studies were available on the TRAP‐18: Collins and Clark (2021), Dmitrieva and Meloy (2022), and Erlandsson and Reid Meloy (2018). All three studies found that the TRAP‐18 was fit for purpose, as most of its items (distal and proximal behaviors) were endorsed by individuals who either committed or tried to commit a terrorist attack. Specifically, 14 of the 18 TRAP‐18 items were endorsed in an Incel case study (Collins and Clark 2021), 12 of 18 in a far‐right case (Dmitrieva and Meloy 2022), and 15 of 18 in a case involving a mass school shooting perpetrator with far‐right views and severe mental health issues (Erlandsson and Reid Meloy 2018).

Other types of content validity studies were available on the TRAP‐18. In Meloy et al. (2015), the content validity of the scale was tested in a sample of 22 individuals who committed terrorist acts motivated by various types of extremist ideas. According to the authors, the TRAP‐18 demonstrated good fit in real‐world data, with more than half of participants scoring positively on 13 out of 18 items (72%) on the scale.

Another avenue to study the content validity of violent extremism risk tools was employed by Kupper and Meloy (2021), who verified if the written or spoken manifestos of lone actors who planned or committed attacks contained words or sentences that could enable the scoring of TRAP‐18 items. The authors found that 17 of the 18 items (94%) could be scored positively or negatively according to the content of the manifestos. On average, manifestos comprised 4.5 of the possible 8 proximal warning behaviors and 3.8 of the possible 10 distal characteristics.

Brugh et al. (2023) also verified the extent to which TRAP‐18 items could be scored using publicly available information alone. The percentage of missing values varied by item (range = 3.9%–87%), and around half of them (11/18) were more often missing than scored. On average, half (9/18) of the TRAP‐18 items were unscored per case. Distal characteristics were deemed easier to score than proximal warning behaviors. When comparing the proportion of present and absent items while discarding unscored ones, more items were present rather than absent, leading the authors to think that, on average, TRAP‐18 items are relevant to lone‐actor terrorists. However, they concluded that triangulation of publicly available data was insufficient to score the TRAP‐18, which casts doubt on most TRAP‐18 validation studies, as all but one were scored with such data.

Finally, Meloy et al. (2021) tested if the division of TRAP‐18 items into distal and proximal characteristics was confirmed by a time sequence analysis relying on the open‐source data of 125 lone‐actor terrorists who mounted attacks in Europe or North America. The authors found that when looking at the trajectories of these individuals, most distal characteristics preceded the proximal warning behaviors that culminated in an attack. Therefore, the division of TRAP‐18 items into what equates to static/stable and acute risk factors (e.g., Douglas and Skeem 2005; Hanson and Harris 2000) was validated by that study.

One content validity study on the VERA was conducted by Beardsley and Beech (2013), who examined whether items from the scale would be found in individuals who committed terrorist attacks. Using information from Google on five highly mediatized cases, the authors concluded that the VERA was fit for purpose, as items representing multiple risk dimensions, particularly extremist attitudes, were present, while those from the protective dimension were relatively absent. Specifically, on average across the five cases, 85% of attitudinal items were present, followed by 66% of contextual items, 63% of demographic items, 40% of historical items, and only 27% of protective factor items. The authors furthermore noted that VERA items applied to both lone actors and members of offline extremist groups.

A similar approach was used by Böckler et al. (2017) to evaluate *Der Screener—Islamismus*. The authors applied the 13‐item tool to 31 anonymized case summaries of individuals convicted of jihadist activity, including completed attacks, attempted plots, and participation in terrorist organizations. On average, the tool identified nearly seven present risk indicators per case (*M* = 6.84, SD = 2.67), representing 53% of the total items. All 31 cases presented at least two flagged items. In addition, the authors conducted a face validity assessment through a workshop involving 20 frontline practitioners from education, justice, and police sectors. Participants rated the tool highly on a 5‐point Likert scale (1 = very satisfied, 5 = very dissatisfied), with overall satisfaction averaging 1.6 (SD = 0.60), perceived clarity at 1.3 (SD = 0.47), and practical utility at 1.35 (SD = 0.75), further supporting the content relevance and operational usefulness of the tool.

Evidently, by nature of being mostly curtailed to descriptive statistics, content validity studies are not well suited to test null versus alternative hypotheses and cannot answer the question of whether higher scores on risk tools lead to more negative outcomes compared to individuals with lower scores. In addition, content validity analyses were often based on a very low number of individuals—in some studies, one. Nevertheless, authors evaluating risk tools in the PVE space found that these tools demonstrated good content validity.

##### 4.5.2.2 Construct

Two studies explored the construct validity of PVE risk tools: one for the ERG22+ (Powis et al. 2021) and one for the TRAP‐18 (Goodwill and Meloy 2019). Based on a sample of individuals adhering to Islamist extremist ideas, Powis et al. (2021) conducted a principal component analysis and multidimensional scaling analysis of ERG22+ items, which both suggested the presence of multiple dimensions in the scale. Principal component analysis suggested the presence of seven dimensions accounting for 64% of the explained variance, while multidimensional scaling analysis suggested the presence of five. For both analyses, the choice of parameters was sound and well‐documented. However, neither the principal component analysis nor the multidimensional scaling analysis led to a factor structure that mirrored that of the conceptual division of the scale. Furthermore, the fit indices for the multidimensional scaling solution fell slightly short of the predefined threshold, with a coefficient of alienation (CoA) of 0.23—just above the acceptable cut‐off of 0.20 specified in the methods section. Thus, construct validity was not established for the ERG22+.

In Goodwill and Meloy (2019), multidimensional scaling was used to verify if TRAP‐18 items under the distal characteristics dimension would cluster together and separately from items under the proximal warning behaviors dimension. The analysis was documented appropriately, and the fit of the solution was deemed good (Stress‐1 = 0.098, S‐Stress = 1.69%). Proximal warning behaviors mostly clustered together, but not distal characteristics, which sometimes clustered with proximal warning behaviors and sometimes did not cluster at all. Therefore, construct validity as it relates to the division between distal and proximal behaviors was partly established. It was, however, validated in a time‐sequence analysis of TRAP‐18 indicators (Meloy et al. 2021).

##### 4.5.2.3 Convergent

Two studies—those in Hart et al. (2017)—assessed the convergent validity of violent extremism risk tools. In the first study, the authors tested the convergence between the MLG‐V2 and, respectively, the Historical Clinical Risk Management‐20 Version 3 (HCR‐20V3; Douglas et al. 2013) and the VERA. Convergent validity between the total scores of the MLG‐V2 and the HCR‐20V3 was not supported (*r* = −0.45). However, the MLG‐V2 “individual” subscale showed moderate correlations with several components of the HCR‐20V3: *r* = 0.37 with the total score, 0.55 with historical items, 0.16 with clinical items, and 0.22 with risk management items. Due to the small sample size (*n* = 5; same sample as Beardsley and Beech 2013), none of these correlations reached significance. Group‐related dimensions of the MLG‐V2 correlated negatively with HCR‐20V3 scores (range: *r* = −0.10–−0.68), a lack of covariation that the authors anticipated given that the HCR‐20V3 focuses exclusively on individual‐level factors and does not account for group influences.

With respect to the VERA, a substantial correlation was observed between its total score and that of the MLG‐V2 (*r* = 0.69). Domain‐level scores between the two tools also showed considerable overlap, with correlations ranging from −0.74 to 0.92. The strongest associations were found between the “contextual” subscale of the VERA and the group‐related dimensions of the MLG‐V2: *r* = 0.90 with “individual‐in‐group,” *r* = 0.90 with “group,” and *r* = 0.92 with “group‐in‐society.” These three correlations were the only ones to reach statistical significance in the analysis.

In the second study, Hart et al. (2017) asked three researchers to evaluate the degree of item overlap between the MLG‐V2 and VERA 2. Most of the VERA 2 items overlapped with those of the MLG‐V2, but the opposite was not true. Only two of the four MLG‐V2 dimensions—“individual” and “individual‐in‐group”—showed substantial overlap with VERA‐2 content. In contrast, the “group” and “group‐in‐society” dimensions of the MLG‐V2 were largely unrepresented in the VERA‐2.

In sum, available studies do not attest to the convergent validity of the MLG‐V2. This is in part due to the low sample size of Hart et al. (2017), making the evidence base relatively anecdotal, but also to the fact that the MLG‐V2 is a tool that integrates risk factors about the individual and their group. With most other PVE risk tools being purely focused on the individual, convergent validity is curtailed. Tools that are more similar in nature (e.g., the ERG22+ and the TRAP‐18) may be found to correlate more strongly in the future, but as of now, studies on the topic were not found in our review.

##### 4.5.2.4 Discriminant

Discriminant validity analyses conducted for violent extremism risk tools manifested in whether scores on such tools could discriminate between groups based on (a) type of violent extremist ideology, (b) type of attack, (c) country/continent, and (d) individuals working alone versus in an autonomous cell. Studies comparing whether radicalized individuals would go on to commit violence or not were classified under predictive/postdictive validity.

As to the type of ideology, Egan et al. (2016) found that IVP guidance scores were significantly lower for animal rights activists (*M* = 11.8, SD = 2.9) and school shooters (*M* = 14.9, SD = 5.3) than for Irish Republicans (*M* = 23.2, SD = 9.5), Islamists (*M* = 21.4, SD = 7.4), and right‐wing extremists (*M* = 24.7, SD = 6.4), who did not differ among themselves (*F*[4, 173] = 15.5, *p* < 0.001). Combined with the low internal consistency of the IVP guidance for animal rights activists and school shooters, the authors concluded that the scale may not be fit for purpose for these two groups. Meloy and Gill (2016) investigated whether TRAP‐18 scores significantly differed across right‐wing, Islamic, and single‐issue extremists. They found that even though some items (e.g., personal grievance and moral outrage) were more frequently found in proponents of certain extremist ideologies, average TRAP‐18 scores did not differ between the three groups (average scores ranged between 9.5 and 9.9).

Concerning the type of attack, Egan et al. (2016) tested whether the IVP guidance was more sensitive in identifying a particular violent outcome compared to others (causing injury, killing, and bombing). AUC greatly varied depending on the group under study and outcome, with the authors reaching no substantial conclusion other than the scale being potentially less sensitive, no matter the outcome, for animal rights activists and school shooters (AUC between 0.49 and 0.67).

With regard to geographic differences, Brugh et al. (2023) found that the TRAP‐18 may be less well suited for assessing lone‐actor cases from Europe compared to those from the United States. European cases had significantly more missing items (*M* = 10.4, SD = 3.5) than U.S. cases (*M* = 7.3, SD = 2.6) (*t*[70] = 2.56, *p* = 0.013). They also had fewer positively scored items (*M* = 5.7, SD = 2.6) compared to their U.S. counterparts (*M* = 8.8, SD = 3.1) (*t*[70] = 2.13, *p* = 0.037). As a result, fewer European cases were recommended for active monitoring by the tool (*p* = 0.034, Fisher's exact test), despite all individuals having been involved in attacks and/or affiliated with terrorist groups.

Two studies evaluated whether violent extremism risk tools showed differences in assessing individuals who acted alone or in a group. Egan et al. (2016) found no significant differences in IVP guidance scores between individuals who worked alone and those who worked in a group (AUC = 0.40, *n*.*s*.). In turn, Meloy et al. (2015) found nearly no differences between the TRAP‐18 scores of lone actors and individuals who were part of an autonomous cell. Only 1 of the 18 items showed significant differences: a history of criminal violence, which was more common in autonomous cell extremists (*p* = 0.005, *φ* = 0.70, Fisher's exact test).

Discriminant validity analyses collected here do not tell us much about PVE risk tools. These analyses were mostly exploratory in nature and did not specify in advance which group should theoretically obtain lower or higher scores on the scales. In that sense, they do not constitute proper discriminant validity analyses and provide very little information on the nomological network of constructs evaluated in violent extremism risk tools. They nevertheless suggest that some tools, such as the IVP guidance, might not appropriately capture the risk dynamics of some groups (animal rights activists and school shooters) and that there might not be major differences in item fit for lone‐actor terrorism and those who are members of a group. Therefore, even though scales such as the TRAP‐18 were developed specifically for lone‐actor extremists, the future may reveal that the scale could be applicable to violent extremists who are part of a group.

##### 4.5.2.5 Predictive/Postdictive

Predictive validity is often considered the litmus test for risk tool validation (Helmus and Babchishin 2017). However, as of now, only the TRAP‐18 seems to benefit from such studies, and these have substantial methodological flaws, namely, reliance on open‐source data and use of retrospective (postdictive) rather than prospective designs. In total, six postdictive validity studies were identified, and none provided evidence of true predictive validity.

Two studies evaluated whether scores on TRAP‐18 items were significantly different for radicalized individuals who did or did not go on to commit violence. Meloy and Gill (2016) compared individuals whose attacks were thwarted to individuals whose attack was not stopped. Not only did this conflate intention to act with outside elements such as police investigation, but results indicated that among the five items that were significantly different in prevalence, two were in the opposite direction (higher in the thwarted‐attack group). In Meloy et al. (2019), attackers were compared to non‐attackers. In that study, seven items were significantly more frequent in attackers, and two were more frequent in non‐attackers. Both studies did not disclose if total TRAP‐18 scores differed between the two groups. Although the TRAP‐18 is a SPJ tool and does not include a formal total score, SPJ tools can nonetheless be evaluated using the number of indicators present (see de Vogel et al. [2004] and Hanson et al. [2009] for examples). This omission limits the extent to which Meloy and Gill (2016) and Meloy et al. (2019) can be interpreted as providing postdictive validity evidence in the conventional psychometric sense.

**Figure 2:**
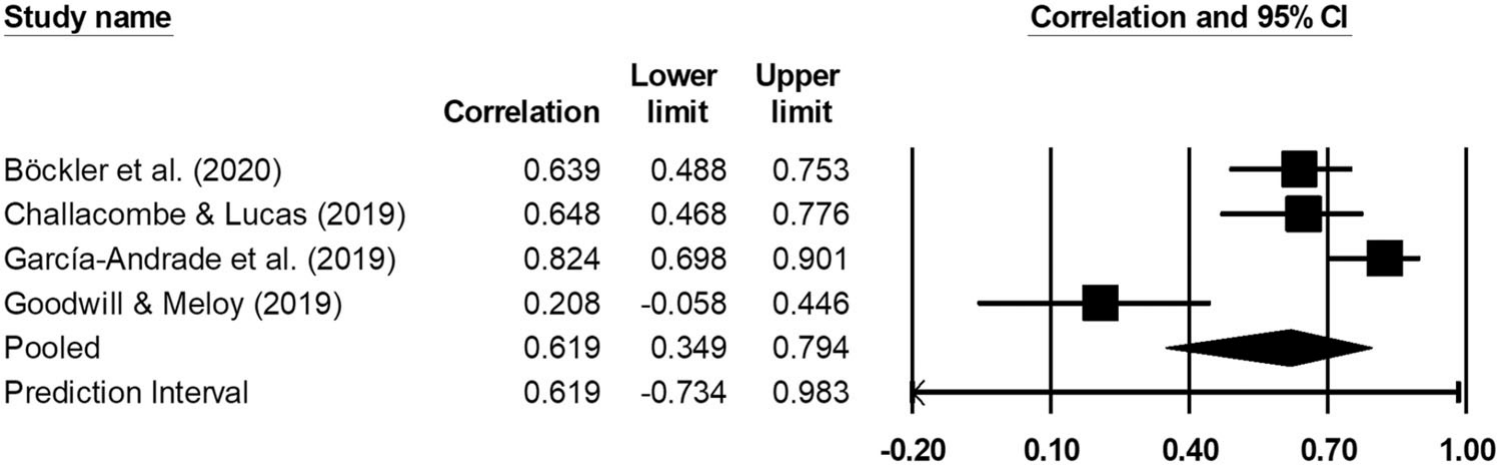
Forest plot for the meta‐analysis of the postdictive validity of the TRAP‐18.

Four studies tested whether TRAP‐18 total scores postdicted violence among samples of radicalized individuals at risk of committing extremist violence (Böckler et al. 2015; Challacombe and Lucas 2019; Fernández García‐Andrade et al. 2019; Goodwill and Meloy 2019). Their reported effect sizes can be found in Table 5. Among the four studies, one reported a perfect effect size (AUC of 1.00; Fernández García‐Andrade et al. 2019). This implies that in that study, the two participants who committed extremist violence had higher TRAP‐18 scores than all the other 42 participants in the study. To enable inclusion in the meta‐analysis, we applied a continuity correction, adjusting the AUC to 0.98. This adjusted value was then converted to a point‐biserial correlation of *r* = 0.82 following Rice and Harris's (2005) guidelines. Once all effect sizes were converted to point‐biserial correlations, a meta‐analysis of the TRAP‐18's postdictive validity was conducted, as shown in Table 6 and Figure 2.

**Table 5:** Summary of the evidence concerning the postdictive validity of the TRAP‐18.

Study	*N*	Effect sizes and conversion to point‐biserial correlation		
		AUC	Cohen's *d*	Point‐biserial *r*
Böckler et al. (2020)	80	0.880	—	0.639
Challacombe and Lucas (2019)	58	—	1.700	0.648
Fernández García‐Andrade et al. (2019)	44	1.000/0.980^a^	—	1.000/0.824^a^
Goodwill and Meloy (2019)	56	—	0.426^b^	0.208

^a^ Value after applying the continuity correction.

^b^ The Cohen's *d* was manually computed using the mean scores and standard errors of attackers versus non‐attackers.

**Table 6:** Meta‐analysis of the postdictive validity of the TRAP‐18 (random‐effect model).

	*N*	*r* [95% CI]	*Z*	*p*	*Q*	*I* ^2^	*τ*²
Study							
Böckler et al. (2020)	80	0.639 [0.488–0.753]	6.638	0.000	—	—	—
Challacombe and Lucas (2019)	58	0.648 [0.468–0.776]	5.724	0.000	—	—	—
Fernández García‐Andrade et al. (2019)	44	0.824 [0.698–0.901]	7.486	0.000	—	—	—
Goodwill and Meloy (2019)	56	0.208 [−0.058–0.446]	1.537	0.124	—	—	—
Studies pooled	238	0.619 [0.349–0.794]	3.953	0.000	21.211 (*df* = 3, *p* = 0.000)	86%	*Z* = 0.115 (*r* ≈ 0.107)
Sensitivity analysis							
Böckler et al. (2020) removed	158	0.613 [0.178–0.848]	2.620	0.009	21.927 (*df* = 2, *p* = 0.000)	91%	*Z* = 0.202 (*r* ≈ 0.199)
Challacombe and Lucas (2019) removed	180	0.610 [0.200–0.838]	2.748	0.006	21.907 (*df* = 2, *p* = 0.001)	91%	*Z* = 0.181 (*r* ≈ 0.179)
Fernández García‐Andrade et al. (2019) removed	194	0.526 [0.261–0.732]	3.281	0.001	11.522 (*df* = 2, *p* = 0.003)	83%	*Z* = 0.078 (*r* ≈ 0.078)
Goodwill and Meloy (2019) removed	182	0.708 [0.564–0.810]	7.090	0.000	5.171 (*df* = 2, *p* = 0.075)	61%	*Z* = 0.028 (*r* ≈ 0.028)

The postdictive validity of the TRAP‐18 for extremist violence varied considerably across studies, with individual effect sizes ranging from *r* = 0.208 to 0.824. The pooled correlation across the four included studies was large (*r* = 0.619, 95% CI = 0.349–0.794) and statistically significant. However, the heterogeneity was substantial (*I*² = 86%), suggesting that the variation in observed effect sizes likely reflects true differences between studies rather than sampling error alone. Sensitivity analyses revealed that the overall estimate was unstable: removing the weakest study (Goodwill and Meloy 2019) resulted in a markedly stronger pooled effect (*r* = 0.708, *p* < 0.001), whereas removing any of the other studies reduced the correlation and, in some cases, its statistical significance. These findings point to the promise of the TRAP‐18 as a potential tool to distinguish between individuals who do and do not engage in extremist violence—but they also raise significant concerns about its consistency across samples.

To contextualize these findings, it is helpful to compare the TRAP‐18 to established risk assessment tools in adjacent fields. For example, the Static‐99R—a widely used instrument for assessing sexual recidivism risk—has demonstrated a pooled AUC of 0.69 (*r* ≈ 0.38) in a large‐scale meta‐analysis (Helmus et al. 2022). Similarly, the Violence Risk Appraisal Guide (VRAG; Harris et al. 1993) has shown an AUC of 0.74 (*r* ≈ 0.48), and the HCR‐20, commonly used to assess violence risk in psychiatric settings, has reached an AUC of 0.70 (*r* ≈ 0.40) according to Singh et al. (2011). In the criminological field, the LS/CMI—a widely used general recidivism risk tool—showed an average correlation of approximately 0.30 in a meta‐analysis by Olver et al. (2014). Against this backdrop, the TRAP‐18's pooled correlation of *r* = 0.62 appears unusually high. While this may reflect the tool's potential to discriminate between individuals who did and did not engage in extremist violence, it also raises concerns about possible sampling bias or model overfitting, particularly given the retrospective nature and modest sample sizes of the included studies. Moreover, no study to date has tested the TRAP‐18's predictive validity using prospective methods, which are known to result in more conservative estimates compared to retrospective designs (Hanson et al. 2009). As such, the current evidence base, though encouraging, remains preliminary and should be interpreted with appropriate caution.

## 5 Discussion

### 5.1 Summary of Main Results

The objectives of the current systematic review were to synthesize evidence about the psychometric validation of risk tools available in the PVE space. Encouraging results were found concerning the inter‐rater agreement of scales in research contexts, but one of the two studies that examined it in a routine/field setting obtained disappointing results. Content validity studies were mostly positive, indicating that PVE risk tools adequately cover the risk factors and offending processes of individuals who go on to commit extremist violence. Construct validity analyses were few and far between, with results indicating that empirical divisions of scales did not match their conceptual divisions. The internal consistency of subscales was lackluster, while that of full scales was acceptable. Only one study examined convergent validity, and it revealed a lack of convergence, primarily due to particularities of the scale under study (the MLG‐V2). Discriminant validity analyses were exploratory in nature rather than true tests of null versus alternative hypotheses, but suggested that most PVE risk tools might not be ideology‐specific and may apply to both lone and group actors. Finally, even though encouraging results were found regarding the predictive validity of scales—arguably the most important validation criterion—effect sizes varied substantially and were based on research designs that cannot truly test predictive validity, hence the use of the term “postdictive validity” by the authors. Such data was only available on the TRAP‐18.

### 5.2 Overall Completeness and Applicability of Evidence

The 20 studies on PVE risk tool validation included in this review focused on the following tools: the TRAP‐18, ERG22+, MLG‐V2, VERA, IVP guidance, and *Der Screener—Islamismus*. Apart from the TRAP‐18, no risk tool had more than two validation studies comprising primary data. Furthermore, many risk tools beyond those did not benefit from any publicly available validation study. The Extremism Monitoring Instrument (EMI‐20; Schmid 2014), IAT8, Islamic Radicalization (IR‐46; Elzinga et al. 2010), Radar (Barrelle 2015), RADAR‐iTE (Sadowski et al. 2021), Radicalization Risk Assessment in Prisons (RRAP; Esgalhado et al. 2018), *Référentiel des indicateurs de basculement dans la radicalization* (Comité interministériel de prévention de la délinquance 2016), and Vulnerability Assessment Framework (HM Government 2012) were all tools that were included in our search strategy, but for which no eligible validation study was found. The lack of psychometric validation data for many of these tools was also reported by Clesle et al. (2025) and Lloyd (2019).

This suggests that some widely used and publicized violent extremism risk tools rely on evidence that is either nonexistent, very slim, or not published by governments and organizations. The issue of data not being made available to researchers has been known for a long time in the PVE space (Ayres 2021; Sageman 2014) and continues to be a hindrance to the confidence that practitioners can have in violent extremism risk tools. In comparison, research on criminological risk scales dates back to the start of the 20th century, with Burgess' (1928) seminal paper on the predictors of parole success. Meta‐analyses on criminal recidivism risk scales commonly find dozens of eligible papers (e.g., 128 for the LS/CMI [Olver et al. 2014] and 56 for the Static‐99R [Helmus et al. 2022]). While data on perpetrators of extremist violence is sensitive, so is data on adjudicated individuals and perpetrators of sexual violence, which has nevertheless been used for more than 50 years to anchor best practice guidelines in the evaluation of the risk, case management, and treatment of individuals involved in criminal careers.

Although several postdictive validity studies reported AUC values, which offer insight into a tool's overall performance, they provide only limited information about false positives and false negatives—a critical concern in low base rate contexts like PVE. Notably, only one study (Egan et al. 2016) reported sensitivity and specificity alongside AUC. However, this was not a postdictive design but rather a discriminant validity analysis comparing subtypes of attackers. As a result, we lack direct evidence on one of the most pressing concerns in the application of PVE risk tools: their potential to generate high false positive rates when applied in operational settings.

Another factor related to the completeness of the evidence base is that samples comprised nearly exclusively male participants. Some samples did comprise women, but these represented 2% to 5% of the total sample (Böckler et al. 2020; Egan et al. 2016; Goodwill and Meloy 2019; Meloy et al. 2015, 2019, 2021). One exception was present in Challacombe and Lucas (2019), where women constituted 14% of the sample. While this underrepresentation possibly reflects the lower involvement of women in violent extremist acts compared to men, it nonetheless limits the ability to draw gender‐specific conclusions. As such, it is unlikely that current risk assessment research in the PVE field enables a meaningful understanding of the dynamics of risk in women.

Finally, none of the studies examined the predictive or postdictive validity of summary risk judgments (e.g., low/moderate/high) produced by practitioners using SPJ tools—judgments that differ from the additive scoring approaches used in current validation studies. This lack of predictive or postdictive validation for structured summary judgments represents a notable blind spot in the validation of PVE risk tools. It is particularly striking given that nearly all such tools follow a SPJ model. If developers of these instruments wish to empirically demonstrate the added value of SPJ frameworks over actuarial approaches, they should test whether professional judgments made after tool administration predict violent extremist outcomes—or whether they improve upon predictions based on summed item scores alone. Designs of this nature are well established in the broader field of violence risk assessment (Campbell et al. 2009; Singh et al. 2011) and could be readily adapted for PVE risk tool validation. In the absence of such evidence, it remains difficult to ascertain whether these tools meaningfully support decision‐making in operational or clinical contexts, underscoring the limited ecological validity of currently available validation studies.

### 5.3 Quality of the Evidence

Risk of bias was omnipresent in risk tool validation studies and included multiple forms of reporting bias, as defined by Campbell Collaboration guidelines (Aloe et al. 2024). First, all studies but one on the TRAP‐18 and all studies on the MLG‐V2, IVP guidance, and VERA were based on publicly available data. This means that for these studies, information on participants resulted from public terrorist databases, newspaper articles, manifestos, Google searches, and autobiographies. While open‐source data has notable strengths—such as accessibility, transparency, and relevance for understanding how radicalized individuals present in public discourse—it also comes with well‐documented limitations. Scholars have emphasized that such data may be biased, influenced by government agendas, limited by the effectiveness of journalistic investigations, or, in the case of manifestos and autobiographies, susceptible to social desirability bias (Ayres 2021; Sageman 2014; Spaaij and Hamm 2015). Additionally, as emphasized by Brugh et al. (2023), using open‐source data entails a lot of missing information, which makes tools such as the TRAP‐18 impractical to score.

Importantly, in the context of violent extremism risk tool validation, the reliance on open‐source data often constrains how outcomes are chosen, which may compromise their relevance for actual risk prediction and contribute to selective outcome reporting. For example, in Egan et al. (2016), because the database did not comprise non‐attackers, the AUCs reported did not discriminate attackers from non‐attackers but rather distinguished between different types of attackers. The reported AUCs compared different types of attackers (e.g., bombers vs. shooters), a comparison that, while potentially interesting for descriptive profiling, cannot establish predictive/postdictive validity. In turn, Meloy and Gill (2016) compared individuals whose attacks were thwarted to those who completed them—an outcome that conflates intent to act with factors such as planning, conscientiousness, and the effectiveness of the police investigation. Some postdictive validity studies, however, did compare radicalized individuals who acted out to those who did not (Böckler et al. 2015; Challacombe and Lucas 2019; Fernández García‐Andrade et al. 2019; Goodwill and Meloy 2019).

The second main source of bias was the reliance on retrospective rather than prospective designs. With predictive validity being the litmus test of risk tool validation, and with true predictive validity being only testable via prospective designs (Mathes and Pieper 2019), it was surprising that not a single prospective study was found. The lack of prospective designs was inextricably linked to the use of publicly available data, which cannot be, by nature, prospective. Using retrospective data runs the risk of mixing up temporal sequences and paves the way for reverse causation and recall biases. Hindsight or outcome bias (Henriksen and Kaplan 2003) is also a concern: researchers retrospectively scoring risk tools based on known case outcomes (e.g., a completed attack) may unintentionally assign higher risk scores to those individuals, inflating apparent postdictive validity. That said, the absence of prospective designs in the included studies must also be interpreted within the broader context of PVE research. The low base rate of violent extremist recidivism, ethical constraints around prediction in non‐correctional populations, and restricted access to institutional samples make prospective designs exceptionally difficult to implement (Borum 2015; Hodwitz 2021; Sarma 2017; Silke and Morrison 2020). As such, while the lack of prospective research weakens the overall certainty of evidence, it may reflect practical limitations rather than methodological neglect.

Third, very small samples were a threat to validity. Five studies relied on samples of five or fewer individuals preselected by authors (Beardsley and Beech 2013; Collins and Clark 2021; Dmitrieva and Meloy 2022; Erlandsson and Reid Meloy 2018; Hart et al. 2017). They were mostly descriptive, did not enable a test of the null hypothesis, and were likely limited in terms of external validity.

The trifecta of using small sample sizes, retrospective open‐source data, and convenience outcomes—along with the associated biases—represents a real threat to generalizability. According to our initial guidelines, no reviewed studies could be rated as methodologically robust. Three were rated as average (Fernández García‐Andrade et al. 2019; Powis et al. 2019, 2021), as they did not rely on open‐source data, and the rest (*k* = 17) were rated as below average. It is not a given that the findings gathered in the current systematic review, particularly in terms of predictive/postdictive validity, will be replicated once more methodologically rigorous designs are employed. Interestingly, these flaws did not manifest in COSMIN scores, which seemed to assess mostly for sample size considerations and the appropriateness of the analytical strategy. Studies that obtained lower COSMIN scores were mostly case studies or studies with very few participants.

In addition to these structural limitations, author affiliation, study funding, and potential conflicts of interest represent a final source of reporting bias. Although such involvement is common in early‐stage validation research and often reflects specialized expertise with the instrument, it also raises concerns about potential inflation of predictive/postdictive validity estimates. Fazel et al. (2022) found that studies conducted by tool developers tend to report more favorable predictive performance than independent evaluations. For instance, the TRAP‐18 is a commercially available instrument marketed by Multi‐Health Systems, and its lead developer, who is an author on most validation studies, may receive royalties. While such arrangements are not uncommon in psychological assessment, they underscore the need for independent replication to reduce the risk of bias and enhance confidence in the generalizability of findings. We do not imply any ill intent or misconduct—on the contrary, most authors in this review were transparent about their methodological constraints. However, in accordance with Campbell Collaboration guidelines, this potential conflict of interest must be acknowledged. Independent replications remain essential to corroborate the utility and validity of these tools—and we are confident that such studies will emerge in due course.

Another core limitation of the current evidence base is its lack of ecological validity. Nearly all included studies relied on open‐source data or clinical vignettes, rather than real‐world assessments conducted by practitioners. These designs often involve limited and/or missing data, which may underrepresent key psychosocial and contextual factors relevant to risk. Moreover, they do not reflect the informational environments in which practitioners operate—environments that vary substantially depending on the intervention setting (RTI International 2018). In secondary prevention contexts, for instance, practitioners may lack access to police or institutional records (and may deliberately avoid relying on them), but are typically better positioned to assess psychological functioning, motivation, and protective factors through direct engagement. In tertiary or correctional settings, by contrast, access to institutional and criminal history data is generally more robust. Yet to date, no prospective validation studies of PVE risk tools have captured this variation—unlike what has been done in the broader criminological risk assessment literature (e.g., Hanson et al. 2007). In short, while it remains unclear how more ecologically valid conditions will impact estimates of reliability and validity, it is evident that current research does not reflect real‐world assessment contexts—posing a key limitation for interpreting the practical utility of these tools.

Despite the substantial threats to validity and potential reporting biases, it is paramount to put things in perspective: Imperfect data is preferable to no data—especially if it is presented as such. In most of the reviewed papers, authors were very cognizant of the limitations of the evidence and made conclusions accordingly. There were very few limitations that we noted that the authors had not already mentioned. As such, we did not feel like authors and/or risk tool developers were minimizing data quality issues while presenting their tool as the gold standard. This also manifested in the quality of analytical strategies, which generally upheld standards in risk tool psychometric testing and validation (Brouillette‐Alarie et al. 2016; Hanson 2021; Helmus and Babchishin 2017). Flaws were mostly present in research designs and sample constitutions. Researchers and developers of PVE risk tools can only do so much with the data they are provided and authorized to access. It is easy for systematic review authors to report that methodological designs are not strong enough; it is much harder for researchers, stakeholders, and practitioners to assemble the conditions to make better research happen. In that, we acknowledge the numerous hurdles that shift researchers towards open‐source data.

### 5.4 Potential Biases in the Review Process

The current systematic review is not without limitations. First, the December 31, 2021, end date for inclusion means that some papers that would otherwise have been eligible were not included in the review. We have documented some of these papers, which are listed in Table 7 (note that this list is not intended to be exhaustive). Although we did not integrate them into our analyses, we did go through each of these papers and can conclude that their results do not impact our main conclusion: To date, there is no prospective study attesting to the predictive validity of any violent extremism risk tool. Some of these papers are, however, methodologically sophisticated and would compare favorably, in terms of sample constitution and methods, to those included in this systematic review. These papers also provide new evidence on the VERA‐2R, which was until recently quite slim.

**Table 7:** Recently published papers on violent extremism risk tools.

Study	Tools	Data source	Sample	Reliability	Validity
Challacombe and Patrick (2023)	TRAP‐18	Publicly available data	*N* = 101 (Capitol insurrectionists)	Interrater	Postdictive
Cherney and Belton (2024)	VERA‐2R	Publicly available data	*N* = 50 (various ideologies)	Interrater	Content, postdictive
Corner and Pyszora (2022)	TRAP‐18	Focus groups + interviews	*N* = 58 (PVE experts)	N/A	Content
Corner and Taylor (2023)	Radar, VERA‐2R	Publicly available data + official records	*N* = 30 (university students, PVE experts, trained assessors) using the tools on 60 anonymized vignettes	Interrater	Content, discriminant,^a^ postdictive
Duits and Kempes (2023)	VERA‐2R	Official records	*N* = 2 raters (researchers) using the tool on 30 extremist cases	Interrater	N/A
Elliott et al. (2023)	ERG22+	Official records	*N* = 310 (various ideologies but mostly Islamist VE)	Internal consistency	Construct, content
Tassin and Allely (2022)	TRAP‐18	Publicly available data	*N* = 1 (Islamist VE)	N/A	Content

^a^ The authors refer to the equity of instruments, that is, whether they result in higher scores for some ideologies compared to others.

Second, because of unreleased PVE risk tool evaluations, we may only have a truncated picture of the reliability, validity, and usefulness of such tools. Systematic reviews are limited by the accessibility of scientific evidence, and evidence kept under wraps will necessarily have evaded the scrutiny of the current review.

Third, another limitation may result from the variability introduced by each rater. We attempted to address this by measuring and monitoring inter‐rater agreement rates, updating training as required, and reaching consensus when raters had divergent ratings or made different selections. However, inter‐rater reliability remained in the moderate range, suggesting room for improvement.

Finally, because of the limited methodological quality of PVE risk tool validation research, we were not able to complete objective 4, which aimed to provide tool recommendations for specific audiences and contexts. The current state of evidence precludes the recommendation of any tool over another—much less specific ones for specific settings. Indeed, the heterogeneity of validation techniques and outcomes precluded us from running moderator analyses, as too few studies were available on the same tools to enable meta‐regression data synthesis. It is noteworthy, however, that tools were much more ideology‐agnostic than anticipated. Apart from *Der Screener—Islamismus*, surveyed tools were designed to apply to radicalized individuals no matter their ideology, and discriminant validity (or equity) evaluations suggested these tools did not provide arbitrarily higher scores for specific ideological affiliations (Corner and Taylor 2023; Meloy and Gill 2016).

## 6 Authors' Conclusions

### 6.1 Implications for Research

The findings of this review, despite the aforementioned limitations, allow us to offer informed recommendations for future work. With regard to research, first and foremost, the issue of data being withheld by governments and organizations is well‐documented in the field (Cubitt and Wolbers 2022; RTI International 2018). Some tools rely on validation data only present in internal reports (e.g., the IR‐46; Lloyd 2019). In other cases, data that exists is neither made available to researchers for independent evaluation nor published by government‐affiliated researchers in publicly available organization reports or peer‐reviewed scientific journals. For example, many governments and security agencies are known to use the VERA‐2R to structure surveillance, case management, and resource allocation in both pre‐ and post‐crime contexts (Duits et al. 2023). However, no studies have yet been published demonstrating whether these screenings are predictive of future extremist violence or intentions to act, despite the fact that operational use implies that relevant data may exist. Two recent postdictive studies (Cherney and Belton 2024; Corner and Taylor 2023) signal growing interest in addressing this gap, but predictive evidence remains elusive. In parallel, existing ERG22+ data could feasibly support predictive validity analyses if the criminal records of assessed individuals were collected to identify subsequent extremism‐related offenses (or their absence).

However, low base rates would likely be an issue for such studies. While base rates of recidivism for violent extremism (approximately 3%; Hodwitz 2021; Silke and Morrison 2020) are not dramatically lower than those observed in other fields like sexual violence prevention (5%–10%; Lussier et al. 2023), the key issue is the substantially smaller sample sizes typically available in violent extremism research. As a result, the absolute number of individuals who reoffend is often so low that group comparisons or predictive analyses become severely underpowered, regardless of the statistical technique employed. Although effect size measures such as AUC are relatively robust to low base rates (Fawcett 2006; Swets 1988), they still require a sufficient number of positive outcome cases to yield stable and interpretable estimates. This constraint highlights the need for outcome measures that extend beyond terrorism‐related recidivism. Future PVE risk tool validation studies could consider multidimensional outcomes that encompass extremist attitudes, intentions, or lower‐level behaviors. Such outcomes are conceptually relevant and would likely occur at a higher frequency, increasing the number of analyzable cases and improving statistical power. For example, tertiary prevention evaluations have often used proxy measures of re‐radicalization due to the frequent unavailability of recidivism data (Hassan et al. 2021; Demant et al. 2009; van der Heide and Schuurman 2018). While such alternative outcomes are not recommended for operational use, they may provide meaningful insights for research purposes.

Beyond sharing undisclosed existing data to researchers, there is also a need in the PVE space for the development and implementation of new and robust research initiatives to assess the reliability, validity, and relevance of risk assessment tools. Despite the numerous articles published since Scarcella et al.'s (2016) systematic review, the robustness of research designs has not improved substantially, which suggests that new initiatives are needed instead of reusing the same open‐source data currently in circulation. To enable new research efforts in the field, multisectoral collaboration is likely needed. Such initiatives need to be funded by decision‐makers who see value in obtaining accurate data about PVE risk tools (including changing their practices if results are not in the anticipated direction). They also need to be designed and implemented by researchers with expertise in risk tool evaluation and prospective designs. Crucially, future research initiatives need to have buy‐in from practitioners, as they will be the ones using the tools and collecting data. If practitioners are neither well‐trained nor receptive to using such tools (because of time constraints or personal advice), they are unlikely to collect reliable data on them, which will curtail both reliability and validity. Quality of training was found to be one of the most important predictive validity moderators for sexual violence scales, with higher‐quality training being consistently associated with higher predictive validity (Helmus et al. 2022). Finally, some amount of high‐level provincial and federal coordination should also be helpful to ensure that data collected in different organizations and provinces is comparable. This will allow pooling data to reach higher sample sizes, which is likely necessary for discriminative analyses, considering the low base rate of violent extremism reoffenses.

In sum, the current systematic review highlights in no uncertain terms the need for higher‐quality validation data as it pertains to PVE risk assessment tools. Non‐convenience samples and prospective predictive validity designs are urgently needed. Even though some authors have questioned the viability of such analyses (e.g., Sarma 2017), quality research from the fields of sexual violence, general criminality, and violence has proven that such analyses are possible, even with relatively low base rates (e.g., Helmus et al. 2022; Olver et al. 2014).

Finally, we do not believe that further reviews of the available PVE risk assessment literature are needed, as literature reviews on PVE risk tools have multiplied in recent years and mostly reached the same conclusions about the lack of quality validation data (Corner and Taylor 2023; Clesle et al. 2025; Cubitt and Wolbers 2022; Risk Management Authority 2021; RTI International 2018; Scarcella et al. 2016; van der Heide et al. 2019). Therefore, if one were to have to choose between funding new research initiatives and funding new literature syntheses, we would encourage the former rather than the latter—at least until the quality of validation data improves sufficiently to warrant a new investigation of the available literature.

### 6.2 Implications for Practice

The current state of validation data raises concerns regarding the legal admissibility of violent extremism risk tools in judicial proceedings. As an illustrative example, in the United States, the Frye v. United States (1923) and Daubert v. Merrell Dow Pharmaceuticals Inc. (1993) standards outline criteria to determine the admissibility of expert testimony and the tools used to support it. These include: (1) empirical testing of the tool's reliability and validity; (2) a known error rate or misclassification potential; (3) peer‐reviewed publication of supporting evidence; and (4) general acceptance within the relevant scientific community (Faigman et al. 2014, 2016; Helmus et al. 2022; Neal et al. 2019). While the TRAP‐18 is the only tool for which postdictive validity data were readily available, it may not meet the first two criteria due to methodological limitations and possible bias in the validation studies. This example from U.S. law highlights broader issues that would likely raise admissibility concerns in other jurisdictions as well.

In many legal systems, decisions related to liberty, supervision, or sentencing require the use of rigorously validated tools. At present, the evidence base supporting PVE risk tools may be insufficient to provide courts with confidence that these instruments consistently produce valid assessments. Moreover, consensus regarding the functionality of PVE risk tools is lacking within the field, as reflected in critical commentary from numerous scholars (e.g., Borum 2015; Monahan 2012; Sarma 2017). Although some critiques may hold these tools to idealized standards that even well‐established instruments in other fields may not meet, the absence of high‐quality evidence remains a legitimate concern—particularly when such tools inform decisions that significantly impact individuals' rights and liberties. Legal actors and policymakers should therefore exercise caution when relying on PVE risk tools in high‐stakes contexts.

The lack of predictive validity data does not mean, however, that PVE risk tools should be discarded entirely. The last 100 years of research in criminology have unequivocally demonstrated that structured assessments of risk outperform unstructured assessments (i.e., those relying on pure clinical judgment without support from any tool; Hanson 2009). In that sense, currently available risk tools constitute lists of (mostly) relevant risk and protective factors for violent extremism that can be used by practitioners to structure clinical reflections, assess risk, and plan interventions. Assessing each case by considering all relevant risk and protective factors while discarding irrelevant ones has historically been the recipe for success in risk assessment (Hanson 2009).

To that end, it might be worthwhile for risk tool developers to revisit the risk and protective factors (items) present in PVE risk tools, as the scientific literature has significantly evolved since the design and release of most of the tools for which validation studies were documented (Corner and Taylor 2023). Many authors critical of the viability of risk assessment in the field rightly mentioned the lack of scientific literature on risk and protective factors for violent extremism at the time risk tools were developed (Borum 2015; Monahan 2012; Sarma 2017). Now that, years later, the first meta‐analyses and systematic reviews have come out (Desmarais et al. 2017; Emmelkamp et al. 2020; Gill et al. 2021; Lösel et al. 2018; Misiak et al. 2019; Vergani et al. 2020; Wolfowicz et al. 2020), it may be time to review existing tools with an updated evidence base. Regarding potential updates, many authors have mentioned that ideological and political factors may have been overemphasized compared to risk factors for crime and violence (Wolfowicz et al. 2020). The relative weight of these dimensions could thus be revisited in currently used assessment scales.

### 6.3 Agreements and Disagreements With Other Studies and Reviews

The conclusions of the present Campbell review are broadly consistent with those of two previous systematic reviews on violent extremism risk assessment tools (Clesle et al. 2025; Scarcella et al. 2016), despite important differences in scope, methods, inclusion criteria, and publication timing. For reference, of the 20 studies included in our review, three were also covered in Scarcella et al. (2016), and 10 in Clesle et al. (2025). Although most of the validation studies in this field were published within the last 8–9 years, Scarcella et al. had already concluded in 2016 that “based on the quality reporting and on the psychometric properties (or the lack thereof), there is no substantial evidence that would enable the authors to recommend one instrument over another” (p. 17). Nearly a decade later, our conclusion remains, unfortunately, quite similar.

All three systematic reviews converge on a central finding: the evidence base concerning the psychometric properties of violent extremism risk tools remains insufficient to support confident, empirically informed guidelines for tool selection and deployment. Although a growing number of such tools are being used by frontline practitioners, data on their validity remains sparse (Clesle et al. 2025). Even the TRAP‐18—supported by what appears to be the most active research community—still lacks essential validation, particularly in the form of prospective predictive validity analyses. Some methodologically rigorous studies have begun to emerge for the ERG22+ and VERA‐2R, occasionally using official records instead of publicly available data; however, robust predictive or postdictive validity analyses are still missing. Moreover, recent work has raised concerns about the psychometric properties of the VERA‐2R (Cherney and Belton 2024; Corner and Taylor 2023).

That all three systematic reviews—despite differences in inclusion criteria, methodology, and intended audiences—arrive at similarly cautious conclusions underscores the persistent challenges of conducting rigorous validation research in this field. Collectively, these reviews portray a maturing field still in search of a solid empirical footing, and underscore the urgent need for better‐designed, prospective, and transparent studies to support sound clinical and operational decision‐making in PVE. Encouragingly, the increasing volume and methodological sophistication of recent studies suggest that the field is moving in the right direction, laying the groundwork for a stronger and more evidence‐informed future.

## Author Contributions

*Sébastien Brouillette‐Alarie and Ghayda Hassan*: co‐leads of the systematic review who were involved at all stages of the process (conceptualization of the review, production of the search strategy, coordination of the search, selection, and data extraction phases, analysis of the data, and redaction of the manuscript).

*Wynnpaul Varela, Emmanuel Danis, and Deniz Kilinc*: research assistants who were involved in the selection of articles, data extraction, and analysis of the data. Wynnpaul Varela also acted as our English writing expert. Deniz Kilinc completed his studies in 2020 and, thus, was mostly involved in the early stages of the review.

*Sarah Ousman, Pablo Madriaza, and Eugene Borokhovski*: co‐authors involved in the conceptualization of the review and manuscript writing. Sarah Ousman substantially helped to develop and test the search strategy.

*Inga L. Pauls and Robert Pelzer*: responsible for coordinating and conducting the German literature search, as well as selecting and coding these articles. They also contributed to drafting the manuscript.

*David Pickup*: library science expert who reviewed the search syntax, conducted the official literature searches, indexed the results, and produced documents for the article selection phase.

## Conflicts of Interest

The authors declare no conflicts of interest.

## Plans for Updating the Review

At present, there are no plans to update this review, as the allocated funding has been fully expended. However, should the evidence base on violent extremism risk assessment tools expand significantly and warrant a new synthesis, the lead researchers would likely seek funding to support an update. All data extraction and coding files have been securely archived and can support future updating efforts if resources become available.

## Differences Between Protocol and Review

Deviations from the initial protocol include conducting a literature search specifically in German (requested by Public Safety Canada; see the Bibliographic Search Phases section) and the inability to conduct more advanced meta‐analytic techniques (e.g., meta‐regression) due to the limited number of studies available for quantitative data synthesis (see the Data Synthesis section). Additionally, the Australian Criminology Database (CINCH, Informit), which had been listed in the protocol, was not searched due to a miscommunication between our team and the Campbell editorial group. This omission has been noted, and the database will be included in any future update of the review, should one be undertaken.

Note that the presentation of the methodology slightly diverges from the protocol to improve readability and clarity, especially in the Search Methods for Identification of Studies section. However, this has no practical implications for the way the search was conducted.

## Sources of Support

This project was funded by the Community Resilience Fund of Public Safety Canada, in collaboration with the Crime and Justice Group of the Campbell Collaboration. The views expressed herein do not necessarily reflect those of Public Safety Canada or the Campbell Collaboration.

## Supporting information

Appendices.

## Data Availability

The data that support the findings of this study are available from the corresponding author upon reasonable request.

## Endnotes

1 The risk‐need‐responsivity (RNR) model is a widely used framework in correctional psychology. It recommends that (1) the intensity of interventions should match the person's risk of reoffending (*risk*), (2) services should target criminogenic needs—factors that contribute to offending (*need*), and (3) services should be delivered in a way that is responsive to the person's learning style, abilities, and strengths (*responsivity*; Bonta and Andrews 2024).

2 Actuarial tools estimate risk by mechanically combining empirically validated predictors, with items selected for their statistical—rather than theoretical—association with outcomes. Each item is pre‐weighted, coding is highly structured, and professional discretion is minimal. The total score (sum of items) is then compared to normative tables to determine the individual's risk level (Brouillette‐Alarie and Lussier 2018).

## References

[cl270080-bib-0227] Bartlett, J. , and C. Miller . 2012. “The Edge of Violence: Towards Telling the Difference Between Violent and Non‐Violent Radicalization.” Terrorism and Political Violence 24, no. 1: 1–21. 10.1080/09546553.2011.594923.

[cl270080-bib-0001] Beardsley, N. L. , and A. R. Beech . 2013. “Applying the Violent Extremist Risk Assessment (VERA) to a Sample of Terrorist Case Studies.” Journal of Aggression, Conflict and Peace Research 5, no. 1: 4–15. 10.1108/17596591311290713.

[cl270080-bib-0003] Böckler, N. , M. Allwinn , C. Metwaly , B. Wypych , J. Hoffmann , and A. Zick . 2020. “Islamist Terrorists in Germany and Their Warning Behaviors: A Comparative Assessment of Attackers and Other Convicts Using the TRAP‐18.” Journal of Threat Assessment and Management 7, no. 3–4: 157–172. 10.1037/tam0000150.

[cl270080-bib-0002] Böckler, N. , M. Allwinn , J. Hoffmann , and A. Zick . 2017. “Früherkennung von islamistisch motivierter Radikalisierung. Vorstellung und empirische Validierung eines verhaltensbasierten Instrumentes zum Fallscreening [Early Detection of Islamist‐Motivated Radicalization: Presentation and Empirical Validation of a Behavior‐Based Instrument for Case Screening].” Kriminalistik 8–9, no. 2017: 491–497. https://pub.uni-bielefeld.de/record/2913614.

[cl270080-bib-0004] Brugh, C. S. , S. L. Desmarais , and J. Simons‐Rudolph . 2023. “Application of the TRAP‐18 Framework to U.S. and Western European Lone Actor Terrorists.” Studies in Conflict & Terrorism 46, no. 2: 183–208. 10.1080/1057610X.2020.1758372.

[cl270080-bib-0005] Challacombe, D. J. , and P. A. Lucas . 2019. “Postdicting Violence With Sovereign Citizen Actors: An Exploratory Test of the TRAP‐18.” Journal of Threat Assessment and Management 6, no. 1: 51–59. 10.1037/tam0000105.

[cl270080-bib-0006] Collins, C. J. , and J. J. Clark . 2021. “Using the TRAP‐18 to Identify an Incel Lone‐Actor Terrorist.” Journal of Threat Assessment and Management 8, no. 4: 159–173. 10.1037/tam0000167.

[cl270080-bib-0228] de Vogel, V. , C. de Ruiter , D. van Beek , and G. Mead . 2004. “Predictive Validity of the SVR‐20 and Static‐99 in a Dutch Sample of Treated Sex Offenders.” Law and Human Behavior 28, no. 3: 235–251. 10.1023/B:LAHU.0000029137.41974.eb. 15264445

[cl270080-bib-0007] Dmitrieva, A. M. , and J. R. Meloy . 2022. “Troubled Waters: Domestic Terrorism Threat in the U.S. Coast Guard and the TRAP‐18.” Journal of Threat Assessment and Management 9, no. 3: 153–170. 10.1037/tam0000170.

[cl270080-bib-0008] Egan, V. , J. Cole , B. Cole , et al. 2016. “Can You Identify Violent Extremists Using a Screening Checklist and Open‐Source Intelligence Alone?.” Journal of Threat Assessment and Management 3, no. 1: 21–36. 10.1037/tam0000058.

[cl270080-bib-0009] Erlandsson, Å. , and J. Reid Meloy . 2018. “The Swedish School Attack in Trollhättan.” Journal of Forensic Sciences 63, no. 6: 1917–1927. 10.1111/1556-4029.13800. 29684937

[cl270080-bib-0010] Fernández García‐Andrade, R. , B. Serván Rendón‐Luna , B. Reneses Prieto , V. Vidal Martínez , E. Medina Téllez de Meneses , and E. Fernández Rodríguez . 2019. “Forensic‐Psychiatric Assessment of the Risk of Terrorist Radicalisation in the Mentally Ill Patient.” Spanish Journal of Legal Medicine 45, no. 2: 59–66. 10.1016/j.remle.2019.01.003.

[cl270080-bib-0011] Goodwill, A. , and J. R. Meloy . 2019. “Visualizing the Relationship Among Indicators for Lone Actor Terrorist Attacks: Multidimensional Scaling and the TRAP‐18.” Behavioral Sciences & the Law 37, no. 5: 522–539. 10.1002/bsl.2434. 31758736

[cl270080-bib-0012] Hart, S. D. , A. N. Cook , D. E. Pressman , S. Strang , and Y. L. Lim . 2017. *A Concurrent Evaluation of Threat Assessment Tools for the Individual Assessment of Terrorism* (Report No. 17‐1). Canadian Network for Research on Terrorism, Security, and Society (TSAS). https://www.tsas.ca/publications/a-concurrent-evaluation-of-threat-assessment-tools-for-the-individual-assessment-of-terrorism/.

[cl270080-bib-0013] Kupper, J. , and J. R. Meloy . 2021. “TRAP‐18 Indicators Validated Through the Forensic Linguistic Analysis of Targeted Violence Manifestos.” Journal of Threat Assessment and Management 8, no. 4: 174–199. 10.1037/tam0000165.

[cl270080-bib-0015] Meloy, J. R. , A. Goodwill , C. Clemmow , and P. Gill . 2021. “Time Sequencing the TRAP‐18 Indicators.” Journal of Threat Assessment and Management 8, no. 1–2: 1–19. 10.1037/tam0000157.

[cl270080-bib-0016] Meloy, J. R. , A. M. Goodwill , M. J. Meloy , G. Amat , M. Martinez , and M. Morgan . 2019. “Some TRAP‐18 Indicators Discriminate Between Terrorist Attackers and Other Subjects of National Security Concern.” Journal of Threat Assessment and Management 6, no. 2: 93–110. 10.1037/tam0000119.

[cl270080-bib-0014] Meloy, J. R. , and P. Gill . 2016. “The Lone‐Actor Terrorist and the TRAP‐18.” Journal of Threat Assessment and Management 3, no. 1: 37–52. 10.1037/tam0000061.

[cl270080-bib-0017] Meloy, J. R. , K. Roshdi , J. Glaz‐Ocik , and J. Hoffmann . 2015. “Investigating the Individual Terrorist in Europe.” Journal of Threat Assessment and Management 2, no. 3–4: 140–152. 10.1037/tam0000036.

[cl270080-bib-0019] Powis, B. , K. Randhawa‐Horne , I. Elliot , and J. Woodhams . 2019. Inter‐Rater Reliability of the Extremism Risk Guidelines 22+ (ERG 22+). Ministry of Justice. https://www.gov.uk/government/publications/inter-rater-reliability-of-the-extremism-risk-guidelines-22-erg-22.

[cl270080-bib-0018] Powis, B. , K. Randhawa , and D. Bishopp . 2021. “An Examination of the Structural Properties of the Extremism Risk Guidelines (ERG22+): A Structured Formulation Tool for Extremist Offenders.” Terrorism and Political Violence 33, no. 6: 1141–1159. 10.1080/09546553.2019.1598392.

[cl270080-bib-0020] Aguerri, J. C. , and C. Fernández Abad . 2021. “La orden de servicios 3/2018: ¿un instrumento para medir el riesgo de radicalismo violento en prisión?.” Estudios Penales Y Criminológicos 41: 361–413. 10.15304/epc.41.6724.

[cl270080-bib-0021] Ahearn, E. R. , K. Bhui , and E. Jones . 2021. “What Factors Are Truly Associated With Risk for Radicalisation? A Secondary Data Analysis Within a UK Sample.” Transcultural Psychiatry 58, no. 5: 645–653. 10.1177/1363461520933755. 32611222

[cl270080-bib-0022] Al‐Farajat, A. M. , and H. Al‐sharah . 2018. “The Ability of Maladaptive Schemas in Prediction of Intellectual Extremism.” Arab Journal of Psychiatry 29, no. 1: 67–81. 10.12816/0046446.

[cl270080-bib-0023] Allely, C. S. , and L. Faccini . 2019. “Clinical Profile, Risk, and Critical Factors and the Application of the “Path Toward Intended Violence” Model in the Case of Mass Shooter Dylann Roof.” Deviant Behavior 40, no. 6: 672–689. 10.1080/01639625.2018.1437653.

[cl270080-bib-0024] Altemeyer, B. , and B. Hunsberger . 2004. “Research: A Revised Religious Fundamentalism Scale: The Short and Sweet of It.” International Journal for the Psychology of Religion 14, no. 1: 47–54. 10.1207/s15327582ijpr1401_4.

[cl270080-bib-0025] Bélanger, J. J. , M. Moyano , H. Muhammad , et al. 2019. “Radicalization Leading to Violence: A Test of the 3N Model.” Frontiers in Psychiatry 10: 42. 10.3389/fpsyt.2019.00042. 30853917 PMC6396731

[cl270080-bib-0026] Besta, T. , and M. Błażek . 2007. “Polska adaptacja Skali Fundamentalizmu Religijnego autorstwa B. Altemeyera i B. Hunsbergera [Polish Adaptation of Religious Fundamentalism Scale by B. Altemeyer and B. Hunsberger].” Przegląd Psychologiczny 50, no. 4: 347–365.

[cl270080-bib-0027] Bhui, K. , M. Otis , K. Halvorsrud , M. Freestone , and E. Jones . 2020. “Assessing Risks of Violent Extremism in Depressive Disorders: Developing and Validating a New Measure of Sympathies for Violent Protest and Terrorism.” Australian & New Zealand Journal of Psychiatry 54, no. 11: 1078–1085. 10.1177/0004867420944520. 32702996

[cl270080-bib-0028] Bhui, K. , M. Otis , M. J. Silva , K. Halvorsrud , M. Freestone , and E. Jones . 2020. “Extremism and Common Mental Illness: Cross‐Sectional Community Survey of White British and Pakistani Men and Women Living in England.” British Journal of Psychiatry 217, no. 4: 547–554. 10.1192/bjp.2019.14. PMC7525107 30873926

[cl270080-bib-0029] Bhui, K. , N. Warfa , and E. Jones . 2014. “Is Violent Radicalisation Associated With Poverty, Migration, Poor Self‐Reported Health and Common Mental Disorders?.” PLoS One 9, no. 3: e90718. 10.1371/journal.pone.0090718. 24599058 PMC3944722

[cl270080-bib-0030] Böckler, N. , J. Hoffmann , and A. Zick . 2015. “The Frankfurt Airport Attack: A Case Study on the Radicalization of a Lone‐Actor Terrorist.” Journal of Threat Assessment and Management 2, no. 3–4: 153–163. 10.1037/tam0000045.

[cl270080-bib-0031] Bootsma, L. , and E. Harbers . 2021. “Assessing Potentially Violent Extremists: Experiences From Dutch Investigative Psychologists.” In International Handbook of Threat Assessment (2nd ed.), edited by J. R. Meloy and J. Hoffmann , 639–653. Oxford University Press. 10.1093/med-psych/9780190940164.003.0035.

[cl270080-bib-0032] Brayton, K. J. 2004. “The Measurement of Bias and Risk Assessment in Perpetrators of Bias Motivated Acts of Violence (Publication No. 3162759).” Doctoral diss., Palo Alto University. ProQuest Dissertations & Theses. https://www.proquest.com/dissertations-theses/measurement-bias-risk-assessment-perpetrators/docview/305076706/se-2.

[cl270080-bib-0033] Carlucci, L. , M. Tommasi , M. Balsamo , A. Furnham , and A. Saggino . 2015. “Religious Fundamentalism and Psychological Well‐Being: An Italian Study.” Journal of Psychology and Theology 43, no. 1: 23–33.

[cl270080-bib-0034] Carlucci, L. , M. Tommasi , and A. Saggino . 2013. “Factor Structure of the Italian Version of the Religious Fundamentalism Scale.” Psychological Reports 112, no. 1: 6–13. 10.2466/07.17.PR0.112.1.6-13. 23654022

[cl270080-bib-0035] Clemmow, C. 2020. “Risk Factors and Indicators for Engagement in Violent Extremism.” Doctoral diss., University College London. UCL Discovery. https://discovery.ucl.ac.uk/id/eprint/10116345/.

[cl270080-bib-0036] Clemmow, C. , P. Gill , N. Bouhana , J. Silver , and J. Horgan . 2020. “Disaggregating Lone‐Actor Grievance‐Fuelled Violence: Comparing Lone‐Actor Terrorists and Mass Murderers.” Terrorism and Political Violence 34, no. 3: 558–584. 10.1080/09546553.2020.1718661.

[cl270080-bib-0037] Conley, C. 2019. “*Risk Assessment Tools Developed for Radicalized Individuals and Their Application in a Correctional Context* (Research Report R‐425).” Correctional Service of Canada. https://www.csc-scc.gc.ca/research/r-425-en.shtml.

[cl270080-bib-0038] Cowan, R. G. , and R. Cole . 2022. “The Pathway to Violence and Public Mass Shooters in Mental Health Treatment Before Attacks.” Safer Communities 21, no. 1: 31–44. 10.1108/sc-05-2021-0020.

[cl270080-bib-0039] Cunningham, M. D. 2018. “Differentiating Delusional Disorder From the Radicalization of Extreme Beliefs: A 17‐Factor Model.” Journal of Threat Assessment and Management 5, no. 3: 137–154. 10.1037/tam0000106.

[cl270080-bib-0040] da Silva, R. , P. Fernández‐Navarro , M. M. Gonçalves , C. Rosa , and J. Silva . 2019. “Tracking Narrative Change in the Context of Extremism and Terrorism: Adapting the Innovative Moments Coding System.” Aggression and Violent Behavior 47: 204–214. 10.1016/j.avb.2019.05.002.

[cl270080-bib-0041] Dean, G. , and G. Pettet . 2017. “The 3R's of Risk Assessment for Violent Extremism.” Journal of Forensic Practice 19, no. 2: 91–101. 10.1108/JFP-07-2016-0029.

[cl270080-bib-0042] Dehlin, A. J. , and R. V. Galliher . 2019. “Young Women's Sexist Beliefs and Internalized Misogyny: Links With Psychosocial and Relational Functioning and Political Behavior.” Psi Chi Journal of Psychological Research 24, no. 4: 255–246. 10.24839/2325-7342.JN24.4.255.

[cl270080-bib-0043] Denovan, A. , N. Dagnall , K. Drinkwater , A. Parker , and P. Clough . 2017. “Perception of Risk and Terrorism‐Related Behavior Change: Dual Influences of Probabilistic Reasoning and Reality Testing.” Frontiers in Psychology 8: 1721. 10.3389/fpsyg.2017.01721. 29062288 PMC5633603

[cl270080-bib-0044] Dover, H. , M. Miner , and M. Dowson . 2007. “The Nature and Structure of Muslim Religious Reflection.” Journal of Muslim Mental Health 2, no. 2: 189–210. 10.1080/15564900701614858.

[cl270080-bib-0045] Dunbar, E. , J. Quinones , and D. A. Crevecoeur . 2005. “Assessment of Hate Crime Offenders: The Role of Bias Intent in Examining Violence Risk.” Journal of Forensic Psychology Practice 5, no. 1: 1–19. 10.1300/J158v05n01_01.

[cl270080-bib-0046] Eisenman, D. P. , and L. Flavahan . 2017. “Canaries in the Coal Mine: Interpersonal Violence, Gang Violence, and Violent Extremism Through a Public Health Prevention Lens.” International Review of Psychiatry 29, no. 4: 341–349. 10.1080/09540261.2017.1343527. 28805121

[cl270080-bib-0047] Fiedler, N. , F. Sommer , V. Leuschner , and H. Scheithauer . 2019. “Student Crisis Prevention in Schools: The NETWorks Against School Shootings Program (NETWASS)—An Approach Suitable for the Prevention of Violent Extremism?.” International Journal of Developmental Science 13, no. 3–4: 109–122. 10.3233/DEV-190283.

[cl270080-bib-0048] Furnham, A. , G. Horne , and S. Grover . 2020. “Correlates of the Militant Extremist Mindset.” Frontiers in Psychology 11: 2250. 10.3389/fpsyg.2020.02250. 32982896 PMC7492641

[cl270080-bib-0049] Gordon, T. J. , Y. Sharan , and E. Florescu . 2017. “Potential Measures for the Pre‐Detection of Terrorism.” Technological Forecasting and Social Change 123: 1–16. 10.1016/j.techfore.2017.05.017.

[cl270080-bib-0050] Gottschalk, M. , and S. Gottschalk . 2004. “Authoritarianism and Pathological Hatred: A Social Psychological Profile of the Middle Eastern Terrorist.” American Sociologist 35, no. 2: 38–59. 10.1007/BF02692396.

[cl270080-bib-0051] Grossman, M. , K. Hadfield , P. Jefferies , V. Gerrand , and M. Ungar . 2020. “Youth Resilience to Violent Extremism: Development and Validation of the BRAVE Measure.” Terrorism and Political Violence 34, no. 3: 468–488. 10.1080/09546553.2019.1705283.

[cl270080-bib-0052] Grossman, M. , M. Ungar , J. Brisson , V. Gerrand , K. Hadfield , and P. Jefferies . 2017. Understanding Youth Resilience to Violent Extremism: A Standardised Research Measure. Alfred Deakin Institute for Citizenship and Globalisation. 10.13140/RG.2.2.21022.79689.

[cl270080-bib-0053] Guldimann, A. , and J. R. Meloy . 2020. “Assessing the Threat of Lone‐Actor Terrorism: The Reliability and Validity of the TRAP‐18.” Forensische Psychiatrie, Psychologie, Kriminologie 14, no. 2: 158–166. 10.1007/s11757-020-00596-y.

[cl270080-bib-0054] Hammer, J. H. , and A. Lazar . 2019. “Internal Structure and Criterion Relationships for Long and Brief Versions of the Intratextual Fundamentalism Scale (IFS) Among Israeli Jews.” Psychology of Religion and Spirituality 11, no. 4: 358–367. 10.1037/rel0000148.

[cl270080-bib-0055] Harder, Q. N. 2018. “Qualitative Case Study Analysis of Domestic Terrorist Data for Use in Community‐Based Counternarrative Program Development (Publication No. 10980087).” Doctoral diss., Northcentral University. ProQuest Dissertations & Theses. https://www.proquest.com/dissertations-theses/qualitative-case-study-analysis-domestic/docview/2139717332/se-2.

[cl270080-bib-0056] Haroun, A. 2003. “Psychiatric Evaluation of Suspected Terrorists.” Psychiatric Annals 33, no. 11: 738–742. 10.3928/0048-5713-20031101-10.

[cl270080-bib-0057] Herzog‐Evans, M. 2018. “A Comparison of Two Structured Professional Judgment Tools for Violent Extremism and Their Relevance in the French Context.” European Journal of Probation 10, no. 1: 3–27. 10.1177/2066220317749140.

[cl270080-bib-0058] Ho, C. S. H. , T. C. Quek , R. C. M. Ho , and C. C. Choo . 2019. “Terrorism and Mental Illness: A Pragmatic Approach for the Clinician.” BJPsych Advances 25, no. 2: 101–109. 10.1192/bja.2018.49.

[cl270080-bib-0059] Hung, B. W. K. 2017. “A Graph‐Based, Systems Approach for Detecting Violent Extremist Radicalization Trajectories and Other Latent Behaviors.” Doctoral diss., Colorado State University. 10.25675/3.022986.

[cl270080-bib-0060] Hung, B. W. K. , A. P. Jayasumana , and V. W. Bandara . 2018. “INSiGHT: A System to Detect Violent Extremist Radicalization Trajectories in Dynamic Graphs.” Data & Knowledge Engineering 118: 52–70. 10.1016/j.datak.2018.09.003.

[cl270080-bib-0061] Hunsberger, B. 1996. “Religious Fundamentalism, Right‐Wing Authoritarianism, and Hostility Toward Homosexuals in Non‐Christian Religious Groups.” International Journal for the Psychology of Religion 6, no. 1: 39–49. 10.1207/s15327582ijpr0601_5.

[cl270080-bib-0062] Janjua, Z. 2021. “What Predicts the Militant Extremist Mindset? An Investigation Into the Relationship Between Violent Extremism, and Personality, Moral Disengagement, and Linguistic Markers.” Doctoral diss., University of Nottingham (Nottingham Theses). https://eprints.nottingham.ac.uk/62093/.

[cl270080-bib-0063] Kenyon, J. 2020. “Exploring the Role of the Internet in the Radicalisation Process and Offending of Individuals Convicted of Extremist Offences.” Doctoral diss., Nottingham Trent University. https://irep.ntu.ac.uk/id/eprint/44237.

[cl270080-bib-0064] Kerodal, A. G. , J. D. Freilich , and S. M. Chermak . 2016. “Commitment to Extremist Ideology: Using Factor Analysis to Move Beyond Binary Measures of Extremism.” Studies in Conflict & Terrorism 39, no. 7–8: 687–711. 10.1080/1057610X.2016.1141012.

[cl270080-bib-0065] Klausen, J. , S. Campion , N. Needle , G. Nguyen , and R. Libretti . 2016. “Toward a Behavioral Model of “Homegrown” Radicalization Trajectories.” Studies in Conflict & Terrorism 39, no. 1: 67–83. 10.1080/1057610X.2015.1099995.

[cl270080-bib-0066] Klausen, J. , R. Libretti , B. W. K. Hung , and A. P. Jayasumana . 2018. “Radicalization Trajectories: An Evidence‐Based Computational Approach to Dynamic Risk Assessment of “Homegrown” Jihadists.” Studies in Conflict & Terrorism 43, no. 7: 588–615. 10.1080/1057610X.2018.1492819.

[cl270080-bib-0067] Knudsen, R. A. 2020. “Measuring Radicalisation: Risk Assessment Conceptualisations and Practice in England and Wales.” Behavioral Sciences of Terrorism and Political Aggression 12, no. 1: 37–54. 10.1080/19434472.2018.1509105.

[cl270080-bib-0068] Lemieux, F. , and J. Regens . 2012. “Assessing Terrorist Risks: Developing an Algorithm‐Based Model for Law Enforcement.” Pakistan Journal of Criminology 3, no. 3: 33–49.

[cl270080-bib-0069] Liht, J. , L. G. Conway, III , S. Savage , W. White , and K. A. O'Neill . 2011. “Religious Fundamentalism: An Empirically Derived Construct and Measurement Scale.” Archive for the Psychology of Religion 33, no. 3: 299–323. 10.1163/157361211X594159.

[cl270080-bib-0070] Lloyd, M. 2021. “Making Sense of Terrorist Violence and Building Psychological Expertise.” In International Handbook of Threat Assessment (2nd ed.), edited by J. R. Meloy and J. Hoffmann , 624–638. Oxford University Press. 10.1093/med-psych/9780190940164.003.0034.

[cl270080-bib-0071] Lloyd, M. , and C. Dean . 2015. “The Development of Structured Guidelines for Assessing Risk in Extremist Offenders.” Journal of Threat Assessment and Management 2, no. 1: 40–52. 10.1037/tam0000035.

[cl270080-bib-0072] Logan, C. , and R. Sellers . 2020. “Risk Assessment and Management in Violent Extremism: A Primer for Mental Health Practitioners.” Journal of Forensic Psychiatry & Psychology 32, no. 3: 355–377. 10.1080/14789949.2020.1859591.

[cl270080-bib-0073] Lone, Jr., R. F. 2002. “Right‐Wing Authoritarianism and Religious Fundamentalism as Related to Universal‐Diverse Orientation.” Doctoral diss., Oklahoma State University, Open Research Oklahoma. https://hdl.handle.net/20.500.14446/336150.

[cl270080-bib-0074] Loza, W. 2010. “The Prevalence of Middle Eastern Extremist Ideologies Among Some Canadian Offenders.” Journal of Interpersonal Violence 25, no. 5: 919–928. 10.1177/0886260509336966. 19584405

[cl270080-bib-0075] Loza, W. , Y. Abd‐el‐Fatah , J. Prinsloo , A. Hesselink‐Louw , and K. Seidler . 2011. “The Prevalence of Extreme Middle Eastern Ideologies Around the World.” Journal of Interpersonal Violence 26, no. 3: 522–538. 10.1177/0886260510363417. 20448230

[cl270080-bib-0076] Manganelli Rattazzi, A. M. , A. Bobbio , and L. Canova . 2007. “A Short Version of the Right‐Wing Authoritarianism (RWA) Scale.” Personality and Individual Differences 43, no. 5: 1223–1234. 10.1016/j.paid.2007.03.013.

[cl270080-bib-0077] Meloy, J. R. 2018. “The Operational Development and Empirical Testing of the Terrorist Radicalization Assessment Protocol (TRAP‐18).” Journal of Personality Assessment 100, no. 5: 483–492. 10.1080/00223891.2018.1481077. 29927673

[cl270080-bib-0078] Meloy, J. R. , and J. Genzman . 2016. “The Clinical Threat Assessment of the Lone‐Actor Terrorist.” Psychiatric Clinics of North America 39, no. 4: 649–662. 10.1016/j.psc.2016.07.004. 27836158

[cl270080-bib-0079] Meloy, J. R. , and Hoffmann, J. , eds. 2021. International Handbook of Threat Assessment (2nd ed.). Oxford University Press. 10.1093/med-psych/9780190940164.001.0001.

[cl270080-bib-0080] Meyer, R. A. 1976. “Multivariate Analyses of Social and Religious Attitudes.” Doctoral diss., Louisiana State University. https://repository.lsu.edu/gradschool_disstheses/2746.

[cl270080-bib-0081] Morris, H. L. , and J. Nicoletti . 2018. “Kinetic Insider Violence and Mass Shootings.” In Violence Goes to College: The Authoritative Guide to Prevention, Intervention, and Response (3rd ed.), edited by C. Bollinger , R. Flintoft , J. Nicoletti , S. Spencer‐Thomas and M. Dvoskina , 316–346. Charles C. Thomas.

[cl270080-bib-0082] Moskalenko, S. , and C. McCauley . 2009. “Measuring Political Mobilization: The Distinction Between Activism and Radicalism.” Terrorism and Political Violence 21, no. 2: 239–260. 10.1080/09546550902765508.

[cl270080-bib-0083] Mourad, M. 2018. “Tailoring Violent Extremism Prevention: A Targeted Intervention Method.” Master's thesis, Naval Postgraduate School, Defense Technical Information Center. https://apps.dtic.mil/sti/citations/AD1069668.

[cl270080-bib-0084] Muluk, H. , and N. G. Sumaktoyo . 2010. “Intratextual Fundamentalism and the Desire for Simple Cognitive Structure: The Moderating Effect of the Ability to Achieve Cognitive Structure.” Archive for the Psychology of Religion 32, no. 2: 217–238. 10.1163/157361210X500919.

[cl270080-bib-0085] Pathé, M. T. , D. J. Haworth , T. J. Lowry , et al. 2015. “A Model for Managing the Mentally Ill Fixated Person at Major Events.” Australian & New Zealand Journal of Psychiatry 49, no. 7: 610–615. 10.1177/0004867415581022. 25859053

[cl270080-bib-0086] Pendley, J. A. 2018. “The Cloudy Crystal Ball: Detecting and Disrupting Homegrown Violent Extremism.” Master's thesis, Naval Postgraduate School, Defense Technical Information Center. https://apps.dtic.mil/sti/citations/AD1052799.

[cl270080-bib-0087] Petrov, V. E. , A. V. Kokurin , V. I. Ekimova , A. V. Koteneva , and T. N. Berezina . 2019. “The Assessment of Tolerance of Military Personnel to Extremist Ideology.” Psychology and Law 9, no. 2: 69–83. 10.17759/psylaw.2019090205.

[cl270080-bib-0088] Pressman, D. E. 2009. “Risk Assessment Decisions for Violent Political Extremism.” *Public Safety Canada*. https://www.publicsafety.gc.ca/cnt/rsrcs/pblctns/2009-02-rdv/index-en.aspx.

[cl270080-bib-0089] Pressman, D. E. 2016. “The Complex Dynamic Causality of Violent Extremism: Applications of the VERA‐2 Risk Assessment Method to CVE Initiatives.” In Disaster Forensics: Understanding Root Cause and Complex Causality, edited by A. J. Masys , 249–269. Springer International Publishing. 10.1007/978-3-319-41849-0_10.

[cl270080-bib-0090] Pressman, D. E. , and J. Flockton . 2012. “Calibrating Risk for Violent Political Extremists and Terrorists: The VERA 2 Structured Assessment.” British Journal of Forensic Practice 14, no. 4: 237–251. 10.1108/14636641211283057.

[cl270080-bib-0091] Pressman, D. E. , and C. Ivan . 2019. “Internet Use and Violent Extremism: A Cyber‐VERA Risk Assessment Protocol.” In Multigenerational Online Behavior and Media Use: Concepts, Methodologies, Tools, and Applications, edited by M. Khosrow‐Pour , 266–284. Information Science Reference/IGI Global. 10.4018/978-1-5225-7909-0.ch015.

[cl270080-bib-0092] Richards, J. 2018. “High Risk or Low Risk: Screening for Violent Extremists in DDR Programmes.” International Peacekeeping 25, no. 3: 373–393. 10.1080/13533312.2018.1440177.

[cl270080-bib-0093] Shaffer, B. A. , and B. M. Hastings . 2007. “Authoritarianism and Religious Identification: Response to Threats on Religious Beliefs.” Mental Health, Religion & Culture 10, no. 2: 151–158. 10.1080/13694670500469949.

[cl270080-bib-0094] Shrestha, A. , L. Kaati , and K. Cohen . 2020. “Extreme Adopters in Digital Communities.” Journal of Threat Assessment and Management 7, no. 1–2: 72–84. 10.1037/tam0000143.

[cl270080-bib-0095] Stankov, L. 2018. “Psychological Processes Common to Social Conservatism and Terrorism.” Personality and Individual Differences 120: 75–80. 10.1016/j.paid.2017.08.029.

[cl270080-bib-0096] Stankov, L. , D. Higgins , G. Saucier , and G. Knežević . 2010. “Contemporary Militant Extremism: A Linguistic Approach to Scale Development.” Psychological Assessment 22, no. 2: 246–258. 10.1037/a0017372. 20528052

[cl270080-bib-0097] Stankov, L. , G. Knežević , G. Saucier , B. Radović , and B. Milovanović . 2018. “Militant Extremist Mindset and the Assessment of Radicalization in the General Population.” Journal of Individual Differences 39, no. 2: 88–98. 10.1027/1614-0001/a000253.

[cl270080-bib-0098] Torregrosa, J. , and Á. Panizo . 2018. “RiskTrack: Assessing the Risk of Jihadi Radicalization on Twitter Using Linguistic Factors.” In Intelligent Data Engineering and Automated Learning – IDEAL 2018 (Vol. 11315), edited by H. Yin , D. Camacho , P. Novais and A. Tallón‐Ballesteros , 21–29. Springer. 10.1007/978-3-030-03496-2_3.

[cl270080-bib-0099] Trip, S. , M. I. Marian , A. Halmajan , M. I. Drugas , C. H. Bora , and G. Roseanu . 2019. “Irrational Beliefs and Personality Traits as Psychological Mechanisms Underlying the Adolescents' Extremist Mind‐Set.” Frontiers in Psychology 10: 1184. 10.3389/fpsyg.2019.01184. 31231270 PMC6558417

[cl270080-bib-0100] Trujillo, H. M. , M. Prados , and M. Moyano . 2016. “Psychometric Properties of the Spanish Version of the Activism and Radicalism Intention Scale.” International Journal of Social Psychology: Revista de Psicología Social 31, no. 1: 157–189. 10.1080/02134748.2015.1101317.

[cl270080-bib-0101] Unterrainer, H.‐F. , J. Ruttinger , A. J. Lewis , J. Anglim , A. Fink , and H.‐P. Kapfhammer . 2016. “Vulnerable Dark Triad Personality Facets Are Associated With Religious Fundamentalist Tendencies.” Psychopathology 49, no. 1: 47–52. 10.1159/000443901. 26953723

[cl270080-bib-0102] Van Brunt, B. , A. Murphy , and A. Zedginidze . 2017. “An Exploration of the Risk, Protective, and Mobilization Factors Related to Violent Extremism in College Populations.” Violence and Gender 4, no. 3: 81–101. 10.1089/vio.2017.0039.

[cl270080-bib-0103] Warren, J. I. , A. C. R. Leviton , J. Reed , et al. 2018. “Operationalizing Theory: A Moral‐Situational Action Model for Extremist Violence.” Journal of Threat Assessment and Management 5, no. 4: 205–226. 10.1037/tam0000118.

[cl270080-bib-0104] Warren, J. I. , A. C. R. Leviton , G. B. Saathoff , et al. 2020. “Using the Moral–Situational Action Violence Risk Model for Assessing Women Involved in Extremist Violence: An Empirical Study.” Journal of Threat Assessment and Management 7, no. 1–2: 41–71. 10.1037/tam0000148.

[cl270080-bib-0105] Weems, L. L. 1998. “Religiosity and Religious Attitudes as They Relate to Mysticism and Sexual Permissiveness (Publication No. 9901352).” Doctoral diss., University of Southern Mississippi. ProQuest Dissertations & Theses. https://www.proquest.com/dissertations-theses/religiosity-religious-attitudes-as-they-relate/docview/304452783/se-2.

[cl270080-bib-0106] Williamson, W. P. , and A. Ahmad . 2007. “Survey Research and Islamic Fundamentalism: A Question about Validity.” Journal of Muslim Mental Health 2, no. 2: 155–176. 10.1080/15564900701614809.

[cl270080-bib-0107] Williamson, W. P. , J. Bishop , and R. W. Hood, Jr. 2014. “Religious Fundamentalism and Racial Prejudice: A Comparison of Implicit and Explicit Approaches.” Mental Health, Religion & Culture 17, no. 8: 847–859. 10.1080/13674676.2014.935729.

[cl270080-bib-0108] Williamson, W. P. , R. W. Hood, Jr. , A. Ahmad , M. Sadiq , and P. C. Hill . 2010. “The Intratextual Fundamentalism Scale: Cross‐Cultural Application, Validity Evidence, and Relationship With Religious Orientation and the Big 5 Factor Markers.” Mental Health, Religion & Culture 13, no. 7–8: 721–747. 10.1080/13674670802643047.

[cl270080-bib-0109] Wong, M. Y. H. , P. V. Khiatani , and W. H. Chui . 2019. “Understanding Youth Activism and Radicalism: Chinese Values and Socialization.” Social Science Journal 56, no. 2: 255–267. 10.1016/j.soscij.2018.08.006.

[cl270080-bib-0110] Zierhoffer, D. 2014. “Threat Assessment: Do Lone Terrorists Differ From Other Lone Offenders.” Journal of Strategic Security 7, no. 3: 48–62. 10.5038/1944-0472.7.3.3.

[cl270080-bib-0111] N/A.

[cl270080-bib-0112] N/A.

[cl270080-bib-0113] Aloe, A. M. , O. Dewidar , E. A. Hennessy , et al. 2024. “Campbell Standards: Modernizing Campbell's Methodologic Expectations for Campbell Collaboration Intervention Reviews (MECCIR).” Campbell Systematic Reviews 20: e1445. 10.1002/cl2.1445. 39376895 PMC11456310

[cl270080-bib-0114] Altemeyer, B. , and B. Hunsberger . 1992. “Authoritarianism, Religious Fundamentalism, Quest, and Prejudice.” International Journal for the Psychology of Religion 2, no. 2: 113–133. 10.1207/s15327582ijpr0202_5.

[cl270080-bib-0115] Andrews, D. A. , J. Bonta , and S. J. Wormith . 2004. The Level of Service/Case Management Inventory (LS/CMI). Multi‐Health Systems.

[cl270080-bib-0116] Ayres, T. 2021. “Research on International Terrorism.” In The Encyclopedia of Research Methods in Criminology and Criminal Justice, 724–731. 10.1002/9781119111931.ch136.

[cl270080-bib-0117] Barrelle, K. 2015. “Pro‐Integration: Disengagement From and Life After Extremism.” Behavioral Sciences of Terrorism and Political Aggression 7, no. 2: 129–142. 10.1080/19434472.2014.988165.

[cl270080-bib-0118] Bonta, J. , and D. A. Andrews . 2024. The Psychology of Criminal Conduct (7th ed.). Routledge.

[cl270080-bib-0119] Borenstein, M. 2019. *Common Mistakes in Meta‐Analysis and How to Avoid Them*. Biostat.

[cl270080-bib-0120] Borenstein, M. , L. Hedges , J. Higgins , and H. Rothstein . 2022a. *Comprehensive Meta‐Analysis* (Version 4). Biostat. https://meta-analysis.com/.

[cl270080-bib-0121] Borenstein, M. , L. Hedges , J. Higgins , and H. Rothstein . 2022b. Introduction to Meta‐Analysis. Wiley.

[cl270080-bib-0122] Borum, R. 2011. “Radicalization Into Violent Extremism I: A Review of Social Science Theories.” Journal of Strategic Security 4, no. 4: 7–36. 10.5038/1944-0472.4.4.1.

[cl270080-bib-0123] Borum, R. 2015. “Assessing Risk for Terrorism Involvement.” Journal of Threat Assessment and Management 2, no. 2: 63–87. 10.1037/tam0000043.

[cl270080-bib-0124] Brouillette‐Alarie, S. , K. M. Babchishin , R. K. Hanson , and L.‐M. Helmus . 2016. “Latent Constructs of the Static‐99R and Static‐2002R: A Three‐Factor Solution.” Assessment 23, no. 1: 96–111. 10.1177/1073191114568114. 25612625

[cl270080-bib-0125] Brouillette‐Alarie, S. , G. Hassan , S. Ousman , et al. 2025. “Systematic Review on the Outcomes of Tertiary Prevention Programs in the Field of Violent Radicalization.” Journal for Deradicalization 42, no. Spring 2025: 140–193. https://journals.sfu.ca/jd/index.php/jd/article/view/1027.

[cl270080-bib-0126] Brouillette‐Alarie, S. , G. Hassan , W. Varela , et al. 2022. “Systematic Review on the Outcomes of Primary and Secondary Prevention Programs in the Field of Violent Radicalization.” Journal for Deradicalization 30, no. Spring 2022: 117–168. https://journals.sfu.ca/jd/index.php/jd/article/view/577.

[cl270080-bib-0127] Brouillette‐Alarie, S. , and P. Lussier . 2018. “The Risk Assessment of Offenders With a History of Sexual Crime: Past, Present and New Perspectives.” In Sexual Offending: A Criminological Perspective, edited by P. Lussier and E. Beauregard , 349–375. Routledge.

[cl270080-bib-0128] Burgess, E. 1928. “Factors Determining Success or Failure in Parole.” In The Workings of the Indeterminate‐Sentence Law and the Parole System in Illinois, edited by A. Bruce , A. Harno , E. Burgess and J. Landesco , 205–249. State Board of Parole.

[cl270080-bib-0129] Campbell, M. A. , S. French , and P. Gendreau . 2009. “The Prediction of Violence in Adult Offenders: A Meta‐Analytic Comparison of Instruments and Methods of Assessment.” Criminal Justice and Behavior 36, no. 6: 567–590. 10.1177/0093854809333610.

[cl270080-bib-0130] Challacombe, D. J. , and C. L. Patrick . 2023. “The January 6th Insurrection at the U.S. Capitol: What the TRAP‐18 Can Tell Us About the Participants.” Journal of Threat Assessment and Management 10, no. 3: 220–228. 10.1037/tam0000194.

[cl270080-bib-0131] Cherney, A. , and E. Belton . 2024. *Testing the Reliability and Validity of the VERA‐2R on Individuals Who Have Radicalised in Australia*. Australian Institute of Criminology. https://www.aic.gov.au/sites/default/files/2024-06/crg_40_21_22_testing_the_reliability_and_validity_of_the_vera-2r.pdf.

[cl270080-bib-0132] Clesle, A. , J. Knäble , and M. Rettenberger . 2025. “Risk and Threat Assessment Instruments for Violent Extremism: A Systematic Review.” Journal of Threat Assessment and Management 12, no. 1: 1–22. 10.1037/tam0000223.

[cl270080-bib-0133] Comité interministériel de prévention de la délinquance . 2016. Guide interministériel de prévention de la radicalisation. https://www.cipdr.gouv.fr/wp-content/uploads/2018/02/guide-interministériel-de-prevention-de-la-radicalisation-Mars-2016.pdf.

[cl270080-bib-0134] Cook, A. N. , S. D. Hart , and P. R. Kropp . 2013. *Multi‐Level Guidelines for the Assessment and Management of Group‐Based Violence*. Mental Health, Law, and Policy Institute, Simon Fraser University.

[cl270080-bib-0135] Cook, A. N. , S. D. Hart , and P. R. Kropp . 2015. *Multi‐Level Guidelines for the Assessment and Management of Group‐Based Violence* (Rev. ed.). Mental Health, Law, and Policy Institute, Simon Fraser University.

[cl270080-bib-0136] Corner, E. , and N. Pyszora . 2022. “The Terrorist Radicalization Assessment Protocol‐18 (TRAP‐18) in Australia: Face Validity, Content Validity, and Utility in the Australian Context.” Journal of Policing, Intelligence and Counter Terrorism 17, no. 3: 246–268. 10.1080/18335330.2022.2117993.

[cl270080-bib-0137] Corner, E. , and H. Taylor . 2023. *Testing the Reliability, Validity, and Equity of Terrorism Risk Assessment Instruments*. Australian National University. https://www.homeaffairs.gov.au/foi/files/2023/fa-230400097-document-released-part-1.PDF.

[cl270080-bib-0138] Costa, Jr. P. T. , and R. R. McCrae . 2008. “The Revised NEO Personality Inventory (NEO‐PI‐R).” In The SAGE Handbook of Personality Theory and Assessment, Vol. 2. Personality Measurement and Testing, edited by G. J. Boyle , G. Matthews and D. H. Saklofske , 179–198. Sage Publications Inc. 10.4135/9781849200479.n9.

[cl270080-bib-0139] Cubitt, T. , and H. Wolbers . 2022. Review of Violent Extremism Risk Assessment Tools in Division 104 Control Orders and Division 105A Post‐Sentence Orders. Australian Institute of Criminology. https://www.aic.gov.au/sites/default/files/2023-05/sr14.pdf.

[cl270080-bib-0140] Daubert v. Merrell Dow Pharmaceuticals Inc . 1993. 509 U.S. 579, 113 S. Ct. 2786, 12 5 L. Ed. 2 d 469.

[cl270080-bib-0141] Dawes, R. M. , D. Faust , and P. E. Meehl . 1989. “Clinical Versus Actuarial Judgment.” Science 243, no. 4899: 1668–1674. 10.1126/science.2648573. 2648573

[cl270080-bib-0142] Demant, F. , W. Wagenaar , and J. van Donselaar . 2009. Racism & Extremism Monitor: Deradicalisation in Practice. Ann Frank Stichting. https://annefrank.global.ssl.fastly.net/media/imagevault/VwhJOFqw7V26EVsFUXQu.pdf.

[cl270080-bib-0143] Desmarais, S. L. , J. Simons‐Rudolph , C. S. Brugh , E. Schilling , and C. Hoggan . 2017. “The State of Scientific Knowledge Regarding Factors Associated With Terrorism.” Journal of Threat Assessment and Management 4, no. 4: 180–209. 10.1037/tam0000090.

[cl270080-bib-0144] Douglas, K. S. , S. D. Hart , C. D. Webster , and H. Belfrage . 2013. HCR‐20V3: Assessing Risk of Violence – User Guide. Mental Health, Law, and Policy Institute, Simon Fraser University.

[cl270080-bib-0145] Douglas, K. S. , and J. L. Skeem . 2005. “Violence Risk Assessment: Getting Specific About Being Dynamic.” Psychology, Public Policy, and Law 11, no. 3: 347–383. 10.1037/1076-8971.11.3.347.

[cl270080-bib-0146] Douglas, T. , J. Pugh , I. Singh , J. Savulescu , and S. Fazel . 2017. “Risk Assessment Tools in Criminal Justice and Forensic Psychiatry: The Need for Better Data.” European Psychiatry 42: 134–137. 10.1016/j.eurpsy.2016.12.009. 28371726 PMC5408162

[cl270080-bib-0147] Duits, N. , and M. Kempes . 2023. “Interrater and Intra‐Rater Reliability of the VERA‐2R Tool.” Frontiers in Psychiatry 14: 1236295. 10.3389/fpsyt.2023.1236295. 37829765 PMC10566368

[cl270080-bib-0148] Duits, N. , C. Overdulve , and M. Kempes . 2023. “Using the VERA‐2R, Professional and Organisational Aspects.” Frontiers in Psychiatry 14: 1165279. 10.3389/fpsyt.2023.1165279. 37547204 PMC10400441

[cl270080-bib-0149] Elliott, I. A. , K. Randhawa‐Horne , and O. Hambly . 2023. *The Extremism Risk Guidance 22*+*: An Exploratory Psychometric Analysis*. Ministry of Justice Analytical Series. https://www.gov.uk/government/publications/extremism-risk-guidance-22-an-exploratory-psychometric-analysis.

[cl270080-bib-0150] Elzinga, P. , J. Poelmans , S. Viaene , G. Dedene , and S. Morsing . 2010. Terrorist Threat Assessment With Formal Concept Analysis. IEEE International Conference on Intelligence and Security Informatics. 10.1109/ISI.2010.5484773.

[cl270080-bib-0151] Emmelkamp, J. , J. J. Asscher , I. B. Wissink , and G. J. J. M. Stams . 2020. “Risk Factors for (Violent) Radicalization in Juveniles: A Multilevel Meta‐Analysis.” Aggression and Violent Behavior 55: 101489. 10.1016/j.avb.2020.101489.

[cl270080-bib-0152] Esgalhado, G. , H. Pereira , S. Monteiro , V. Costa , P. das Neves , and S. Reis . 2018. R2PRIS – Radicalisation Screening Tool. https://ec.europa.eu/programmes/erasmus-plus/project-result-content/0b4a914e-064a-4dbf-b4b0-db89bcd85c21/IO2_Eng.pdf.

[cl270080-bib-0153] Faigman, D. L. , J. Monahan , and C. Slobogin . 2014. “Group to Individual (G2i) Inference in Scientific Expert Testimony.” University of Chicago Law Review 81: 417–480. http://www.jstor.org/stable/23762370.

[cl270080-bib-0154] Faigman, D. L. , C. Slobogin , and J. Monahan . 2016. “Gatekeeping Science: Using the Structure of Scientific Research to Distinguish Between Admissibility and Weight in the Expert Testimony.” Northwestern University Law Review 110: 859–904. https://scholarlycommons.law.northwestern.edu/nulr/vol110/iss4/3.

[cl270080-bib-0155] Fawcett, T. 2006. “An Introduction to ROC Analysis.” Pattern Recognition Letters 27, no. 8: 861–874. 10.1016/j.patrec.2005.10.010.

[cl270080-bib-0156] Fazel, S. , M. Burghart , T. Fanshawe , S. D. Gil , J. Monahan , and R. Yu . 2022. “The Predictive Performance of Criminal Risk Assessment Tools Used at Sentencing: Systematic Review of Validation Studies.” Journal of Criminal Justice 81: 101902. 10.1016/j.jcrimjus.2022.101902. 36530210 PMC9755051

[cl270080-bib-0157] Fleiss, J. L. 1971. “Measuring Nominal Scale Agreement Among Many Raters.” Psychological Bulletin 76, no. 5: 378–382. 10.1037/h0031619.

[cl270080-bib-0158] Furr, R. M. 2021. Psychometrics: An introduction (4th ed.). Sage Publications.

[cl270080-bib-0159] Frye v. United States . 1923. 293 F. 1013, 34 A.L.R. 145 (D.C.Cir.).

[cl270080-bib-0160] Gendreau, P. , T. Little , and C. Goggin . 1996. “A Meta‐Analysis of the Predictors of Adult Offender Recidivism: What Works!.” Criminology 34, no. 4: 575–608. 10.1111/j.1745-9125.1996.tb01220.x.

[cl270080-bib-0161] Gill, P. , C. Clemmow , F. Hetzel , et al. 2021. “Systematic Review of Mental Health Problems and Violent Extremism.” Journal of Forensic Psychiatry & Psychology 32, no. 1: 51–78. 10.1080/14789949.2020.1820067.

[cl270080-bib-0162] Grove, W. M. , D. H. Zald , B. S. Lebow , B. E. Snitz , and C. Nelson . 2000. “Clinical Versus Mechanical Prediction: A Meta‐Analysis.” Psychological Assessment 12, no. 1: 19–30. 10.1037/1040-3590.12.1.19. 10752360

[cl270080-bib-0163] Hanson, R. K. 2009. “The Psychological Assessment of Risk for Crime and Violence.” Canadian Psychology/Psychologie canadienne 50, no. 3: 172–182. 10.1037/a0015726.

[cl270080-bib-0164] Hanson, R. K. 2021. Prediction Statistics for Psychological Assessment. American Psychological Association.

[cl270080-bib-0165] Hanson, R. K. , G. Bourgon , L. Helmus , and S. Hodgson . 2009. “The Principles of Effective Correctional Treatment Also Apply to Sexual Offenders: A Meta‐Analysis.” Criminal Justice and Behavior 36, no. 9: 865–891. 10.1177/0093854809338545.

[cl270080-bib-0166] Hanson, R. K. , and A. J. R. Harris . 2000. “Where Should We Intervene?: Dynamic Predictors of Sexual Offense Recidivism.” Criminal Justice and Behavior 27, no. 1: 6–35. 10.1177/0093854800027001002.

[cl270080-bib-0167] Hanson, R. K. , A. J. R. Harris , T.‐L. Scott , and L.‐M. Helmus . 2007. “Assessing the Risk of Sexual Offenders on Community Supervision: The Dynamic Supervision Project.” *Public Safety Canada*. https://www.publicsafety.gc.ca/cnt/rsrcs/pblctns/ssssng-rsk-sxl-ffndrs/index-en.aspx.

[cl270080-bib-0168] Harris, G. T. , M. E. Rice , and V. L. Quinsey . 1993. “Violent Recidivism of Mentally Disordered Offenders: The Development of a Statistical Prediction Instrument.” Criminal Justice and Behavior 20, no. 4: 315–335. 10.1177/0093854893020004001.

[cl270080-bib-0169] Hassan, G. , S. Brouillette‐Alarie , S. Ousman , et al. 2021. A Systematic Review on the Outcomes of Tertiary Prevention Programs in the Field of Violent Radicalization. Canadian Practitioners Network for the Prevention of Radicalization and Extremist Violence. https://cpnprev.ca/systematic-review-3/.

[cl270080-bib-0170] Hassan, G. , S. Brouillette‐Alarie , S. Ousman , et al. 2022. “Protocol: Are Tools That Assess Risk of Violent Radicalization Fit for Purpose? A Systematic Review.” Campbell Systematic Reviews 18, no. 4: e1279. 10.1002/cl2.1279. 36908841 PMC9538709

[cl270080-bib-0171] Hassan, G. , S. Ousman , P. Madriaza , et al. 2020. From Coast to Coast: Mapping of Secondary and Tertiary Prevention Initiatives in the Field of Violent Radicalization and Extremism in Canada (Report 1: Overview of Canadian organizations). Canadian Practitioners Network for the Prevention of Radicalization and Extremist Violence. https://cpnprev.ca/wp-content/uploads/2022/03/Mapping-1-EN-.pdf.

[cl270080-bib-0172] Helmus, L. M. , and K. M. Babchishin . 2017. “Primer on Risk Assessment and the Statistics Used to Evaluate Its Accuracy.” Criminal Justice and Behavior 44, no. 1: 8–25. 10.1177/0093854816678898.

[cl270080-bib-0173] Helmus, L. M. , S. M. Kelley , A. Frazier , et al. 2022. “Static‐99R: Strengths, Limitations, Predictive Accuracy Meta‐Analysis, and Legal Admissibility Review.” Psychology, Public Policy, and Law 28, no. 3: 307–331. 10.1037/law0000351.

[cl270080-bib-0174] Henriksen, K. , and H. Kaplan . 2003. “Hindsight Bias, Outcome Knowledge and Adaptive Learning.” Quality & Safety in Health Care 12 Suppl 2, no. suppl 2: 46–50. 10.1136/qhc.12.suppl_2.ii46. PMC1765779 14645895

[cl270080-bib-0175] Higgins, J. P. T. 2003. “Measuring Inconsistency in Meta‐Analyses.” BMJ 327, no. 7414: 557–560. 10.1136/bmj.327.7414.557. 12958120 PMC192859

[cl270080-bib-0176] HM Government . 2012. *Channel: Vulnerability Assessment Framework*. https://assets.publishing.service.gov.uk/media/5a7a36bee5274a34770e50fe/vul-assessment.pdf.

[cl270080-bib-0177] Hodwitz, O. 2021. “The Terrorism Recidivism Study (TRS): An Update on Data Collection and Results.” Perspectives on Terrorism 15, no. 4: 27–38. https://www.jstor.org/stable/27044233.

[cl270080-bib-0178] Ioannidis, J. P. , and T. A. Trikalinos . 2007. “An Exploratory Test for an Excess of Significant Findings.” Clinical Trials 4, no. 3: 245–253. 10.1177/1740774507079441. 17715249

[cl270080-bib-0179] Jensen, M. , and G. LaFree . 2016. *Final Report: Empirical Assessment of Domestic Radicalization* (Document Number 250481). National Criminal Justice Reference Service. https://www.ojp.gov/library/abstracts/final-report-empirical-assessment-domestic-radicalization-eadr.

[cl270080-bib-0180] Lloyd, M. 2019. *Extremism Risk Assessment: A Directory*. Centre for Research and Evidence on Security Threats (CREST). https://crestresearch.ac.uk/resources/extremism-risk-assessment-directory/.

[cl270080-bib-0181] Logan, C. , and M. Lloyd . 2019. “Violent Extremism: A Comparison of Approaches to Assessing and Managing Risk.” Legal and Criminological Psychology 24, no. 1: 141–161. 10.1111/lcrp.12140.

[cl270080-bib-0182] Lösel, F. , S. King , D. Bender , and I. Jugl . 2018. “Protective Factors Against Extremism and Violent Radicalization: A Systematic Review of Research.” International Journal of Developmental Science: Biopsychosocial Mechanisms of Change, Human Development, and Psychopathology – Perspectives From Psychology, Neuroscience, and Genetics 12, no. 1–2: 89–102. 10.3233/DEV-170241.

[cl270080-bib-0183] Lussier, P. , E. McCuish , J. Proulx , S. Chouinard Thivierge , and J. Frechette . 2023. “The Sexual Recidivism Drop in Canada: A Meta‐Analysis of Sex Offender Recidivism Rates Over an 80‐Year Period.” Criminology & Public Policy 22, no. 1: 125–160. 10.1111/1745-9133.12611.

[cl270080-bib-0184] Madriaza, P. , F. Valendru , L. Stock‐Rabbat , et al. 2018. *Rapport final du projet « Dispositif d'intervention sur la radicalisation violente en milieu ouvert: Identification des difficultés et des besoins des professionnels des SPIP, aide à l'adaptation des pratiques »* [Final Report of the Project “Intervention Measures on Violent Radicalization in the Community: Identification of the Difficulties and Needs of Professionals Working in Probation Penitentiary Services, Help in Adapting Practices”]. International Centre for the Prevention of Crime. https://www.researchgate.net/publication/325904423_Rapport_final_Dispositif_d'intervention_sur_la_radicalisation_violente_en_milieu_ouvert_SPIP_en_France.

[cl270080-bib-0185] Marshall, W. L. , and J. McGuire . 2003. “Effect Sizes in the Treatment of Sexual Offenders.” International Journal of Offender Therapy and Comparative Criminology 47, no. 6: 653–663. 10.1177/0306624X03256663. 14661385

[cl270080-bib-0186] Martinson, R. 1974. “What Works? Questions and Answers about Prison Reform.” Public Interest 35: 22–54. https://www.nationalaffairs.com/public_interest/detail/what-works-questions-and-answers-about-prison-reform.

[cl270080-bib-0187] Mathes, T. , and D. Pieper . 2019. “An Algorithm for the Classification of Study Designs to Assess Diagnostic, Prognostic and Predictive Test Accuracy in Systematic Reviews.” Systematic Reviews 8: 226. 10.1186/s13643-019-1131-4. 31481098 PMC6720081

[cl270080-bib-0188] Meloy, J. R. 2017. *TRAP‐18: Terrorist Radicalization Assessment Protocol*. Multi‐Health Systems. 10.1080/00223891.2018.1481077 29927673

[cl270080-bib-0189] Misiak, B. , J. Samochowiec , K. Bhui , et al. 2019. “A Systematic Review on the Relationship Between Mental Health, Radicalization and Mass Violence.” European Psychiatry 56, no. 1: 51–59. 10.1016/j.eurpsy.2018.11.005. 30500571

[cl270080-bib-0190] Mokkink, L. B. , H. C. W. de Vet , C. A. C. Prinsen , et al. 2018. “COSMIN Risk of Bias Checklist for Systematic Reviews of Patient‐Reported Outcome Measures.” Quality of Life Research 27, no. 5: 1171–1179. 10.1007/s11136-017-1765-4. 29260445 PMC5891552

[cl270080-bib-0191] Monahan, J. 2012. “The Individual Risk Assessment of Terrorism.” Psychology, Public Policy, and Law 18, no. 2: 167–205. 10.1037/a0025792.

[cl270080-bib-0192] Mullen, P. E. 2000. “Forensic Mental Health.” British Journal of Psychiatry 176, no. 4: 307–311. 10.1192/bjp.176.4.307. 10827876

[cl270080-bib-0193] National Offender Management Service . 2011. ERG 22+ Structured Professional Guidelines for Assessing Risk of Extremist Offending. Ministry of Justice, National Offender Management Service.

[cl270080-bib-0194] Neal, T. M. S. , C. Slobogin , M. J. Saks , D. L. Faigman , and K. F. Geisinger . 2019. “Psychological Assessments in Legal Contexts: Are Courts Keeping “Junk Science” Out of the Courtroom?.” Psychological Science in the Public Interest 20, no. 3: 135–164. 10.1177/1529100619888860. 32065036

[cl270080-bib-0195] Neumann, P. R. 2013. “The Trouble With Radicalization.” International Affairs 89, no. 4: 873–893. 10.1111/1468-2346.12049.

[cl270080-bib-0196] Olver, M. E. , K. C. Stockdale , and J. S. Wormith . 2014. “Thirty Years of Research on the Level of Service Scales: A Meta‐Analytic Examination of Predictive Accuracy and Sources of Variability.” Psychological Assessment 26, no. 1: 156–176. 10.1037/a0035080. 24274046

[cl270080-bib-0197] Pressman, D. E. , N. Duits , T. Rinne , and J. Flockton . 2016. *Violent Extremism Risk Assessment Version 2 Revised*. Netherlands Institute for Forensic Psychiatry and Psychology.

[cl270080-bib-0198] Prinsen, C. A. C. , L. B. Mokkink , L. M. Bouter , et al. 2018. “COSMIN Guideline for Systematic Reviews of Patient‐Reported Outcome Measures.” Quality of Life Research 27, no. 5: 1147–1157. 10.1007/s11136-018-1798-3. 29435801 PMC5891568

[cl270080-bib-0199] Rice, M. E. , and G. T. Harris . 2005. “Comparing Effect Sizes in Follow‐Up Studies: ROC Area, Cohen's *d*, and *r* .” Law and Human Behavior 29, no. 5: 615–620. 10.1007/s10979-005-6832-7. 16254746

[cl270080-bib-0200] Risk Management Authority . 2021. A Review of Risk Management Approaches Relevant to Terrorism and Radicalisation. https://www.rma.scot/wp-content/uploads/2022/08/A-review-of-Risk-Assessment-Tools-and-Risk-Factors-Relevant-to-Terrorism-December-2021-1.pdf.

[cl270080-bib-0201] RTI International . 2018. *Countering Violent Extremism: The Application of Risk Assessment Tools in the Criminal Justice and Rehabilitation Process* (RTI Project Number 0214428.004). First Responders Group Department of Homeland Security. https://www.dhs.gov/sites/default/files/publications/OPSR_TP_CVE-Application-Risk-Assessment-Tools-Criminal-Rehab-Process_2018Feb-508.pdf.

[cl270080-bib-0202] Sadowski, F. , H. Meier , C. Sonka , R. Witt , and J. Malzacher . 2021. “RADAR‐iTE 2.0: An Instrument of the German State Protection—Structure, Development and Current Stage of Evaluation.” In NATO Science for Peace and Security Series – E: Human and Societal Dynamics, Volume 152: Terrorism Risk Assessment Instruments, 223–235. 10.3233/NHSDP210013.

[cl270080-bib-0203] Sageman, M. 2014. “The Stagnation in Terrorism Research.” Terrorism and Political Violence 26, no. 4: 565–580. 10.1080/09546553.2014.895649.

[cl270080-bib-0204] Sarma, K. M. 2017. “Risk Assessment and the Prevention of Radicalization From Nonviolence Into Terrorism.” American Psychologist 72, no. 3: 278–288. 10.1037/amp0000121. 28383980

[cl270080-bib-0205] Scarcella, A. , R. Page , and V. Furtado . 2016. “Terrorism, Radicalisation, Extremism, Authoritarianism and Fundamentalism: A Systematic Review of the Quality and Psychometric Properties of Assessments.” PLoS One 11, no. 12: e0166947. 10.1371/journal.pone.0166947. 28002457 PMC5176288

[cl270080-bib-0206] Schmid, A. P. 2012. “The Revised Academic Consensus Definition of Terrorism.” Perspectives on Terrorism 6, no. 2: 158–159. http://www.jstor.org/stable/26298569.

[cl270080-bib-0207] Schmid, A. P. 2013. *Radicalisation, De‐Radicalisation, Counter‐Radicalisation: A Conceptual Discussion and Literature Review*. International Centre for Counter‐Terrorism. https://icct.nl/publication/radicalisation-de-radicalisation-counter-radicalisation-conceptual-discussion-and.

[cl270080-bib-0208] Schmid, A. P. 2014. *Violent and Non‐Violent Extremism: Two Sides of the Same Coin?* International Centre for Counter‐Terrorism. https://www.icct.nl/publication/violent-and-non-violent-extremism-two-sides-same-coin.

[cl270080-bib-0209] Shrout, P. E. 1998. “Measurement Reliability and Agreement in Psychiatry.” Statistical Methods in Medical Research 7, no. 3: 301–317. 10.1177/096228029800700306. 9803527

[cl270080-bib-0210] Silke, A. , and J. Morrison . 2020. *Re‐Offending by Released Terrorist Prisoners: Separating Hype From Reality*. International Centre for Counter‐Terrorism – The Hague (ICCT). https://www.icct.nl/sites/default/files/2022-12/Re-Offending-by-Released-Terrorist-Prisoners.pdf.

[cl270080-bib-0211] Singh, J. P. , M. Grann , and S. Fazel . 2011. “A Comparative Study of Violence Risk Assessment Tools: A Systematic Review and Metaregression Analysis of 68 Studies Involving 25,980 Participants.” Clinical Psychology Review 31, no. 3: 499–513. 10.1016/j.cpr.2010.11.009. 21255891

[cl270080-bib-0212] Spaaij, R. , and M. S. Hamm . 2015. “Key Issues and Research Agendas in Lone Wolf Terrorism.” Studies in Conflict & Terrorism 38, no. 3: 167–178. 10.1080/1057610X.2014.986979.

[cl270080-bib-0213] Sterne, J. A. C. , A. J. Sutton , J. P. A. Ioannidis , et al. 2011. “Recommendations for Examining and Interpreting Funnel Plot Asymmetry in Meta‐Analyses of Randomised Controlled Trials.” BMJ 343: d4002. 10.1136/bmj.d4002. 21784880

[cl270080-bib-0214] Sun, S. 2011. “Meta‐Analysis of Cohen's Kappa.” Health Services and Outcomes Research Methodology 11, no. 3: 145–163. 10.1007/s10742-011-0077-3.

[cl270080-bib-0215] Sweeting, M. J. , A. J. Sutton , and P. C. Lambert . 2004. “What to Add to Nothing? Use and Avoidance of Continuity Corrections in Meta‐Analysis of Sparse Data.” Statistics in Medicine 23, no. 9: 1351–1375. 10.1002/sim.1761. 15116347

[cl270080-bib-0216] Swets, J. A. 1988. “Measuring the Accuracy of Diagnostic Systems.” Science 240, no. 4857: 1285–1293. 10.1126/science.3287615. 3287615

[cl270080-bib-0217] Tanguy, B. , S. Dercon , K. Orkin , and A. S. Taffesse . 2014. The Future in Mind: Aspirations and Forward‐Looking Behaviour in Rural Ethiopia (Publication No. WPS/2014–16). Centre for the Study of African Economies.

[cl270080-bib-0218] Tassin, C. , and C. S. Allely . 2022. “Application of the Terrorist Radicalization Assessment Protocol (TRAP‐18) to the Case of the Army‐Navy Recruiting Center Attacker in Little Rock, Arkansas.” Behavioral Sciences of Terrorism and Political Aggression 16, no. 3: 402–427. 10.1080/19434472.2022.2118349.

[cl270080-bib-0219] Tavakol, M. , and R. Dennick . 2011. “Making Sense of Cronbach's Alpha.” International Journal of Medical Education 2: 53–55. 10.5116/ijme.4dfb.8dfd. 28029643 PMC4205511

[cl270080-bib-0220] Terwee, C. B. , C. A. C. Prinsen , A. Chiarotto , et al. 2018. “COSMIN Methodology for Evaluating the Content Validity of Patient‐Reported Outcome Measures: A Delphi Study.” Quality of Life Research 27, no. 5: 1159–1170. 10.1007/s11136-018-1829-0. 29550964 PMC5891557

[cl270080-bib-0221] van der Heide, E. , and B. Schuurman . 2018. “Reintegrating Terrorists in the Netherlands: Evaluating the Dutch Approach.” Journal for Deradicalization 17: 196–239. https://journals.sfu.ca/jd/index.php/jd/article/view/179.

[cl270080-bib-0222] van der Heide, L. , M. van der Zwan , and M. van Leyenhorst . 2019. *The Practitioner's Guide to the Galaxy—A Comparison of Risk Assessment Tools for Violent Extremism*. International Centre for Counter‐Terrorism—The Hague. https://icct.nl/publication/the-practitioners-guide-to-the-galaxy-a-comparison-of-risk-assessment-tools-for-violent-extremism/.

[cl270080-bib-0223] Vergani, M. , M. Iqbal , E. Ilbahar , and G. Barton . 2020. “The Three Ps of Radicalization: Push, Pull and Personal. A Systematic Scoping Review of the Scientific Evidence About Radicalization Into Violent Extremism.” Studies in Conflict & Terrorism 43, no. 10: 854. 10.1080/1057610X.2018.1505686.

[cl270080-bib-0224] Viljoen, J. L. , I. Goossens , S. Monjazeb , et al. 2025. “Are Risk Assessment Tools More Accurate Than Unstructured Judgments in Predicting Violent, Any, and Sexual Offending? A Meta‐Analysis of Direct Comparison Studies.” Behavioral Sciences & the Law 43, no. 1: 75–113. 10.1002/bsl.2698. 39363308 PMC11771637

[cl270080-bib-0225] Webster, C. D. , T. L. Nicholls , M. L. Martin , S. L. Desmarais , and J. Brink . 2006. “Short‐Term Assessment of Risk and Treatability (START): The Case for a New Structured Professional Judgment Scheme.” Behavioral Sciences & the Law 24, no. 6: 747–766. 10.1002/bsl.737. 17171764

[cl270080-bib-0226] Wolfowicz, M. , Y. Litmanovitz , D. Weisburd , and B. Hasisi . 2020. “A Field‐Wide Systematic Review and Meta‐Analysis of Putative Risk and Protective Factors for Radicalization Outcomes.” Journal of Quantitative Criminology 36: 407–447. 10.1007/s10940-019-09439-4.

