# Supplementary material for: Reliability and Validity of Risk Assessment Tools for Violent Extremism: A Systematic Review
Source: Campbell Syst Rev. 2025 Dec 7;21(4):e70080. doi: 10.1002/cl2.70080 (PMC12682207; doi:10.1002/cl2.70080)
Supplement: Supplementary file 1 — Appendices. [file CL2-21-e70080-s001.pdf]

Appendix A

Search Strategy and Phases

Violent Extremism Risk Assessment Tools Review – Initial Search (Phase 1)

<u>Overview</u>

Source	Initial results	Results after duplicates
PsycINFO	1,090	1,089
ERIC	46	36
Education Source	81	56
Academic Search Complete	707	545
Sociological Abstracts	242	167
Criminal Justice Abstracts	238	60
NCJRS	170	155
ProQuest Central	528	250
ProQuest Dissertations & Theses Global	128	87
Medline	1,744	1,254
Web of Science	1,251	860
**TOTAL**	**6,225**	**4,559**

<u>PsycINFO (APA PsycNET)</u>
Search performed September 25, 2020

1,090 Results for ((**title**: ("Activism and Radicalism Intention Scales") *OR* **title**: ("Building Resilience to Violent Extremism") *OR* **title**: ("BRAVE 14") *OR* **title**: (BRAVE14) *OR* **title**: ("Extremism Monitoring Instrument") *OR* **title**: ("EMI 20") *OR* **title**: (EMI20) *OR* **title**: ("Extremism Risk Guidance") *OR* **title**: ("Extremism Risk Guidelines") *OR* **title**: ("ERG 22+") *OR* **title**: (ERG22+) *OR* **title**: ("IAT 8") *OR* **title**: (IAT8) *OR* **title**: ("Identifying Vulnerable People Guidance") *OR* **title**: ("Intratextual Fundamentalism Scale") *OR* **title**: ("IR 46") *OR* **title**: (IR46) *OR* **title**: ("Militant Extremist Mindset") *OR* **title**: ("Multi level Guidelines") *OR* **title**: ("Multi-Dimensional Fundamentalism Inventory") *OR* **title**: ("Radicalisation Risk Assessment in Prisons") *OR* **title**: ("Référentiel des indicateurs de basculement dans la radicalisation") *OR* **title**: ("Religious Fundamentalism Scale") *OR* **title**: ("Significance Quest Assessment Tool") *OR* **title**: ("Significance Quest Assessment Test") *OR* **title**: (SyFoR) *OR* **title**: ("Terrorist Radicalization Assessment Protocol") *OR* **title**: ("TRAP 18") *OR* **title**: (TRAP18) *OR* **title**: ("Violence Threat Risk Assessment") *OR* **title**: ("Violent Extremism Beliefs Scale") *OR* **title**: ("Violent Extremism Risk Assessment") *OR* **title**: ("VERA 2") *OR* **title**: (VERA2*) *OR* **title**: ("Vulnerability Assessment Framework")) *OR* (**abstract**: ("Activism and Radicalism Intention Scales") *OR* **abstract**: ("Building Resilience to Violent Extremism") *OR* **abstract**: ("BRAVE 14") *OR* **abstract**: (BRAVE14) *OR* **abstract**: ("Extremism Monitoring Instrument") *OR* **abstract**: ("EMI 20") *OR* **abstract**: (EMI20) *OR* **abstract**: ("Extremism Risk Guidance") *OR* **abstract**: ("Extremism Risk Guidelines") *OR* **abstract**: ("ERG 22+") *OR* **abstract**:

(ERG22+) *OR* **abstract**: ("IAT 8") *OR* **abstract**: (IAT8) *OR* **abstract**: ("Identifying Vulnerable People Guidance") *OR* **abstract**: ("Intratextual Fundamentalism Scale") *OR* **abstract**: ("IR 46") *OR* **abstract**: (IR46) *OR* **abstract**: ("Militant Extremist Mindset") *OR* **abstract**: ("Multi level Guidelines") *OR* **abstract**: ("Multi-Dimensional Fundamentalism Inventory") *OR* **abstract**: ("Radicalisation Risk Assessment in Prisons") *OR* **abstract**: ("Référentiel des indicateurs de basculement dans la radicalisation") *OR* **abstract**: ("Religious Fundamentalism Scale") *OR* **abstract**: ("Significance Quest Assessment Tool") *OR* **abstract**: ("Significance Quest Assessment Test") *OR* **abstract**: (SyFoR) *OR* **abstract**: ("Terrorist Radicalization Assessment Protocol") *OR* **abstract**: ("TRAP 18") *OR* **abstract**: (TRAP18) *OR* **abstract**: ("Violence Threat Risk Assessment") *OR* **abstract**: ("Violent Extremism Beliefs Scale") *OR* **abstract**: ("Violent Extremism Risk Assessment") *OR* **abstract**: ("VERA 2") *OR* **abstract**: (VERA2*) *OR* **abstract**: ("Vulnerability Assessment Framework")) *OR* (**Keywords**: ("Activism and Radicalism Intention Scales") *OR* **Keywords**: ("Building Resilience to Violent Extremism") *OR* **Keywords**: ("BRAVE 14") *OR* **Keywords**: (BRAVE14) *OR* **Keywords**: ("Extremism Monitoring Instrument") *OR* **Keywords**: ("EMI 20") *OR* **Keywords**: (EMI20) *OR* **Keywords**: ("Extremism Risk Guidance") *OR* **Keywords**: ("Extremism Risk Guidelines") *OR* **Keywords**: ("ERG 22+") *OR* **Keywords**: (ERG22+) *OR* **Keywords**: ("IAT 8") *OR* **Keywords**: (IAT8) *OR* **Keywords**: ("Identifying Vulnerable People Guidance") *OR* **Keywords**: ("Intratextual Fundamentalism Scale") *OR* **Keywords**: ("IR 46") *OR* **Keywords**: (IR46) *OR* **Keywords**: ("Militant Extremist Mindset") *OR* **Keywords**: ("Multi level Guidelines") *OR* **Keywords**: ("Multi-Dimensional Fundamentalism Inventory") *OR* **Keywords**: ("Radicalisation Risk Assessment in Prisons") *OR* **Keywords**: ("Référentiel des indicateurs de basculement dans la radicalisation") *OR* **Keywords**: ("Religious Fundamentalism Scale") *OR* **Keywords**: ("Significance Quest Assessment Tool") *OR* **Keywords**: ("Significance Quest Assessment Test") *OR* **Keywords**: (SyFoR) *OR* **Keywords**: ("Terrorist Radicalization Assessment Protocol") *OR* **Keywords**: ("TRAP 18") *OR* **Keywords**: (TRAP18) *OR* **Keywords**: ("Violence Threat Risk Assessment") *OR* **Keywords**: ("Violent Extremism Beliefs Scale") *OR* **Keywords**: ("Violent Extremism Risk Assessment") *OR* **Keywords**: ("VERA 2") *OR* **Keywords**: (VERA2*) *OR* **Keywords**: ("Vulnerability Assessment Framework"))) *OR* ((((**Keywords**: (radicali*)) *OR* (**Keywords**: (extremis*)) *OR* (**Keywords**: (fundamentalis*)) *OR* (**Keywords**: (terroris*)) *OR* (**Keywords**: ("hate crime*")) *OR* (**Keywords**: ("religious violence*")) *OR* (**Keywords**: ("political violence*")) *OR* (**Keywords**: ("ideological violence*")) *OR* (**Keywords**: ("environmental violence*")) *OR* (**Keywords**: ("racist violence*")) *OR* (**Keywords**: ("separatist violence*")) *OR* (**Keywords**: ("far right")) *OR* (**Keywords**: ("right wing")) *OR* (**Keywords**: ("alt right")) *OR* (**Keywords**: ("radical right")) *OR* (**Keywords**: ("extreme right")) *OR* (**Keywords**: ("white supremac*")) *OR* (**Keywords**: ("neo nazi*")) *OR* (**Keywords**: (neonazi*)) *OR* (**Keywords**: ("anti semiti*")) *OR* (**Keywords**: (antisemiti*)) *OR* (**Keywords**: ("left wing")) *OR* (**Keywords**: ("far left")) *OR* (**Keywords**: ("alt left")) *OR* (**Keywords**: ("anti fa")) *OR* (**Keywords**: (antifa*)) *OR* (**Keywords**: (anarch*)) *OR* (**Keywords**: ("anti capitalis*")) *OR* (**Keywords**: (anticapitalis*)) *OR* (**Keywords**: (jihadis*)) *OR* (**Keywords**: (islamis*)) *OR* (**Keywords**: (salafis*)) *OR* (**Keywords**: ("lone wolf*")) *OR* (**Keywords**: ("lone actor*")) *OR* (**Keywords**: ("lone offend*")) *OR* (**Keywords**: ("suicide bomb*")) *OR* (**Keywords**: ("suicide attack*")) *OR* (**Keywords**: ("mass shoot*")) *OR* (**Keywords**: (indoctrinat*)) *OR* (**Keywords**: ("foreign fight*")) *OR* (**Keywords**: (martyr*))) *OR* ((**title**: (radicali*)) *OR* (**title**:

(extremis*)) *OR* (**title**: (fundamentalis*)) *OR* (**title**: (terroris*)) *OR* (**title**: ("hate crime*")) *OR* (**title**: ("religious violence*")) *OR* (**title**: ("political violence*")) *OR* (**title**: ("ideological violence*")) *OR* (**title**: ("environmental violence*")) *OR* (**title**: ("racist violence*")) *OR* (**title**: ("separatist violence*")) *OR* (**title**: ("far right")) *OR* (**title**: ("right wing")) *OR* (**title**: ("alt right")) *OR* (**title**: ("radical right")) *OR* (**title**: ("extreme right")) *OR* (**title**: ("white supremac*")) *OR* (**title**: ("neo nazi*")) *OR* (**title**: (neonazi*)) *OR* (**title**: ("anti semiti*")) *OR* (**title**: (antisemiti*)) *OR* (**title**: ("left wing")) *OR* (**title**: ("far left")) *OR* (**title**: ("alt left")) *OR* (**title**: ("anti fa")) *OR* (**title**: (antifa*)) *OR* (**title**: (anarch*)) *OR* (**title**: ("anti capitalis*")) *OR* (**title**: (anticapitalis*)) *OR* (**title**: (jihadis*)) *OR* (**title**: (islamis*)) *OR* (**title**: (salafis*)) *OR* (**title**: ("lone wolf*")) *OR* (**title**: ("lone actor*")) *OR* (**title**: ("lone offend*")) *OR* (**title**: ("suicide bomb*")) *OR* (**title**: ("suicide attack*")) *OR* (**title**: ("mass shoot*")) *OR* (**title**: (indoctrinat*)) *OR* (**title**: ("foreign fight*")) *OR* (**title**: (martyr*))) *OR* ((**abstract**: (radicali*)) *OR* (**abstract**: (extremis*)) *OR* (**abstract**: (fundamentalis*)) *OR* (**abstract**: (terroris*)) *OR* (**abstract**: ("hate crime*")) *OR* (**abstract**: ("religious violence*")) *OR* (**abstract**: ("political violence*")) *OR* (**abstract**: ("ideological violence*")) *OR* (**abstract**: ("environmental violence*")) *OR* (**abstract**: ("racist violence*")) *OR* (**abstract**: ("separatist violence*")) *OR* (**abstract**: ("far right")) *OR* (**abstract**: ("right wing")) *OR* (**abstract**: ("alt right")) *OR* (**abstract**: ("radical right")) *OR* (**abstract**: ("extreme right")) *OR* (**abstract**: ("white supremac*")) *OR* (**abstract**: ("neo nazi*")) *OR* (**abstract**: (neonazi*)) *OR* (**abstract**: ("anti semiti*")) *OR* (**abstract**: (antisemiti*)) *OR* (**abstract**: ("left wing")) *OR* (**abstract**: ("far left")) *OR* (**abstract**: ("alt left")) *OR* (**abstract**: ("anti fa")) *OR* (**abstract**: (antifa*)) *OR* (**abstract**: (anarch*)) *OR* (**abstract**: ("anti capitalis*")) *OR* (**abstract**: (anticapitalis*)) *OR* (**abstract**: (jihadis*)) *OR* (**abstract**: (islamis*)) *OR* (**abstract**: (salafis*)) *OR* (**abstract**: ("lone wolf*")) *OR* (**abstract**: ("lone actor*")) *OR* (**abstract**: ("lone offend*")) *OR* (**abstract**: ("suicide bomb*")) *OR* (**abstract**: ("suicide attack*")) *OR* (**abstract**: ("mass shoot*")) *OR* (**abstract**: (indoctrinat*)) *OR* (**abstract**: ("foreign fight*")) *OR* (**abstract**: (martyr*)))) *AND* (((((((**Keywords**: (risk*)))) *OR* (((**Keywords**: (vulnerabilit*)))) *OR* (((**Keywords**: (recidiv*)))) *OR* (((**Keywords**: (dangerousness)))) *OR* (((**Keywords**: ("risk factor*")))) *OR* (((**Keywords**: ("risk indicator*"))))) *AND* ((((**Keywords**: (assess*)))) *OR* (((**Keywords**: (predict*)))) *OR* (((**Keywords**: (evaluat*)))) *OR* (((**Keywords**: (screening*)))) *OR* (((**Keywords**: (tool*)))) *OR* (((**Keywords**: (protocol*)))) *OR* (((**Keywords**: (scale*)))) *OR* (((**Keywords**: (instrument*)))))) *OR* (((((**abstract**: (risk*)))) *OR* (((**abstract**: (vulnerabilit*)))) *OR* (((**abstract**: (recidiv*)))) *OR* (((**abstract**: (dangerousness)))) *OR* (((**abstract**: ("risk factor*")))) *OR* (((**abstract**: ("risk indicator*"))))) *AND* ((((**abstract**: (assess*)))) *OR* (((**abstract**: (predict*)))) *OR* (((**abstract**: (evaluat*)))) *OR* (((**abstract**: (screening*)))) *OR* (((**abstract**: (tool*)))) *OR* (((**abstract**: (protocol*)))) *OR* (((**abstract**: (scale*)))) *OR* (((**abstract**: (instrument*)))))) *OR* (((((**title**: (risk*)))) *OR* (((**title**: (vulnerabilit*)))) *OR* (((**title**: (recidiv*)))) *OR* (((**title**: (dangerousness)))) *OR* (((**title**: ("risk factor*")))) *OR* (((**title**: ("risk indicator*"))))) *AND* ((((**title**: (assess*)))) *OR* (((**title**: (predict*)))) *OR* (((**title**: (evaluat*)))) *OR* (((**title**: (screening*)))) *OR* (((**title**: (tool*)))) *OR* (((**title**: (protocol*)))) *OR* (((**title**: (scale*)))) *OR* (((**title**: (instrument*))))))) *OR* ((((**title**: ("need* assessment*"))) *OR* ((**title**: ("assessment* of need*"))) *OR* ((**title**: ("need* evaluation*"))) *OR* ((**title**: ("evaluation* of need*"))) *OR* ((**title**: ("need* screening*"))) *OR* ((**title**: ("screening* of need*"))) *OR* ((**title**: ("threat*

assessment*"))) *OR* ((**title**: ("assessment* of threat*"))) *OR* ((**title**: ("threat* prediction*"))) *OR* ((**title**: ("prediction* of threat*"))) *OR* ((**title**: ("risk* calibration*"))) *OR* ((**title**: ("calibration* of risk*"))) *OR* ((**title**: ("vulnerabilit* index*"))) *OR* ((**title**: ("index* of vulnerabilit*"))) *OR* ((**title**: ("risk* measur*"))) *OR* ((**title**: ("measur* of risk*"))) *OR* ((**title**: ("vulnerability* measur*"))) *OR* ((**title**: ("measur* of vulnerability*"))) *OR* ((**title**: ("recidivism* measur*"))) *OR* ((**title**: ("measur* of recidivism*"))) *OR* ((**title**: ("dangerousness measur*"))) *OR* ((**title**: ("measur* of dangerousness")))) *OR* (((**Keywords**: ("need* assessment*"))) *OR* ((**Keywords**: ("assessment* of need*"))) *OR* ((**Keywords**: ("need* evaluation*"))) *OR* ((**Keywords**: ("evaluation* of need*"))) *OR* ((**Keywords**: ("need* screening*"))) *OR* ((**Keywords**: ("screening* of need*"))) *OR* ((**Keywords**: ("threat* assessment*"))) *OR* ((**Keywords**: ("assessment* of threat*"))) *OR* ((**Keywords**: ("threat* prediction*"))) *OR* ((**Keywords**: ("prediction* of threat*"))) *OR* ((**Keywords**: ("risk* calibration*"))) *OR* ((**Keywords**: ("calibration* of risk*"))) *OR* ((**Keywords**: ("vulnerabilit* index*"))) *OR* ((**Keywords**: ("index* of vulnerabilit*"))) *OR* ((**Keywords**: ("risk* measur*"))) *OR* ((**Keywords**: ("measur* of risk*"))) *OR* ((**Keywords**: ("vulnerability* measur*"))) *OR* ((**Keywords**: ("measur* of vulnerability*"))) *OR* ((**Keywords**: ("recidivism* measur*"))) *OR* ((**Keywords**: ("measur* of recidivism*"))) *OR* ((**Keywords**: ("dangerousness measur*"))) *OR* ((**Keywords**: ("measur* of dangerousness")))) *OR* (((**abstract**: ("need* assessment*"))) *OR* ((**abstract**: ("assessment* of need*"))) *OR* ((**abstract**: ("need* evaluation*"))) *OR* ((**abstract**: ("evaluation* of need*"))) *OR* ((**abstract**: ("need* screening*"))) *OR* ((**abstract**: ("screening* of need*"))) *OR* ((**abstract**: ("threat* assessment*"))) *OR* ((**abstract**: ("assessment* of threat*"))) *OR* ((**abstract**: ("threat* prediction*"))) *OR* ((**abstract**: ("prediction* of threat*"))) *OR* ((**abstract**: ("risk* calibration*"))) *OR* ((**abstract**: ("calibration* of risk*"))) *OR* ((**abstract**: ("vulnerabilit* index*"))) *OR* ((**abstract**: ("index* of vulnerabilit*"))) *OR* ((**abstract**: ("risk* measur*"))) *OR* ((**abstract**: ("measur* of risk*"))) *OR* ((**abstract**: ("vulnerability* measur*"))) *OR* ((**abstract**: ("measur* of vulnerability*"))) *OR* ((**abstract**: ("recidivism* measur*"))) *OR* ((**abstract**: ("measur* of recidivism*"))) *OR* ((**abstract**: ("dangerousness measur*"))) *OR* ((**abstract**: ("measur* of dangerousness")))))))

RESULTS: 1,090 (1,089 after duplicates)

<u>ERIC (EBSCO)</u>
Search performed October 26, 2020

S1 TI ((risk* OR vulnerabilit* OR recidiv* OR dangerousness OR "risk factor*" OR "risk indicator*") N2 (assess* OR predict* OR evaluat* OR screening* OR tool* OR protocol* OR scale* OR instrument*)) OR AB ((risk* OR vulnerabilit* OR recidiv* OR dangerousness OR "risk factor*" OR "risk indicator*") N2 (assess* OR predict* OR evaluat* OR screening* OR tool* OR protocol* OR scale* OR instrument*)) OR SU ((risk* OR vulnerabilit* OR recidiv* OR dangerousness OR "risk factor*" OR "risk indicator*") N2 (assess* OR predict* OR evaluat* OR screening* OR tool* OR protocol* OR scale* OR instrument*))

S2 TI ("need* assessment*" OR "assessment* of need*" OR "need* evaluation*" OR "evaluation* of need*" OR "need* screening*" OR "screening* of need*" OR "threat*

assessment*" OR "assessment* of threat*" OR "threat* prediction*" OR "prediction* of threat*" OR "risk* calibration*" OR "calibration* of risk*" OR "vulnerabilit* index*" OR "index* of vulnerabilit*" OR "risk* measur*" OR "measur* of risk*" OR "vulnerability* measur*" OR "measur* of vulnerability*" OR "recidivism* measur*" OR "measur* of recidivism*" OR "dangerousness measur*" OR "measur* of dangerousness") OR AB ("need* assessment*" OR "assessment* of need*" OR "need* evaluation*" OR "evaluation* of need*" OR "need* screening*" OR "screening* of need*" OR "threat* assessment*" OR "assessment* of threat*" OR "threat* prediction*" OR "prediction* of threat*" OR "risk* calibration*" OR "calibration* of risk*" OR "vulnerabilit* index*" OR "index* of vulnerabilit*" OR "risk* measur*" OR "measur* of risk*" OR "vulnerability* measur*" OR "measur* of vulnerability*" OR "recidivism* measur*" OR "measur* of recidivism*" OR "dangerousness measur*" OR "measur* of dangerousness") OR SU ("need* assessment*" OR "assessment* of need*" OR "need* evaluation*" OR "evaluation* of need*" OR "need* screening*" OR "screening* of need*" OR "threat* assessment*" OR "assessment* of threat*" OR "threat* prediction*" OR "prediction* of threat*" OR "risk* calibration*" OR "calibration* of risk*" OR "vulnerabilit* index*" OR "index* of vulnerabilit*" OR "risk* measur*" OR "measur* of risk*" OR "vulnerability* measur*" OR "measur* of vulnerability*" OR "recidivism* measur*" OR "measur* of recidivism*" OR "dangerousness measur*" OR "measur* of dangerousness")

S3 S1 OR S2

S4 TI (radicali* OR extremis* OR fundamentalis* OR terroris* OR “hate crime*” OR "religious violence*" OR "political violence*" OR "ideological violence*" OR "environmental violence*" OR "racist violence*" OR "separatist violence*" OR "far right" OR "right wing" OR "alt right" OR "radical right" OR "extreme right" OR "white supremac*" OR "neo nazi*" OR neonazi* OR "anti semiti*" OR antisemiti* OR "left wing" OR "far left" OR "alt left" OR "anti fa" OR antifa* OR anarch* OR "anti capitalis*" OR anticapitalis* OR jihadis* OR islamis* OR salafis* OR "lone wolf*" OR "lone actor*" OR "lone offend*" OR "suicide bomb*" OR "suicide attack*" OR "mass shoot*" OR indoctrinat* OR "foreign fight*" OR martyr*) OR AB (radicali* OR extremis* OR fundamentalis* OR terroris* OR “hate crime*” OR "religious violence*" OR "political violence*" OR "ideological violence*" OR "environmental violence*" OR "racist violence*" OR "separatist violence*" OR "far right" OR "right wing" OR "alt right" OR "radical right" OR "extreme right" OR "white supremac*" OR "neo nazi*" OR neonazi* OR "anti semiti*" OR antisemiti* OR "left wing" OR "far left" OR "alt left" OR "anti fa" OR antifa* OR anarch* OR "anti capitalis*" OR anticapitalis* OR jihadis* OR islamis* OR salafis* OR "lone wolf*" OR "lone actor*" OR "lone offend*" OR "suicide bomb*" OR "suicide attack*" OR "mass shoot*" OR indoctrinat* OR "foreign fight*" OR martyr*) OR SU (radicali* OR extremis* OR fundamentalis* OR terroris* OR “hate crime*” OR "religious violence*" OR "political violence*" OR "ideological violence*" OR "environmental violence*" OR "racist violence*" OR "separatist violence*" OR "far right" OR "right wing" OR "alt right" OR "radical right" OR "extreme right" OR "white supremac*" OR "neo nazi*" OR neonazi* OR "anti semiti*" OR antisemiti* OR "left wing" OR "far left" OR "alt left" OR "anti fa" OR antifa* OR anarch* OR "anti capitalis*" OR anticapitalis* OR jihadis* OR islamis* OR salafis* OR "lone wolf*" OR "lone actor*" OR "lone offend*" OR "suicide bomb*" OR "suicide attack*" OR "mass shoot*" OR indoctrinat* OR "foreign fight*" OR martyr*)

S5 S3 AND S4

S6 "Activism and Radicalism Intention Scales" OR "Building Resilience to Violent Extremism" OR "BRAVE 14" OR BRAVE14 OR "Extremism Monitoring Instrument" OR "EMI 20" OR EMI20 OR "Extremism Risk Guidance" OR "Extremism Risk Guidelines" OR "ERG 22+" OR ERG22+ OR "IAT 8" OR IAT8 OR "Identifying Vulnerable People Guidance" OR "Intratextual Fundamentalism Scale" OR "IR 46" OR IR46 OR "Militant Extremist Mindset" OR "Multi level Guidelines" OR "Multi-Dimensional Fundamentalism Inventory" OR "Radicalisation Risk Assessment in Prisons" OR "Référentiel des indicateurs de basculement dans la radicalisation" OR "Religious Fundamentalism Scale" OR "Significance Quest Assessment Tool" OR "Significance Quest Assessment Test" OR SyFoR OR "Terrorist Radicalization Assessment Protocol" OR "TRAP 18" OR TRAP18 OR "Violence Threat Risk Assessment" OR "Violent Extremism Beliefs Scale" OR "Violent Extremism Risk Assessment" OR "VERA 2" OR VERA2* OR "Vulnerability Assessment Framework"

S7 S5 OR S6

RESULTS: 46 records (36 after duplicates)

Education Source (EBSCO)
Search performed October 26, 2020

S1 TI ((risk* OR vulnerabilit* OR recidiv* OR dangerousness OR "risk factor*" OR "risk indicator*") N2 (assess* OR predict* OR evaluat* OR screening* OR tool* OR protocol* OR scale* OR instrument*)) OR AB ((risk* OR vulnerabilit* OR recidiv* OR dangerousness OR "risk factor*" OR "risk indicator*") N2 (assess* OR predict* OR evaluat* OR screening* OR tool* OR protocol* OR scale* OR instrument*)) OR SU ((risk* OR vulnerabilit* OR recidiv* OR dangerousness OR "risk factor*" OR "risk indicator*") N2 (assess* OR predict* OR evaluat* OR screening* OR tool* OR protocol* OR scale* OR instrument*))

S2 TI ("need* assessment*" OR "assessment* of need*" OR "need* evaluation*" OR "evaluation* of need*" OR "need* screening*" OR "screening* of need*" OR "threat* assessment*" OR "assessment* of threat*" OR "threat* prediction*" OR "prediction* of threat*" OR "risk* calibration*" OR "calibration* of risk*" OR "vulnerabilit* index*" OR "index* of vulnerabilit*" OR "risk* measur*" OR "measur* of risk*" OR "vulnerability* measur*" OR "measur* of vulnerability*" OR "recidivism* measur*" OR "measur* of recidivism*" OR "dangerousness measur*" OR "measur* of dangerousness") OR AB ("need* assessment*" OR "assessment* of need*" OR "need* evaluation*" OR "evaluation* of need*" OR "need* screening*" OR "screening* of need*" OR "threat* assessment*" OR "assessment* of threat*" OR "threat* prediction*" OR "prediction* of threat*" OR "risk* calibration*" OR "calibration* of risk*" OR "vulnerabilit* index*" OR "index* of vulnerabilit*" OR "risk* measur*" OR "measur* of risk*" OR "vulnerability* measur*" OR "measur* of vulnerability*" OR "recidivism* measur*" OR "measur* of recidivism*" OR "dangerousness measur*" OR "measur* of dangerousness") OR SU ("need* assessment*" OR "assessment* of need*" OR "need* evaluation*" OR "evaluation* of need*" OR "need* screening*" OR "screening* of need*" OR "threat* assessment*" OR "assessment* of threat*" OR "threat* prediction*" OR

"prediction* of threat*" OR "risk* calibration*" OR "calibration* of risk*" OR "vulnerabilit* index*" OR "index* of vulnerabilit*" OR "risk* measur*" OR "measur* of risk*" OR "vulnerability* measur*" OR "measur* of vulnerability*" OR "recidivism* measur*" OR "measur* of recidivism*" OR "dangerousness measur*" OR "measur* of dangerousness")

S3 S1 OR S2

S4 TI (radicali* OR extremis* OR fundamentalis* OR terroris* OR “hate crime*” OR "religious violence*" OR "political violence*" OR "ideological violence*" OR "environmental violence*" OR "racist violence*" OR "separatist violence*" OR "far right" OR "right wing" OR "alt right" OR "radical right" OR "extreme right" OR "white supremac*" OR "neo nazi*" OR neonazi* OR "anti semiti*" OR antisemiti* OR "left wing" OR "far left" OR "alt left" OR "anti fa" OR antifa* OR anarch* OR "anti capitalis*" OR anticapitalis* OR jihadis* OR islamis* OR salafis* OR "lone wolf*" OR "lone actor*" OR "lone offend*" OR "suicide bomb*" OR "suicide attack*" OR "mass shoot*" OR indoctrinat* OR "foreign fight*" OR martyr*) OR AB (radicali* OR extremis* OR fundamentalis* OR terroris* OR “hate crime*” OR "religious violence*" OR "political violence*" OR "ideological violence*" OR "environmental violence*" OR "racist violence*" OR "separatist violence*" OR "far right" OR "right wing" OR "alt right" OR "radical right" OR "extreme right" OR "white supremac*" OR "neo nazi*" OR neonazi* OR "anti semiti*" OR antisemiti* OR "left wing" OR "far left" OR "alt left" OR "anti fa" OR antifa* OR anarch* OR "anti capitalis*" OR anticapitalis* OR jihadis* OR islamis* OR salafis* OR "lone wolf*" OR "lone actor*" OR "lone offend*" OR "suicide bomb*" OR "suicide attack*" OR "mass shoot*" OR indoctrinat* OR "foreign fight*" OR martyr*) OR SU (radicali* OR extremis* OR fundamentalis* OR terroris* OR “hate crime*” OR "religious violence*" OR "political violence*" OR "ideological violence*" OR "environmental violence*" OR "racist violence*" OR "separatist violence*" OR "far right" OR "right wing" OR "alt right" OR "radical right" OR "extreme right" OR "white supremac*" OR "neo nazi*" OR neonazi* OR "anti semiti*" OR antisemiti* OR "left wing" OR "far left" OR "alt left" OR "anti fa" OR antifa* OR anarch* OR "anti capitalis*" OR anticapitalis* OR jihadis* OR islamis* OR salafis* OR "lone wolf*" OR "lone actor*" OR "lone offend*" OR "suicide bomb*" OR "suicide attack*" OR "mass shoot*" OR indoctrinat* OR "foreign fight*" OR martyr*)

S5 S3 AND S4

S6 "Activism and Radicalism Intention Scales" OR "Building Resilience to Violent Extremism" OR "BRAVE 14" OR BRAVE14 OR "Extremism Monitoring Instrument" OR "EMI 20" OR EMI20 OR "Extremism Risk Guidance" OR "Extremism Risk Guidelines" OR "ERG 22+" OR ERG22+ OR "IAT 8" OR IAT8 OR "Identifying Vulnerable People Guidance" OR "Intratextual Fundamentalism Scale" OR "IR 46" OR IR46 OR "Militant Extremist Mindset" OR "Multi level Guidelines" OR "Multi-Dimensional Fundamentalism Inventory" OR "Radicalisation Risk Assessment in Prisons" OR "Référentiel des indicateurs de basculement dans la radicalisation" OR "Religious Fundamentalism Scale" OR "Significance Quest Assessment Tool" OR "Significance Quest Assessment Test" OR SyFoR OR "Terrorist Radicalization Assessment Protocol" OR "TRAP 18" OR TRAP18 OR "Violence Threat Risk Assessment" OR "Violent Extremism Beliefs Scale" OR "Violent Extremism Risk Assessment" OR "VERA 2" OR VERA2* OR "Vulnerability Assessment Framework"

S7 S5 OR S6
Filtered: Magazines (31), Newspapers (2), Book Reviews (2)

RESULTS: 81 records (56 after duplicates)
Academic Search Complete (EBSCO)
Search performed October 26, 2020

S1 TI ((risk* OR vulnerabilit* OR recidiv* OR dangerousness OR "risk factor*" OR "risk indicator*") N2 (assess* OR predict* OR evaluat* OR screening* OR tool* OR protocol* OR scale* OR instrument*)) OR AB ((risk* OR vulnerabilit* OR recidiv* OR dangerousness OR "risk factor*" OR "risk indicator*") N2 (assess* OR predict* OR evaluat* OR screening* OR tool* OR protocol* OR scale* OR instrument*)) OR SU ((risk* OR vulnerabilit* OR recidiv* OR dangerousness OR "risk factor*" OR "risk indicator*") N2 (assess* OR predict* OR evaluat* OR screening* OR tool* OR protocol* OR scale* OR instrument*))

S2 TI ("need* assessment*" OR "assessment* of need*" OR "need* evaluation*" OR "evaluation* of need*" OR "need* screening*" OR "screening* of need*" OR "threat* assessment*" OR "assessment* of threat*" OR "threat* prediction*" OR "prediction* of threat*" OR "risk* calibration*" OR "calibration* of risk*" OR "vulnerabilit* index*" OR "index* of vulnerabilit*" OR "risk* measur*" OR "measur* of risk*" OR "vulnerability* measur*" OR "measur* of vulnerability*" OR "recidivism* measur*" OR "measur* of recidivism*" OR "dangerousness measur*" OR "measur* of dangerousness") OR AB ("need* assessment*" OR "assessment* of need*" OR "need* evaluation*" OR "evaluation* of need*" OR "need* screening*" OR "screening* of need*" OR "threat* assessment*" OR "assessment* of threat*" OR "threat* prediction*" OR "prediction* of threat*" OR "risk* calibration*" OR "calibration* of risk*" OR "vulnerabilit* index*" OR "index* of vulnerabilit*" OR "risk* measur*" OR "measur* of risk*" OR "vulnerability* measur*" OR "measur* of vulnerability*" OR "recidivism* measur*" OR "measur* of recidivism*" OR "dangerousness measur*" OR "measur* of dangerousness") OR SU ("need* assessment*" OR "assessment* of need*" OR "need* evaluation*" OR "evaluation* of need*" OR "need* screening*" OR "screening* of need*" OR "threat* assessment*" OR "assessment* of threat*" OR "threat* prediction*" OR "prediction* of threat*" OR "risk* calibration*" OR "calibration* of risk*" OR "vulnerabilit* index*" OR "index* of vulnerabilit*" OR "risk* measur*" OR "measur* of risk*" OR "vulnerability* measur*" OR "measur* of vulnerability*" OR "recidivism* measur*" OR "measur* of recidivism*" OR "dangerousness measur*" OR "measur* of dangerousness")

S3 S1 OR S2

S4 TI (radicali* OR extremis* OR fundamentalis* OR terroris* OR “hate crime*” OR "religious violence*" OR "political violence*" OR "ideological violence*" OR "environmental violence*" OR "racist violence*" OR "separatist violence*" OR "far right" OR "right wing" OR "alt right" OR "radical right" OR "extreme right" OR "white supremac*" OR "neo nazi*" OR neonazi* OR "anti semiti*" OR antisemiti* OR "left wing" OR "far left" OR "alt left" OR "anti fa" OR antifa* OR anarch* OR "anti capitalis*" OR anticapitalis* OR jihadis* OR islamis* OR salafis* OR "lone wolf*" OR "lone actor*" OR "lone offend*" OR "suicide bomb*" OR "suicide

attack*" OR "mass shoot*" OR indoctrinat* OR "foreign fight*" OR martyr*) OR AB (radicali* OR extremis* OR fundamentalis* OR terroris* OR “hate crime*” OR "religious violence*" OR "political violence*" OR "ideological violence*" OR "environmental violence*" OR "racist violence*" OR "separatist violence*" OR "far right" OR "right wing" OR "alt right" OR "radical right" OR "extreme right" OR "white supremac*" OR "neo nazi*" OR neonazi* OR "anti semiti*" OR antisemiti* OR "left wing" OR "far left" OR "alt left" OR "anti fa" OR antifa* OR anarch* OR "anti capitalis*" OR anticapitalis* OR jihadis* OR islamis* OR salafis* OR "lone wolf*" OR "lone actor*" OR "lone offend*" OR "suicide bomb*" OR "suicide attack*" OR "mass shoot*" OR indoctrinat* OR "foreign fight*" OR martyr*) OR SU (radicali* OR extremis* OR fundamentalis* OR terroris* OR “hate crime*” OR "religious violence*" OR "political violence*" OR "ideological violence*" OR "environmental violence*" OR "racist violence*" OR "separatist violence*" OR "far right" OR "right wing" OR "alt right" OR "radical right" OR "extreme right" OR "white supremac*" OR "neo nazi*" OR neonazi* OR "anti semiti*" OR antisemiti* OR "left wing" OR "far left" OR "alt left" OR "anti fa" OR antifa* OR anarch* OR "anti capitalis*" OR anticapitalis* OR jihadis* OR islamis* OR salafis* OR "lone wolf*" OR "lone actor*" OR "lone offend*" OR "suicide bomb*" OR "suicide attack*" OR "mass shoot*" OR indoctrinat* OR "foreign fight*" OR martyr*)

S5 S3 AND S4

S6 "Activism and Radicalism Intention Scales" OR "Building Resilience to Violent Extremism" OR "BRAVE 14" OR BRAVE14 OR "Extremism Monitoring Instrument" OR "EMI 20" OR EMI20 OR "Extremism Risk Guidance" OR "Extremism Risk Guidelines" OR "ERG 22+" OR ERG22+ OR "IAT 8" OR IAT8 OR "Identifying Vulnerable People Guidance" OR "Intratextual Fundamentalism Scale" OR "IR 46" OR IR46 OR "Militant Extremist Mindset" OR "Multi level Guidelines" OR "Multi-Dimensional Fundamentalism Inventory" OR "Radicalisation Risk Assessment in Prisons" OR "Référentiel des indicateurs de basculement dans la radicalisation" OR "Religious Fundamentalism Scale" OR "Significance Quest Assessment Tool" OR "Significance Quest Assessment Test" OR SyFoR OR "Terrorist Radicalization Assessment Protocol" OR "TRAP 18" OR TRAP18 OR "Violence Threat Risk Assessment" OR "Violent Extremism Beliefs Scale" OR "Violent Extremism Risk Assessment" OR "VERA 2" OR VERA2* OR "Vulnerability Assessment Framework"

S7 S5 OR S6

Filtered: Magazines (174), Newspapers (52), Book Reviews (9), Trade Publications (81)

RESULTS: 707 records (545 after duplicates)

Web of Science (Clarivate)
Search performed November 13, 2020

S1 **TOPIC:** ((risk* OR vulnerabilit* OR recidiv* OR dangerousness OR "risk factor*" OR "risk indicator*") NEAR/2 (assess* OR predict* OR evaluat* OR screening* OR tool* OR protocol* OR scale* OR instrument*)) *OR* **TOPIC:** ("need* assessment*" OR "assessment* of need*" OR "need* evaluation*" OR "evaluation* of need*" OR "need* screening*" OR "screening* of need*"

OR "threat* assessment*" OR "assessment* of threat*" OR "threat* prediction*" OR "prediction* of threat*" OR "risk* calibration*" OR "calibration* of risk*" OR "vulnerabilit* index*" OR "index* of vulnerabilit*" OR "risk* measur*" OR "measur* of risk*" OR "vulnerability* measur*" OR "measur* of vulnerability*" OR "recidivism* measur*" OR "measur* of recidivism*" OR "dangerousness measur*" OR "measur* of dangerousness")
S2 **TOPIC:** (radicali* OR extremis* OR fundamentalis* OR terroris* OR “hate crime*” OR "religious violence*" OR "political violence*" OR "ideological violence*" OR "environmental violence*" OR "racist violence*" OR "separatist violence*" OR "far right" OR "right wing" OR "alt right" OR "radical right" OR "extreme right" OR "white supremac*" OR "neo nazi*" OR neonazi* OR "anti semiti*" OR antisemiti* OR "left wing" OR "far left" OR "alt left" OR "anti fa" OR antifa* OR anarch* OR "anti capitalis*" OR anticapitalis* OR jihadis* OR islamis* OR salafis* OR "lone wolf*" OR "lone actor*" OR "lone offend*" OR "suicide bomb*" OR "suicide attack*" OR "mass shoot*" OR indoctrinat* OR "foreign fight*" OR martyr*)

S3 S1 AND S2

S4 **TOPIC:** ("Activism and Radicalism Intention Scales" OR "Building Resilience to Violent Extremism" OR "BRAVE 14" OR BRAVE14 OR "Extremism Monitoring Instrument" OR "EMI 20" OR EMI20 OR "Extremism Risk Guidance" OR "Extremism Risk Guidelines" OR "ERG 22+" OR ERG22+ OR "IAT 8" OR IAT8 OR "Identifying Vulnerable People Guidance" OR "Intratextual Fundamentalism Scale" OR "IR 46" OR IR46 OR "Militant Extremist Mindset" OR "Multi level Guidelines" OR "Multi-Dimensional Fundamentalism Inventory" OR "Radicalisation Risk Assessment in Prisons" OR "Référentiel des indicateurs de basculement dans la radicalisation" OR "Religious Fundamentalism Scale" OR "Significance Quest Assessment Tool" OR "Significance Quest Assessment Test" OR SyFoR OR "Terrorist Radicalization Assessment Protocol" OR "TRAP 18" OR TRAP18 OR "Violence Threat Risk Assessment" OR "Violent Extremism Beliefs Scale" OR "Violent Extremism Risk Assessment" OR "VERA 2" OR VERA2* OR "Vulnerability Assessment Framework")

S5 S3 OR S4

RESULTS: 1,251 (860 after duplicates)

Medline (PubMed)
Search performed November 13, 2020

#1 Search: **(risk*[Title/Abstract] OR vulnerabilit*[Title/Abstract] OR recidiv*[Title/Abstract] OR dangerousness[Title/Abstract] OR "risk factor*"[Title/Abstract] OR "risk indicator*"[Title/Abstract]) AND (assess*[Title/Abstract] OR predict*[Title/Abstract] OR evaluat*[Title/Abstract] OR screening*[Title/Abstract] OR tool*[Title/Abstract] OR protocol*[Title/Abstract] OR scale*[Title/Abstract] OR instrument*[Title/Abstract])**
("risk*"[Title/Abstract] OR "vulnerabilit*"[Title/Abstract] OR "recidiv*"[Title/Abstract] OR "dangerousness"[Title/Abstract] OR "risk factor*"[Title/Abstract] OR "risk indicator*"[Title/Abstract]) AND ("assess*"[Title/Abstract] OR "predict*"[Title/Abstract] OR

"evaluat*"[Title/Abstract] OR "screening*"[Title/Abstract] OR "tool*"[Title/Abstract] OR "protocol*"[Title/Abstract] OR "scale*"[Title/Abstract] OR "instrument*"[Title/Abstract])

#2 Search: **"need* assessment*"[Title/Abstract] OR "assessment* of need*"[Title/Abstract] OR "need* evaluation*"[Title/Abstract] OR "evaluation* of need*"[Title/Abstract] OR "need* screening*"[Title/Abstract] OR "screening* of need*"[Title/Abstract] OR "threat* assessment*"[Title/Abstract] OR "assessment* of threat*"[Title/Abstract] OR "threat* prediction*"[Title/Abstract] OR "prediction* of threat*"[Title/Abstract] OR "risk* calibration*"[Title/Abstract] OR "calibration* of risk*"[Title/Abstract] OR "vulnerabilit* index*"[Title/Abstract] OR "index* of vulnerabilit*"[Title/Abstract] OR "risk* measur*"[Title/Abstract] OR "measur* of risk*"[Title/Abstract] OR "vulnerability* measur*"[Title/Abstract] OR "measur* of vulnerability*"[Title/Abstract] OR "recidivism* measur*"[Title/Abstract] OR "measur* of recidivism*"[Title/Abstract] OR "dangerousness measur*"[Title/Abstract] OR "measur* of dangerousness"[Title/Abstract]** "need assessment*"[Title/Abstract] OR "assessment of need*"[Title/Abstract] OR "need evaluation*"[Title/Abstract] OR "need screening*"[Title/Abstract] OR "threat assessment*"[Title/Abstract] OR "threat prediction*"[Title/Abstract] OR "risk calibration*"[Title/Abstract] OR "risk measur*"[Title/Abstract] OR "vulnerability measur*"[Title/Abstract] OR "recidivism measur*"[Title/Abstract]

#3 Search: **#1 OR #2**

#4 "radicali*"[All Fields] OR "extremis*"[All Fields] OR "fundamentalis*"[All Fields] OR "terroris*"[All Fields] OR "hate crime*"[All Fields] OR "religious violence*"[All Fields] OR "political violence*"[All Fields] OR (("ideologic"[All Fields] OR "ideological"[All Fields] OR "ideologically"[All Fields]) AND "violence*"[All Fields]) OR "environmental violence*"[All Fields] OR "racist violence*"[All Fields] OR (("separatist"[All Fields] OR "separatists"[All Fields]) AND "violence*"[All Fields]) OR "far right"[All Fields] OR "right wing"[All Fields] OR "alt right"[All Fields] OR "radical right"[All Fields] OR "extreme right"[All Fields] OR "white supremac*"[All Fields] OR "neo nazi*"[All Fields] OR "neonazi*"[All Fields] OR "anti semiti*"[All Fields] OR "antisemiti*"[All Fields] OR "left wing"[All Fields] OR "far left"[All Fields] OR ("alt"[All Fields] AND "left"[All Fields]) OR "anti fa"[All Fields] OR "antifa*"[All Fields] OR "anarch*"[All Fields] OR "anti capitalis*"[All Fields] OR "anticapitalis*"[All Fields] OR "jihadis*"[All Fields] OR "islamis*"[All Fields] OR "salafis*"[All Fields] OR "lone wolf*"[All Fields] OR "lone actor*"[All Fields] OR "lone offend*"[All Fields] OR "suicide bomb*"[All Fields] OR "suicide attack*"[All Fields] OR "mass shoot*"[All Fields] OR "indoctrinat*"[All Fields] OR (("internationality"[MeSH Terms] OR "internationality"[All Fields] OR "foreign"[All Fields]) AND "fight*"[All Fields]) OR "martyr*"[All Fields]

#5 Search: **#3 AND #4**

#6 Search: "Activism[Title/Abstract] AND Radicalism Intention Scales"[Title/Abstract] OR "Building Resilience to Violent Extremism"[Title/Abstract] OR "BRAVE 14"[Title/Abstract] OR BRAVE14[Title/Abstract] OR "Extremism Monitoring Instrument"[Title/Abstract] OR "EMI 20"[Title/Abstract] OR EMI20[Title/Abstract] OR "Extremism Risk Guidance"[Title/Abstract] OR "Extremism Risk Guidelines"[Title/Abstract] OR "ERG 22+"[Title/Abstract] OR

ERG22+[Title/Abstract] OR "IAT 8"[Title/Abstract] OR IAT8[Title/Abstract] OR "Identifying Vulnerable People Guidance"[Title/Abstract] OR "Intratextual Fundamentalism Scale"[Title/Abstract] OR "IR 46"[Title/Abstract] OR IR46[Title/Abstract] OR "Militant Extremist Mindset"[Title/Abstract] OR "Multi level Guidelines"[Title/Abstract] OR "Multi-Dimensional Fundamentalism Inventory"[Title/Abstract] OR "Radicalisation Risk Assessment in Prisons"[Title/Abstract] OR "Référentiel des indicateurs de basculement dans la radicalisation"[Title/Abstract] OR "Religious Fundamentalism Scale"[Title/Abstract] OR "Significance Quest Assessment Tool"[Title/Abstract] OR "Significance Quest Assessment Test"[Title/Abstract] OR SyFoR[Title/Abstract] OR "Terrorist Radicalization Assessment Protocol"[Title/Abstract] OR "TRAP 18"[Title/Abstract] OR TRAP18[Title/Abstract] OR "Violence Threat Risk Assessment"[Title/Abstract] OR "Violent Extremism Beliefs Scale"[Title/Abstract] OR "Violent Extremism Risk Assessment"[Title/Abstract] OR "VERA 2"[Title/Abstract] OR VERA2*[Title/Abstract] OR "Vulnerability Assessment Framework"[Title/Abstract]

#7 Search: #5 OR #6

RESULTS: 1,744 (1,254 after duplicates)

ProQuest Central (ProQuest)
Search performed November 16, 2020

ab((risk* OR vulnerabilit* OR recidiv* OR dangerousness OR "risk factor*" OR "risk indicator*") NEAR/2 (assess* OR predict* OR evaluat* OR screening* OR tool* OR protocol* OR scale* OR instrument*)) AND ab(radicali* OR extremis* OR fundamentalis* OR terroris* OR "hate crime*" OR "religious violence*" OR "political violence*" OR "ideological violence*" OR "environmental violence*" OR "racist violence*" OR "separatist violence*" OR "far right" OR "right wing" OR "alt right" OR "radical right" OR "extreme right" OR "white supremac*" OR "neo nazi*" OR neonazi* OR "anti semiti*" OR antisemiti* OR "left wing" OR "far left" OR "alt left" OR "anti fa" OR antifa* OR anarch* OR "anti capitalis*" OR anticapitalis* OR jihadis* OR islamis* OR salafis* OR "lone wolf*" OR "lone actor*" OR "lone offend*" OR "suicide bomb*" OR "suicide attack*" OR "mass shoot*" OR indoctrinat* OR "foreign fight*" OR martyr*)

Filtered: Newspapers (1,304), Wire Feeds (733), Trade Journals (466), Other Sources (72), Magazines (54), Blogs (19)

RESULTS: 528 (250 after duplicates)

ProQuest Dissertations & Theses Global (ProQuest)
Search performed November 16, 2020

ab((risk* OR vulnerabilit* OR recidiv* OR dangerousness OR "risk factor*" OR "risk indicator*") NEAR/2 (assess* OR predict* OR evaluat* OR screening* OR tool* OR protocol* OR scale* OR instrument*)) AND ab(radicali* OR extremis* OR fundamentalis* OR terroris* OR "hate crime*" OR "religious violence*" OR "political violence*" OR "ideological violence*" OR "environmental violence*" OR "racist violence*" OR "separatist violence*" OR "far right" OR "right wing" OR

"alt right" OR "radical right" OR "extreme right" OR "white supremac*" OR "neo nazi*" OR neonazi* OR "anti semiti*" OR antisemiti* OR "left wing" OR "far left" OR "alt left" OR "anti fa" OR antifa* OR anarch* OR "anti capitalis*" OR anticapitalis* OR jihadis* OR islamis* OR salafis* OR "lone wolf*" OR "lone actor*" OR "lone offend*" OR "suicide bomb*" OR "suicide attack*" OR "mass shoot*" OR indoctrinat* OR "foreign fight*" OR martyr*)

RESULTS: 128 (87 after duplicates)

Sociological Abstracts (ProQuest)
Search performed November 16, 2020

((risk* OR vulnerabilit* OR recidiv* OR dangerousness OR "risk factor*" OR "risk indicator*") NEAR/2 (assess* OR predict* OR evaluat* OR screening* OR tool* OR protocol* OR scale* OR instrument*)) AND ab(radicali* OR extremis* OR fundamentalis* OR terroris* OR "hate crime*" OR "religious violence*" OR "political violence*" OR "ideological violence*" OR "environmental violence*" OR "racist violence*" OR "separatist violence*" OR "far right" OR "right wing" OR "alt right" OR "radical right" OR "extreme right" OR "white supremac*" OR "neo nazi*" OR neonazi* OR "anti semiti*" OR antisemiti* OR "left wing" OR "far left" OR "alt left" OR "anti fa" OR antifa* OR anarch* OR "anti capitalis*" OR anticapitalis* OR jihadis* OR islamis* OR salafis* OR "lone wolf*" OR "lone actor*" OR "lone offend*" OR "suicide bomb*" OR "suicide attack*" OR "mass shoot*" OR indoctrinat* OR "foreign fight*" OR martyr*)Limits applied

Databases:
Sociological Abstracts
Limited by:
Source type:4 types searched Hide list
Conference Papers & Proceedings, Dissertations & Theses, Scholarly Journals, Working Papers
Language:6 languages searched Hide list
Arabic, English, French, German, Russian, Spanish

RESULTS: 242 (167 after duplicates)

Criminal Justice Abstracts (EBSCO)
Search performed November 17, 2020

S1 TI ((risk* OR vulnerabilit* OR recidiv* OR dangerousness OR "risk factor*" OR "risk indicator*") N2 (assess* OR predict* OR evaluat* OR screening* OR tool* OR protocol* OR scale* OR instrument*)) OR AB ((risk* OR vulnerabilit* OR recidiv* OR dangerousness OR "risk factor*" OR "risk indicator*") N2 (assess* OR predict* OR evaluat* OR screening* OR tool* OR protocol* OR scale* OR instrument*)) OR SU ((risk* OR vulnerabilit* OR recidiv* OR dangerousness OR "risk factor*" OR "risk indicator*") N2 (assess* OR predict* OR evaluat* OR screening* OR tool* OR protocol* OR scale* OR instrument*))

S2 TI ("need* assessment*" OR "assessment* of need*" OR "need* evaluation*" OR "evaluation* of need*" OR "need* screening*" OR "screening* of need*" OR "threat* assessment*" OR "assessment* of threat*" OR "threat* prediction*" OR "prediction* of threat*"

OR "risk* calibration*" OR "calibration* of risk*" OR "vulnerabilit* index*" OR "index* of vulnerabilit*" OR "risk* measur*" OR "measur* of risk*" OR "vulnerability* measur*" OR "measur* of vulnerability*" OR "recidivism* measur*" OR "measur* of recidivism*" OR "dangerousness measur*" OR "measur* of dangerousness") OR AB ("need* assessment*" OR "assessment* of need*" OR "need* evaluation*" OR "evaluation* of need*" OR "need* screening*" OR "screening* of need*" OR "threat* assessment*" OR "assessment* of threat*" OR "threat* prediction*" OR "prediction* of threat*" OR "risk* calibration*" OR "calibration* of risk*" OR "vulnerabilit* index*" OR "index* of vulnerabilit*" OR "risk* measur*" OR "measur* of risk*" OR "vulnerability* measur*" OR "measur* of vulnerability*" OR "recidivism* measur*" OR "measur* of recidivism*" OR "dangerousness measur*" OR "measur* of dangerousness") OR SU ("need* assessment*" OR "assessment* of need*" OR "need* evaluation*" OR "evaluation* of need*" OR "need* screening*" OR "screening* of need*" OR "threat* assessment*" OR "assessment* of threat*" OR "threat* prediction*" OR "prediction* of threat*" OR "risk* calibration*" OR "calibration* of risk*" OR "vulnerabilit* index*" OR "index* of vulnerabilit*" OR "risk* measur*" OR "measur* of risk*" OR "vulnerability* measur*" OR "measur* of vulnerability*" OR "recidivism* measur*" OR "measur* of recidivism*" OR "dangerousness measur*" OR "measur* of dangerousness")

S3 S1 OR S2

S4 TI (radicali* OR extremis* OR fundamentalis* OR terroris* OR “hate crime*” OR "religious violence*" OR "political violence*" OR "ideological violence*" OR "environmental violence*" OR "racist violence*" OR "separatist violence*" OR "far right" OR "right wing" OR "alt right" OR "radical right" OR "extreme right" OR "white supremac*" OR "neo nazi*" OR neonazi* OR "anti semiti*" OR antisemiti* OR "left wing" OR "far left" OR "alt left" OR "anti fa" OR antifa* OR anarch* OR "anti capitalis*" OR anticapitalis* OR jihadis* OR islamis* OR salafis* OR "lone wolf*" OR "lone actor*" OR "lone offend*" OR "suicide bomb*" OR "suicide attack*" OR "mass shoot*" OR indoctrinat* OR "foreign fight*" OR martyr*) OR AB (radicali* OR extremis* OR fundamentalis* OR terroris* OR “hate crime*” OR "religious violence*" OR "political violence*" OR "ideological violence*" OR "environmental violence*" OR "racist violence*" OR "separatist violence*" OR "far right" OR "right wing" OR "alt right" OR "radical right" OR "extreme right" OR "white supremac*" OR "neo nazi*" OR neonazi* OR "anti semiti*" OR antisemiti* OR "left wing" OR "far left" OR "alt left" OR "anti fa" OR antifa* OR anarch* OR "anti capitalis*" OR anticapitalis* OR jihadis* OR islamis* OR salafis* OR "lone wolf*" OR "lone actor*" OR "lone offend*" OR "suicide bomb*" OR "suicide attack*" OR "mass shoot*" OR indoctrinat* OR "foreign fight*" OR martyr*) OR SU (radicali* OR extremis* OR fundamentalis* OR terroris* OR “hate crime*” OR "religious violence*" OR "political violence*" OR "ideological violence*" OR "environmental violence*" OR "racist violence*" OR "separatist violence*" OR "far right" OR "right wing" OR "alt right" OR "radical right" OR "extreme right" OR "white supremac*" OR "neo nazi*" OR neonazi* OR "anti semiti*" OR antisemiti* OR "left wing" OR "far left" OR "alt left" OR "anti fa" OR antifa* OR anarch* OR "anti capitalis*" OR anticapitalis* OR jihadis* OR islamis* OR salafis* OR "lone wolf*" OR "lone actor*" OR "lone offend*" OR "suicide bomb*" OR "suicide attack*" OR "mass shoot*" OR indoctrinat* OR "foreign fight*" OR martyr*)

S5 S3 AND S4

S6 "Activism and Radicalism Intention Scales" OR "Building Resilience to Violent Extremism" OR "BRAVE 14" OR BRAVE14 OR "Extremism Monitoring Instrument" OR "EMI 20" OR EMI20 OR "Extremism Risk Guidance" OR "Extremism Risk Guidelines" OR "ERG 22+" OR ERG22+ OR "IAT 8" OR IAT8 OR "Identifying Vulnerable People Guidance" OR "Intratextual Fundamentalism Scale" OR "IR 46" OR IR46 OR "Militant Extremist Mindset" OR "Multi level Guidelines" OR "Multi-Dimensional Fundamentalism Inventory" OR "Radicalisation Risk Assessment in Prisons" OR "Référentiel des indicateurs de basculement dans la radicalisation" OR "Religious Fundamentalism Scale" OR "Significance Quest Assessment Tool" OR "Significance Quest Assessment Test" OR SyFoR OR "Terrorist Radicalization Assessment Protocol" OR "TRAP 18" OR TRAP18 OR "Violence Threat Risk Assessment" OR "Violent Extremism Beliefs Scale" OR "Violent Extremism Risk Assessment" OR "VERA 2" OR VERA2* OR "Vulnerability Assessment Framework"

S7 S5 OR S6

Filtered: Magazines (38), Trade Publications (7), Reviews (4)

RESULTS = 238 (60 after duplicates)

NCJRS (Proquest)
Search performed November 17, 2020

(ti((risk* OR vulnerabilit* OR recidiv* OR dangerousness OR "risk factor*" OR "risk indicator*") NEAR/2 (assess* OR predict* OR evaluat* OR screening* OR tool* OR protocol* OR scale* OR instrument*)) OR ab((risk* OR vulnerabilit* OR recidiv* OR dangerousness OR "risk factor*" OR "risk indicator*") NEAR/2 (assess* OR predict* OR evaluat* OR screening* OR tool* OR protocol* OR scale* OR instrument*)) OR su((risk* OR vulnerabilit* OR recidiv* OR dangerousness OR "risk factor*" OR "risk indicator*") NEAR/2 (assess* OR predict* OR evaluat* OR screening* OR tool* OR protocol* OR scale* OR instrument*))) AND (radicali* OR extremis* OR fundamentalis* OR terroris* OR "hate crime*" OR "religious violence*" OR "political violence*" OR "ideological violence*" OR "environmental violence*" OR "racist violence*" OR "separatist violence*" OR "far right" OR "right wing" OR "alt right" OR "radical right" OR "extreme right" OR "white supremac*" OR "neo nazi*" OR neonazi* OR "anti semiti*" OR antisemiti* OR "left wing" OR "far left" OR "alt left" OR "anti fa" OR antifa* OR anarch* OR "anti capitalis*" OR anticapitalis* OR jihadis* OR islamis* OR salafis* OR "lone wolf*" OR "lone actor*" OR "lone offend*" OR "suicide bomb*" OR "suicide attack*" OR "mass shoot*" OR indoctrinat* OR "foreign fight*" OR martyr*)

Filtered: Blogs (1), Books (28)

RESULTS = 170 (155 after duplicates)

Violent Extremism Risk Assessment Tools Review – Search Update (Phase 2)

Overview

Source	Initial results	Results after duplicates
PsycINFO 2021	1,068	556
Academic Search Complete 2021	1,455	599
ERIC 2021	89	38
Education Source 2021	172	22
Web of Science 2021	1,415	475
ProQuest Central 2021	2,557	1,140
Sociological Abstracts 2021	438	85
ProQuest Dissertations 2021	524	255
Medline (PubMed) 2021	3,277	1,759
Criminal Justice Abstracts 2021	392	34
NCJRS (ProQuest) 2021	768	572
**TOTAL**	**12,155**	**5,535**

PsycINFO (APA PsycNET)
Search performed August 4, 2022
NOTE: search updated until end of 2021, new strategy run so no start date set.

1,068 Results for (((Any Field: ("Activism and Radicalism Intention Scales") OR Any Field: ("Building Resilience to Violent Extremism") OR Any Field: ("BRAVE 14") OR Any Field: (BRAVE14) OR Any Field: ("Extremism Monitoring Instrument") OR Any Field: ("EMI 20") OR Any Field: (EMI20) OR Any Field: ("Extremism Risk Guidance") OR Any Field: ("Extremism Risk Guidelines") OR Any Field: ("ERG 22+") OR Any Field: (ERG22+) OR Any Field: ("IAT 8") OR Any Field: (IAT8) OR Any Field: ("Identifying Vulnerable People Guidance") OR Any Field: ("Intratextual Fundamentalism Scale") OR Any Field: ("IR 46") OR Any Field: (IR46) OR Any Field: ("Militant Extremist Mindset") OR Any Field: ("Multi level Guidelines") OR Any Field: ("Multi-Dimensional Fundamentalism Inventory") OR Any Field: ("Radicalisation Risk Assessment in Prisons") OR Any Field: ("Référentiel des indicateurs de basculement dans la radicalisation") OR Any Field: ("Religious Fundamentalism Scale") OR Any Field: ("Significance Quest Assessment Tool") OR Any Field: ("Significance Quest Assessment Test") OR Any Field: (SyFoR) OR Any Field: ("Terrorist Radicalization Assessment Protocol") OR Any Field: ("TRAP 18") OR Any Field: (TRAP18) OR Any Field: ("Violence Threat Risk Assessment") OR Any Field: ("Violent Extremism Beliefs Scale") OR Any Field: ("Violent Extremism Risk Assessment") OR Any Field: ("VERA 2") OR Any Field: (VERA2*) OR Any Field: ("Vulnerability Assessment Framework"))) AND ((Year: [0 TO 2021]))) OR (((((title: (radicali*)) OR (title: (extremis*)) OR (title: (fundamentalis*)) OR (title: (terroris*)) OR (title: ("hate crime*")) OR (title: ("religious violence*")) OR (title: ("political violence*")) OR (title: ("ideological violence*")) OR (title: ("environmental violence*")) OR (title: ("racist violence*")) OR (title: ("separatist violence*")) OR (title: ("far right")) OR (title: ("right wing")) OR (title: ("alt right")) OR (title: ("radical right")) OR (title: ("extreme right")) OR (title: ("white supremac*")) OR (title: ("neo nazi*")) OR (title: (neonazi*)) OR (title: ("anti

semiti*")) OR (title: (antisemiti*)) OR (title: ("anti-semiti*")) OR (title: ("left wing")) OR (title: ("far left")) OR (title: ("alt left")) OR (title: ("anti fa")) OR (title: (antifa*)) OR (title: (anarch*)) OR (title: ("anti capitalis*")) OR (title: (anticapitalis*)) OR (title: (jihadis*)) OR (title: (islamis*)) OR (title: (salafis*)) OR (title: ("lone wolf*")) OR (title: ("lone actor*")) OR (title: ("lone offend*")) OR (title: ("suicide bomb*")) OR (title: ("suicide attack*")) OR (title: ("mass shoot*")) OR (title: (indoctrinat*)) OR (title: ("foreign fight*")) OR (title: (martyr*))) OR ((abstract: (radicali*)) OR (abstract: (extremis*)) OR (abstract: (fundamentalis*)) OR (abstract: (terroris*)) OR (abstract: ("hate crime*")) OR (abstract: ("religious violence*")) OR (abstract: ("political violence*")) OR (abstract: ("ideological violence*")) OR (abstract: ("environmental violence*")) OR (abstract: ("racist violence*")) OR (abstract: ("separatist violence*")) OR (abstract: ("far right")) OR (abstract: ("right wing")) OR (abstract: ("alt right")) OR (abstract: ("radical right")) OR (abstract: ("extreme right")) OR (abstract: ("white supremac*")) OR (abstract: ("neo nazi*")) OR (abstract: (neonazi*)) OR (abstract: ("anti semiti*")) OR (abstract: (antisemiti*)) OR (abstract: ("anti-semiti*")) OR (abstract: ("left wing")) OR (abstract: ("far left")) OR (abstract: ("alt left")) OR (abstract: ("anti fa")) OR (abstract: (antifa*)) OR (abstract: (anarch*)) OR (abstract: ("anti capitalis*")) OR (abstract: (anticapitalis*)) OR (abstract: (jihadis*)) OR (abstract: (islamis*)) OR (abstract: (salafis*)) OR (abstract: ("lone wolf*")) OR (abstract: ("lone actor*")) OR (abstract: ("lone offend*")) OR (abstract: ("suicide bomb*")) OR (abstract: ("suicide attack*")) OR (abstract: ("mass shoot*")) OR (abstract: (indoctrinat*)) OR (abstract: ("foreign fight*")) OR (abstract: (martyr*))) OR ((Index Terms: (radicali*)) OR (Index Terms: (extremis*)) OR (Index Terms: (fundamentalis*)) OR (Index Terms: (terroris*)) OR (Index Terms: ("hate crime*")) OR (Index Terms: ("religious violence*")) OR (Index Terms: ("political violence*")) OR (Index Terms: ("ideological violence*")) OR (Index Terms: ("environmental violence*")) OR (Index Terms: ("racist violence*")) OR (Index Terms: ("separatist violence*")) OR (Index Terms: ("far right")) OR (Index Terms: ("right wing")) OR (Index Terms: ("alt right")) OR (Index Terms: ("radical right")) OR (Index Terms: ("extreme right")) OR (Index Terms: ("white supremac*")) OR (Index Terms: ("neo nazi*")) OR (Index Terms: (neonazi*)) OR (Index Terms: ("anti semiti*")) OR (Index Terms: (antisemiti*)) OR (Index Terms: ("anti-semiti*")) OR (Index Terms: ("left wing")) OR (Index Terms: ("far left")) OR (Index Terms: ("alt left")) OR (Index Terms: ("anti fa")) OR (Index Terms: (antifa*)) OR (Index Terms: (anarch*)) OR (Index Terms: ("anti capitalis*")) OR (Index Terms: (anticapitalis*)) OR (Index Terms: (jihadis*)) OR (Index Terms: (islamis*)) OR (Index Terms: (salafis*)) OR (Index Terms: ("lone wolf*")) OR (Index Terms: ("lone actor*")) OR (Index Terms: ("lone offend*")) OR (Index Terms: ("suicide bomb*")) OR (Index Terms: ("suicide attack*")) OR (Index Terms: ("mass shoot*")) OR (Index Terms: (indoctrinat*)) OR (Index Terms: ("foreign fight*")) OR (Index Terms: (martyr*))) OR ((Keywords: (radicali*)) OR (Keywords: (extremis*)) OR (Keywords: (fundamentalis*)) OR (Keywords: (terroris*)) OR (Keywords: ("hate crime*")) OR (Keywords: ("religious violence*")) OR (Keywords: ("political violence*")) OR (Keywords: ("ideological violence*")) OR (Keywords: ("environmental violence*")) OR (Keywords: ("racist violence*")) OR (Keywords: ("separatist violence*")) OR (Keywords: ("far right")) OR (Keywords: ("right wing")) OR (Keywords: ("alt right")) OR (Keywords: ("radical right")) OR (Keywords: ("extreme right")) OR (Keywords: ("white supremac*")) OR (Keywords: ("neo nazi*")) OR (Keywords: (neonazi*)) OR (Keywords: ("anti semiti*")) OR (Keywords: (antisemiti*)) OR (Keywords: ("anti-semiti*")) OR (Keywords: ("left wing")) OR (Keywords: ("far left")) OR (Keywords: ("alt left")) OR (Keywords: ("anti fa")) OR (Keywords: (antifa*)) OR (Keywords: (anarch*)) OR (Keywords: ("anti capitalis*")) OR

(Keywords: (anticapitalis*)) OR (Keywords: (jihadis*)) OR (Keywords: (islamis*)) OR (Keywords: (salafis*)) OR (Keywords: ("lone wolf*")) OR (Keywords: ("lone actor*")) OR (Keywords: ("lone offend*")) OR (Keywords: ("suicide bomb*")) OR (Keywords: ("suicide attack*")) OR (Keywords: ("mass shoot*")) OR (Keywords: (indoctrinat*)) OR (Keywords: ("foreign fight*")) OR (Keywords: (martyr*))) OR ((MeSH: (radicali*)) OR (MeSH: (extremis*)) OR (MeSH: (fundamentalis*)) OR (MeSH: (terroris*)) OR (MeSH: ("hate crime*")) OR (MeSH: ("religious violence*")) OR (MeSH: ("political violence*")) OR (MeSH: ("ideological violence*")) OR (MeSH: ("environmental violence*")) OR (MeSH: ("racist violence*")) OR (MeSH: ("separatist violence*")) OR (MeSH: ("far right")) OR (MeSH: ("right wing")) OR (MeSH: ("alt right")) OR (MeSH: ("radical right")) OR (MeSH: ("extreme right")) OR (MeSH: ("white supremac*")) OR (MeSH: ("neo nazi*")) OR (MeSH: (neonazi*)) OR (MeSH: ("anti semiti*")) OR (MeSH: (antisemiti*)) OR (MeSH: ("anti-semiti*")) OR (MeSH: ("left wing")) OR (MeSH: ("far left")) OR (MeSH: ("alt left")) OR (MeSH: ("anti fa")) OR (MeSH: (antifa*)) OR (MeSH: (anarch*)) OR (MeSH: ("anti capitalis*")) OR (MeSH: (anticapitalis*)) OR (MeSH: (jihadis*)) OR (MeSH: (islamis*)) OR (MeSH: (salafis*)) OR (MeSH: ("lone wolf*")) OR (MeSH: ("lone actor*")) OR (MeSH: ("lone offend*")) OR (MeSH: ("suicide bomb*")) OR (MeSH: ("suicide attack*")) OR (MeSH: ("mass shoot*")) OR (MeSH: (indoctrinat*)) OR (MeSH: ("foreign fight*")) OR (MeSH: (martyr*)))) AND ((Year: [0 TO 2021]))) AND ((((title: (risk*)) OR (title: (vulnerabil*)) OR (title: (recidiv*)) OR (title: (dangerous*)) OR (title: (threat*)) OR (title: (need*))) NEAR/3 ((title: (assess*)) OR (title: (predict*)) OR (title: (evaluat*)) OR (title: (screen*)) OR (title: (tool*)) OR (title: (protocol*)) OR (title: (scale*)) OR (title: (instrument*)) OR (title: (calibrat*)) OR (title: (index*)) OR (title: (measur*))) OR ((abstract: (risk*)) OR (abstract: (vulnerabil*)) OR (abstract: (recidiv*)) OR (abstract: (dangerous*)) OR (abstract: (threat*)) OR (abstract: (need*))) NEAR/3 ((abstract: (assess*)) OR (abstract: (predict*)) OR (abstract: (evaluat*)) OR (abstract: (screen*)) OR (abstract: (tool*)) OR (abstract: (protocol*)) OR (abstract: (scale*)) OR (abstract: (instrument*)) OR (abstract: (calibrat*)) OR (abstract: (index*)) OR (abstract: (measur*))) OR ((Index Terms: (risk*)) OR (Index Terms: (vulnerabil*)) OR (Index Terms: (recidiv*)) OR (Index Terms: (dangerous*)) OR (Index Terms: (threat*)) OR (Index Terms: (need*))) NEAR/3 ((Index Terms: (assess*)) OR (Index Terms: (predict*)) OR (Index Terms: (evaluat*)) OR (Index Terms: (screen*)) OR (Index Terms: (tool*)) OR (Index Terms: (protocol*)) OR (Index Terms: (scale*)) OR (Index Terms: (instrument*)) OR (Index Terms: (calibrat*)) OR (Index Terms: (index*)) OR (Index Terms: (measur*))) OR ((Keywords: (risk*)) OR (Keywords: (vulnerabil*)) OR (Keywords: (recidiv*)) OR (Keywords: (dangerous*)) OR (Keywords: (threat*)) OR (Keywords: (need*))) NEAR/3 ((Keywords: (assess*)) OR (Keywords: (predict*)) OR (Keywords: (evaluat*)) OR (Keywords: (screen*)) OR (Keywords: (tool*)) OR (Keywords: (protocol*)) OR (Keywords: (scale*)) OR (Keywords: (instrument*)) OR (Keywords: (calibrat*)) OR (Keywords: (index*)) OR (Keywords: (measur*))) OR ((MeSH: (risk*)) OR (MeSH: (vulnerabil*)) OR (MeSH: (recidiv*)) OR (MeSH: (dangerous*)) OR (MeSH: (threat*)) OR (MeSH: (need*))) NEAR/3 ((MeSH: (assess*)) OR (MeSH: (predict*)) OR (MeSH: (evaluat*)) OR (MeSH: (screen*)) OR (MeSH: (tool*)) OR (MeSH: (protocol*)) OR (MeSH: (scale*)) OR (MeSH: (instrument*)) OR (MeSH: (calibrat*)) OR (MeSH: (index*)) OR (MeSH: (measur*)))) AND ((Year: [0 TO 2021]))))

RESULTS: 1,068 (556 after duplicates)

Academic Search Complete (EBSCO)
Search performed August 4, 2022
NOTE: search updated until end of 2021, new strategy run so no start date set.

S1: TI ((risk* or vulnerabil* or recidiv* or dangerous* or threat* or need*) N3 (assess* or predict* or evaluat* or screen* or tool* or protocol* or scale* or instrument* or calibrat* or index* or measur*)) OR SU ((risk* or vulnerabil* or recidiv* or dangerous* or threat* or need*) N3 (assess* or predict* or evaluat* or screen* or tool* or protocol* or scale* or instrument* or calibrat* or index* or measur*)) OR AB ((risk* or vulnerabil* or recidiv* or dangerous* or threat* or need*) N3 (assess* or predict* or evaluat* or screen* or tool* or protocol* or scale* or instrument* or calibrat* or index* or measur*)) OR KW ((risk* or vulnerabil* or recidiv* or dangerous* or threat* or need*) N3 (assess* or predict* or evaluat* or screen* or tool* or protocol* or scale* or instrument* or calibrat* or index* or measur*))

S2: TI (radicali* or extremis* or fundamentalis* or terroris* or "hate crime*" or "religious violence*" or "political violence*" or "ideological violence*" or "environmental violence*" or "racist violence*" or "separatist violence*" or "far right" or "right wing" or "alt right" or "radical right" or "extreme right" or "white supremac*" or "neo nazi*" or neonazi* or "anti semiti*" or antisemiti* or "anti-semiti*" or "left wing" or "far left" or "alt left" or "anti fa" or antifa* or anarch* or "anti capitalis*" or anticapitalis* or jihadis* or islamis* or salafis* or "lone wolf*" or "lone actor*" or "lone offend*" or "suicide bomb*" or "suicide attack*" or "mass shoot*" or indoctrinat* or "foreign fight*" or martyr*) OR SU (radicali* or extremis* or fundamentalis* or terroris* or "hate crime*" or "religious violence*" or "political violence*" or "ideological violence*" or "environmental violence*" or "racist violence*" or "separatist violence*" or "far right" or "right wing" or "alt right" or "radical right" or "extreme right" or "white supremac*" or "neo nazi*" or neonazi* or "anti semiti*" or antisemiti* or "anti-semiti*" or "left wing" or "far left" or "alt left" or "anti fa" or antifa* or anarch* or "anti capitalis*" or anticapitalis* or jihadis* or islamis* or salafis* or "lone wolf*" or "lone actor*" or "lone offend*" or "suicide bomb*" or "suicide attack*" or "mass shoot*" or indoctrinat* or "foreign fight*" or martyr*) OR AB (radicali* or extremis* or fundamentalis* or terroris* or "hate crime*" or "religious violence*" or "political violence*" or "ideological violence*" or "environmental violence*" or "racist violence*" or "separatist violence*" or "far right" or "right wing" or "alt right" or "radical right" or "extreme right" or "white supremac*" or "neo nazi*" or neonazi* or "anti semiti*" or antisemiti* or "anti-semiti*" or "left wing" or "far left" or "alt left" or "anti fa" or antifa* or anarch* or "anti capitalis*" or anticapitalis* or jihadis* or islamis* or salafis* or "lone wolf*" or "lone actor*" or "lone offend*" or "suicide bomb*" or "suicide attack*" or "mass shoot*" or indoctrinat* or "foreign fight*" or martyr*) OR KW (radicali* or extremis* or fundamentalis* or terroris* or "hate crime*" or "religious violence*" or "political violence*" or "ideological violence*" or "environmental violence*" or "racist violence*" or "separatist violence*" or "far right" or "right wing" or "alt right" or "radical right" or "extreme right" or "white supremac*" or "neo nazi*" or neonazi* or "anti semiti*" or antisemiti* or "anti-semiti*" or "left wing" or "far left" or "alt left" or "anti fa" or antifa* or anarch* or "anti capitalis*" or anticapitalis* or jihadis* or islamis* or salafis* or "lone wolf*" or "lone actor*" or "lone offend*" or "suicide bomb*" or "suicide attack*" or "mass shoot*" or indoctrinat* or "foreign fight*" or martyr*)

S3: S1 AND S2

S4: TI ("Activism and Radicalism Intention Scales" or "Building Resilience to Violent Extremism" or "BRAVE 14" or BRAVE14 or "Extremism Monitoring Instrument" or "EMI 20" or EMI20 or "Extremism Risk Guidance" or "Extremism Risk Guidelines" or "ERG 22+" or ERG22+ or "IAT 8" or IAT8 or "Identifying Vulnerable People Guidance" or "Intratextual Fundamentalism Scale" or "IR 46" or IR46 or "Militant Extremist Mindset" or "Multi level Guidelines" or "Multi-Dimensional Fundamentalism Inventory" or "Radicalisation Risk Assessment in Prisons" or "Référentiel des indicateurs de basculement dans la radicalisation" or "Religious Fundamentalism Scale" or "Significance Quest Assessment Tool" or "Significance Quest Assessment Test" or SyFoR or "Terrorist Radicalization Assessment Protocol" or "TRAP 18" or TRAP18 or "Violence Threat Risk Assessment" or "Violent Extremism Beliefs Scale" or "Violent Extremism Risk Assessment" or "VERA 2" or VERA2* or "Vulnerability Assessment Framework") OR SU ("Activism and Radicalism Intention Scales" or "Building Resilience to Violent Extremism" or "BRAVE 14" or BRAVE14 or "Extremism Monitoring Instrument" or "EMI 20" or EMI20 or "Extremism Risk Guidance" or "Extremism Risk Guidelines" or "ERG 22+" or ERG22+ or "IAT 8" or IAT8 or "Identifying Vulnerable People Guidance" or "Intratextual Fundamentalism Scale" or "IR 46" or IR46 or "Militant Extremist Mindset" or "Multi level Guidelines" or "Multi-Dimensional Fundamentalism Inventory" or "Radicalisation Risk Assessment in Prisons" or "Référentiel des indicateurs de basculement dans la radicalisation" or "Religious Fundamentalism Scale" or "Significance Quest Assessment Tool" or "Significance Quest Assessment Test" or SyFoR or "Terrorist Radicalization Assessment Protocol" or "TRAP 18" or TRAP18 or "Violence Threat Risk Assessment" or "Violent Extremism Beliefs Scale" or "Violent Extremism Risk Assessment" or "VERA 2" or VERA2* or "Vulnerability Assessment Framework") OR AB ("Activism and Radicalism Intention Scales" or "Building Resilience to Violent Extremism" or "BRAVE 14" or BRAVE14 or "Extremism Monitoring Instrument" or "EMI 20" or EMI20 or "Extremism Risk Guidance" or "Extremism Risk Guidelines" or "ERG 22+" or ERG22+ or "IAT 8" or IAT8 or "Identifying Vulnerable People Guidance" or "Intratextual Fundamentalism Scale" or "IR 46" or IR46 or "Militant Extremist Mindset" or "Multi level Guidelines" or "Multi-Dimensional Fundamentalism Inventory" or "Radicalisation Risk Assessment in Prisons" or "Référentiel des indicateurs de basculement dans la radicalisation" or "Religious Fundamentalism Scale" or "Significance Quest Assessment Tool" or "Significance Quest Assessment Test" or SyFoR or "Terrorist Radicalization Assessment Protocol" or "TRAP 18" or TRAP18 or "Violence Threat Risk Assessment" or "Violent Extremism Beliefs Scale" or "Violent Extremism Risk Assessment" or "VERA 2" or VERA2* or "Vulnerability Assessment Framework") OR KW ("Activism and Radicalism Intention Scales" or "Building Resilience to Violent Extremism" or "BRAVE 14" or BRAVE14 or "Extremism Monitoring Instrument" or "EMI 20" or EMI20 or "Extremism Risk Guidance" or "Extremism Risk Guidelines" or "ERG 22+" or ERG22+ or "IAT 8" or IAT8 or "Identifying Vulnerable People Guidance" or "Intratextual Fundamentalism Scale" or "IR 46" or IR46 or "Militant Extremist Mindset" or "Multi level Guidelines" or "Multi-Dimensional Fundamentalism Inventory" or "Radicalisation Risk Assessment in Prisons" or "Référentiel des indicateurs de basculement dans la radicalisation" or "Religious Fundamentalism Scale" or "Significance Quest Assessment Tool" or "Significance Quest Assessment Test" or SyFoR or "Terrorist Radicalization Assessment Protocol" or "TRAP 18" or TRAP18 or "Violence Threat Risk Assessment" or "Violent Extremism Beliefs Scale" or "Violent Extremism Risk Assessment" or "VERA 2" or VERA2* or "Vulnerability Assessment Framework")

S5: S3 OR S4

Limiters - Published Date: -20211231
Filtered: Magazines (295), Newspapers (141), Book Reviews (17)

RESULTS: 1,455 (599 after duplicates)

ERIC (EBSCO)
Search performed August 4, 2022
NOTE: search updated until end of 2021, new strategy run so no start date set.

S1: TI ((risk* or vulnerabil* or recidiv* or dangerous* or threat* or need*) N3 (assess* or predict* or evaluat* or screen* or tool* or protocol* or scale* or instrument* or calibrat* or index* or measur*)) OR SU ((risk* or vulnerabil* or recidiv* or dangerous* or threat* or need*) N3 (assess* or predict* or evaluat* or screen* or tool* or protocol* or scale* or instrument* or calibrat* or index* or measur*)) OR AB ((risk* or vulnerabil* or recidiv* or dangerous* or threat* or need*) N3 (assess* or predict* or evaluat* or screen* or tool* or protocol* or scale* or instrument* or calibrat* or index* or measur*)) OR KW ((risk* or vulnerabil* or recidiv* or dangerous* or threat* or need*) N3 (assess* or predict* or evaluat* or screen* or tool* or protocol* or scale* or instrument* or calibrat* or index* or measur*))

S2: TI (radicali* or extremis* or fundamentalis* or terroris* or "hate crime*" or "religious violence*" or "political violence*" or "ideological violence*" or "environmental violence*" or "racist violence*" or "separatist violence*" or "far right" or "right wing" or "alt right" or "radical right" or "extreme right" or "white supremac*" or "neo nazi*" or neonazi* or "anti semiti*" or antisemiti* or "anti-semiti*" or "left wing" or "far left" or "alt left" or "anti fa" or antifa* or anarch* or "anti capitalis*" or anticapitalis* or jihadis* or islamis* or salafis* or "lone wolf*" or "lone actor*" or "lone offend*" or "suicide bomb*" or "suicide attack*" or "mass shoot*" or indoctrinat* or "foreign fight*" or martyr*) OR SU (radicali* or extremis* or fundamentalis* or terroris* or "hate crime*" or "religious violence*" or "political violence*" or "ideological violence*" or "environmental violence*" or "racist violence*" or "separatist violence*" or "far right" or "right wing" or "alt right" or "radical right" or "extreme right" or "white supremac*" or "neo nazi*" or neonazi* or "anti semiti*" or antisemiti* or "anti-semiti*" or "left wing" or "far left" or "alt left" or "anti fa" or antifa* or anarch* or "anti capitalis*" or anticapitalis* or jihadis* or islamis* or salafis* or "lone wolf*" or "lone actor*" or "lone offend*" or "suicide bomb*" or "suicide attack*" or "mass shoot*" or indoctrinat* or "foreign fight*" or martyr*) OR AB (radicali* or extremis* or fundamentalis* or terroris* or "hate crime*" or "religious violence*" or "political violence*" or "ideological violence*" or "environmental violence*" or "racist violence*" or "separatist violence*" or "far right" or "right wing" or "alt right" or "radical right" or "extreme right" or "white supremac*" or "neo nazi*" or neonazi* or "anti semiti*" or antisemiti* or "anti-semiti*" or "left wing" or "far left" or "alt left" or "anti fa" or antifa* or anarch* or "anti capitalis*" or anticapitalis* or jihadis* or islamis* or salafis* or "lone wolf*" or "lone actor*" or "lone offend*" or "suicide bomb*" or "suicide attack*" or "mass shoot*" or indoctrinat* or "foreign fight*" or martyr*) OR KW (radicali* or extremis* or fundamentalis* or terroris* or "hate crime*" or "religious violence*" or "political violence*" or "ideological violence*" or "environmental violence*" or "racist violence*" or "separatist violence*" or "far

right" or "right wing" or "alt right" or "radical right" or "extreme right" or "white supremac*" or "neo nazi*" or neonazi* or "anti semiti*" or antisemiti* or "anti-semiti*" or "left wing" or "far left" or "alt left" or "anti fa" or antifa* or anarch* or "anti capitalis*" or anticapitalis* or jihadis* or islamis* or salafis* or "lone wolf*" or "lone actor*" or "lone offend*" or "suicide bomb*" or "suicide attack*" or "mass shoot*" or indoctrinat* or "foreign fight*" or martyr*)

S3: S1 AND S2

S4: TI ("Activism and Radicalism Intention Scales" or "Building Resilience to Violent Extremism" or "BRAVE 14" or BRAVE14 or "Extremism Monitoring Instrument" or "EMI 20" or EMI20 or "Extremism Risk Guidance" or "Extremism Risk Guidelines" or "ERG 22+" or ERG22+ or "IAT 8" or IAT8 or "Identifying Vulnerable People Guidance" or "Intratextual Fundamentalism Scale" or "IR 46" or IR46 or "Militant Extremist Mindset" or "Multi level Guidelines" or "Multi-Dimensional Fundamentalism Inventory" or "Radicalisation Risk Assessment in Prisons" or "Référentiel des indicateurs de basculement dans la radicalisation" or "Religious Fundamentalism Scale" or "Significance Quest Assessment Tool" or "Significance Quest Assessment Test" or SyFoR or "Terrorist Radicalization Assessment Protocol" or "TRAP 18" or TRAP18 or "Violence Threat Risk Assessment" or "Violent Extremism Beliefs Scale" or "Violent Extremism Risk Assessment" or "VERA 2" or VERA2* or "Vulnerability Assessment Framework") OR SU ("Activism and Radicalism Intention Scales" or "Building Resilience to Violent Extremism" or "BRAVE 14" or BRAVE14 or "Extremism Monitoring Instrument" or "EMI 20" or EMI20 or "Extremism Risk Guidance" or "Extremism Risk Guidelines" or "ERG 22+" or ERG22+ or "IAT 8" or IAT8 or "Identifying Vulnerable People Guidance" or "Intratextual Fundamentalism Scale" or "IR 46" or IR46 or "Militant Extremist Mindset" or "Multi level Guidelines" or "Multi-Dimensional Fundamentalism Inventory" or "Radicalisation Risk Assessment in Prisons" or "Référentiel des indicateurs de basculement dans la radicalisation" or "Religious Fundamentalism Scale" or "Significance Quest Assessment Tool" or "Significance Quest Assessment Test" or SyFoR or "Terrorist Radicalization Assessment Protocol" or "TRAP 18" or TRAP18 or "Violence Threat Risk Assessment" or "Violent Extremism Beliefs Scale" or "Violent Extremism Risk Assessment" or "VERA 2" or VERA2* or "Vulnerability Assessment Framework") OR AB ("Activism and Radicalism Intention Scales" or "Building Resilience to Violent Extremism" or "BRAVE 14" or BRAVE14 or "Extremism Monitoring Instrument" or "EMI 20" or EMI20 or "Extremism Risk Guidance" or "Extremism Risk Guidelines" or "ERG 22+" or ERG22+ or "IAT 8" or IAT8 or "Identifying Vulnerable People Guidance" or "Intratextual Fundamentalism Scale" or "IR 46" or IR46 or "Militant Extremist Mindset" or "Multi level Guidelines" or "Multi-Dimensional Fundamentalism Inventory" or "Radicalisation Risk Assessment in Prisons" or "Référentiel des indicateurs de basculement dans la radicalisation" or "Religious Fundamentalism Scale" or "Significance Quest Assessment Tool" or "Significance Quest Assessment Test" or SyFoR or "Terrorist Radicalization Assessment Protocol" or "TRAP 18" or TRAP18 or "Violence Threat Risk Assessment" or "Violent Extremism Beliefs Scale" or "Violent Extremism Risk Assessment" or "VERA 2" or VERA2* or "Vulnerability Assessment Framework") OR KW ("Activism and Radicalism Intention Scales" or "Building Resilience to Violent Extremism" or "BRAVE 14" or BRAVE14 or "Extremism Monitoring Instrument" or "EMI 20" or EMI20 or "Extremism Risk Guidance" or "Extremism Risk Guidelines" or "ERG 22+" or ERG22+ or "IAT 8" or IAT8 or "Identifying Vulnerable People Guidance" or "Intratextual Fundamentalism Scale" or "IR 46" or

IR46 or "Militant Extremist Mindset" or "Multi level Guidelines" or "Multi-Dimensional Fundamentalism Inventory" or "Radicalisation Risk Assessment in Prisons" or "Référentiel des indicateurs de basculement dans la radicalisation" or "Religious Fundamentalism Scale" or "Significance Quest Assessment Tool" or "Significance Quest Assessment Test" or SyFoR or "Terrorist Radicalization Assessment Protocol" or "TRAP 18" or TRAP18 or "Violence Threat Risk Assessment" or "Violent Extremism Beliefs Scale" or "Violent Extremism Risk Assessment" or "VERA 2" or VERA2* or "Vulnerability Assessment Framework")

S5: S3 OR S4

Limiters - Published Date: -20211231
Filtered: Electronic Resources (12)

RESULTS: 89 (38 after duplicates)

Education Source (EBSCO)
Search performed August 4, 2022, 2022
NOTE: search updated until end of 2021, new strategy run so no start date set.

S1: TI ((risk* or vulnerabil* or recidiv* or dangerous* or threat* or need*) N3 (assess* or predict* or evaluat* or screen* or tool* or protocol* or scale* or instrument* or calibrat* or index* or measur*)) OR SU ((risk* or vulnerabil* or recidiv* or dangerous* or threat* or need*) N3 (assess* or predict* or evaluat* or screen* or tool* or protocol* or scale* or instrument* or calibrat* or index* or measur*)) OR AB ((risk* or vulnerabil* or recidiv* or dangerous* or threat* or need*) N3 (assess* or predict* or evaluat* or screen* or tool* or protocol* or scale* or instrument* or calibrat* or index* or measur*)) OR KW ((risk* or vulnerabil* or recidiv* or dangerous* or threat* or need*) N3 (assess* or predict* or evaluat* or screen* or tool* or protocol* or scale* or instrument* or calibrat* or index* or measur*))

S2: TI (radicali* or extremis* or fundamentalis* or terroris* or "hate crime*" or "religious violence*" or "political violence*" or "ideological violence*" or "environmental violence*" or "racist violence*" or "separatist violence*" or "far right" or "right wing" or "alt right" or "radical right" or "extreme right" or "white supremac*" or "neo nazi*" or neonazi* or "anti semiti*" or antisemiti* or "anti-semiti*" or "left wing" or "far left" or "alt left" or "anti fa" or antifa* or anarch* or "anti capitalis*" or anticapitalis* or jihadis* or islamis* or salafis* or "lone wolf*" or "lone actor*" or "lone offend*" or "suicide bomb*" or "suicide attack*" or "mass shoot*" or indoctrinat* or "foreign fight*" or martyr*) OR SU (radicali* or extremis* or fundamentalis* or terroris* or "hate crime*" or "religious violence*" or "political violence*" or "ideological violence*" or "environmental violence*" or "racist violence*" or "separatist violence*" or "far right" or "right wing" or "alt right" or "radical right" or "extreme right" or "white supremac*" or "neo nazi*" or neonazi* or "anti semiti*" or antisemiti* or "anti-semiti*" or "left wing" or "far left" or "alt left" or "anti fa" or antifa* or anarch* or "anti capitalis*" or anticapitalis* or jihadis* or islamis* or salafis* or "lone wolf*" or "lone actor*" or "lone offend*" or "suicide bomb*" or "suicide attack*" or "mass shoot*" or indoctrinat* or "foreign fight*" or martyr*) OR AB (radicali* or extremis* or fundamentalis* or terroris* or "hate crime*" or "religious violence*" or "political violence*" or "ideological violence*" or "environmental violence*" or "racist

violence*" or "separatist violence*" or "far right" or "right wing" or "alt right" or "radical right" or "extreme right" or "white supremac*" or "neo nazi*" or neonazi* or "anti semiti*" or antisemiti* or "anti-semiti*" or "left wing" or "far left" or "alt left" or "anti fa" or antifa* or anarch* or "anti capitalis*" or anticapitalis* or jihadis* or islamis* or salafis* or "lone wolf*" or "lone actor*" or "lone offend*" or "suicide bomb*" or "suicide attack*" or "mass shoot*" or indoctrinat* or "foreign fight*" or martyr*) OR KW (radicali* or extremis* or fundamentalis* or terroris* or "hate crime*" or "religious violence*" or "political violence*" or "ideological violence*" or "environmental violence*" or "racist violence*" or "separatist violence*" or "far right" or "right wing" or "alt right" or "radical right" or "extreme right" or "white supremac*" or "neo nazi*" or neonazi* or "anti semiti*" or antisemiti* or "anti-semiti*" or "left wing" or "far left" or "alt left" or "anti fa" or antifa* or anarch* or "anti capitalis*" or anticapitalis* or jihadis* or islamis* or salafis* or "lone wolf*" or "lone actor*" or "lone offend*" or "suicide bomb*" or "suicide attack*" or "mass shoot*" or indoctrinat* or "foreign fight*" or martyr*)

S3: S1 AND S2

S4: TI ("Activism and Radicalism Intention Scales" or "Building Resilience to Violent Extremism" or "BRAVE 14" or BRAVE14 or "Extremism Monitoring Instrument" or "EMI 20" or EMI20 or "Extremism Risk Guidance" or "Extremism Risk Guidelines" or "ERG 22+" or ERG22+ or "IAT 8" or IAT8 or "Identifying Vulnerable People Guidance" or "Intratextual Fundamentalism Scale" or "IR 46" or IR46 or "Militant Extremist Mindset" or "Multi level Guidelines" or "Multi-Dimensional Fundamentalism Inventory" or "Radicalisation Risk Assessment in Prisons" or "Référentiel des indicateurs de basculement dans la radicalisation" or "Religious Fundamentalism Scale" or "Significance Quest Assessment Tool" or "Significance Quest Assessment Test" or SyFoR or "Terrorist Radicalization Assessment Protocol" or "TRAP 18" or TRAP18 or "Violence Threat Risk Assessment" or "Violent Extremism Beliefs Scale" or "Violent Extremism Risk Assessment" or "VERA 2" or VERA2* or "Vulnerability Assessment Framework") OR SU ("Activism and Radicalism Intention Scales" or "Building Resilience to Violent Extremism" or "BRAVE 14" or BRAVE14 or "Extremism Monitoring Instrument" or "EMI 20" or EMI20 or "Extremism Risk Guidance" or "Extremism Risk Guidelines" or "ERG 22+" or ERG22+ or "IAT 8" or IAT8 or "Identifying Vulnerable People Guidance" or "Intratextual Fundamentalism Scale" or "IR 46" or IR46 or "Militant Extremist Mindset" or "Multi level Guidelines" or "Multi-Dimensional Fundamentalism Inventory" or "Radicalisation Risk Assessment in Prisons" or "Référentiel des indicateurs de basculement dans la radicalisation" or "Religious Fundamentalism Scale" or "Significance Quest Assessment Tool" or "Significance Quest Assessment Test" or SyFoR or "Terrorist Radicalization Assessment Protocol" or "TRAP 18" or TRAP18 or "Violence Threat Risk Assessment" or "Violent Extremism Beliefs Scale" or "Violent Extremism Risk Assessment" or "VERA 2" or VERA2* or "Vulnerability Assessment Framework") OR AB ("Activism and Radicalism Intention Scales" or "Building Resilience to Violent Extremism" or "BRAVE 14" or BRAVE14 or "Extremism Monitoring Instrument" or "EMI 20" or EMI20 or "Extremism Risk Guidance" or "Extremism Risk Guidelines" or "ERG 22+" or ERG22+ or "IAT 8" or IAT8 or "Identifying Vulnerable People Guidance" or "Intratextual Fundamentalism Scale" or "IR 46" or IR46 or "Militant Extremist Mindset" or "Multi level Guidelines" or "Multi-Dimensional Fundamentalism Inventory" or "Radicalisation Risk Assessment in Prisons" or "Référentiel des indicateurs de basculement dans la radicalisation" or "Religious Fundamentalism Scale" or "Significance Quest

Assessment Tool" or "Significance Quest Assessment Test" or SyFoR or "Terrorist Radicalization Assessment Protocol" or "TRAP 18" or TRAP18 or "Violence Threat Risk Assessment" or "Violent Extremism Beliefs Scale" or "Violent Extremism Risk Assessment" or "VERA 2" or VERA2* or "Vulnerability Assessment Framework") OR KW ("Activism and Radicalism Intention Scales" or "Building Resilience to Violent Extremism" or "BRAVE 14" or BRAVE14 or "Extremism Monitoring Instrument" or "EMI 20" or EMI20 or "Extremism Risk Guidance" or "Extremism Risk Guidelines" or "ERG 22+" or ERG22+ or "IAT 8" or IAT8 or "Identifying Vulnerable People Guidance" or "Intratextual Fundamentalism Scale" or "IR 46" or IR46 or "Militant Extremist Mindset" or "Multi level Guidelines" or "Multi-Dimensional Fundamentalism Inventory" or "Radicalisation Risk Assessment in Prisons" or "Référentiel des indicateurs de basculement dans la radicalisation" or "Religious Fundamentalism Scale" or "Significance Quest Assessment Tool" or "Significance Quest Assessment Test" or SyFoR or "Terrorist Radicalization Assessment Protocol" or "TRAP 18" or TRAP18 or "Violence Threat Risk Assessment" or "Violent Extremism Beliefs Scale" or "Violent Extremism Risk Assessment" or "VERA 2" or VERA2* or "Vulnerability Assessment Framework")

S5: S3 OR S4

Limiters - Published Date: -20211231
Filtered: Magazines (53), Newspapers (9), Book Reviews (2)

RESULTS: 172 (22 after duplicates)

Web of Science (Clarivate)
Search performed August 4, 2022, 2022
NOTE: search updated until end of 2021, new strategy run so no start date set.

(risk* OR vulnerabilit* OR recidiv* OR dangerous* OR threat* OR need*) NEAR/3 (assess* OR predict* OR evaluat* OR screen* OR tool* OR protocol* OR scale* OR instrument* OR calibrat* OR index* OR measur*) (Topic) and radicali* OR extremis* OR fundamentalis* OR terroris* OR "hate crime*" OR "religious violence*" OR "political violence*" OR "ideological violence*" OR "environmental violence*" OR "racist violence*" OR "separatist violence*" OR "far right" OR "right wing" OR "alt right" OR "radical right" OR "extreme right" OR "white supremac*" OR "neo nazi*" OR neonazi* OR "anti semiti*" OR antisemiti* OR "anti-semiti*" OR "left wing" OR "far left" OR "alt left" OR "anti fa" OR antifa* OR anarch* OR "anti capitalis*" OR anticapitalis* OR jihadis* OR islamis* OR salafis* OR "lone wolf*" OR "lone actor*" OR "lone offend*" OR "suicide bomb*" OR "suicide attack*" OR "mass shoot*" OR indoctrinat* OR "foreign fight*" OR martyr* (Topic) OR "Activism and Radicalism Intention Scales" or "Building Resilience to Violent Extremism" or "BRAVE 14" or BRAVE14 or "Extremism Monitoring Instrument" or "EMI 20" or EMI20 or "Extremism Risk Guidance" or "Extremism Risk Guidelines" or "ERG 22+" or ERG22+ or "IAT 8" or IAT8 or "Identifying Vulnerable People Guidance" or "Intratextual Fundamentalism Scale" or "IR 46" or IR46 or "Militant Extremist Mindset" or "Multi level Guidelines" or "Multi-Dimensional Fundamentalism Inventory" or "Radicalisation Risk Assessment in Prisons" or "Référentiel des indicateurs de basculement dans la radicalisation" or "Religious Fundamentalism Scale" or "Significance Quest Assessment Tool" or "Significance Quest Assessment Test" or SyFoR or

"Terrorist Radicalization Assessment Protocol" or "TRAP 18" or TRAP18 or "Violence Threat Risk Assessment" or "Violent Extremism Beliefs Scale" or "Violent Extremism Risk Assessment" or "VERA 2" or VERA2* or "Vulnerability Assessment Framework" (Topic)

11:27 AM | Timespan: 1900-01-01 to 2021-12-31 (Publication Date)
Editions = A&HCI , ESCI , CPCI-SSH , SSCI

RESULTS: 1,415 (475 after duplicates)

<u>ProQuest Central (ProQuest)</u>
Search performed August 4, 2022, 2022
NOTE: search updated until end of 2021, new strategy run so no start date set.

Proquest Central includes the following databases:

- ABI/INFORM Collection (1971 - current)
- Arts & Humanities Database
- Asian & European Business Collection (1971 - current)
- Australia & New Zealand Database
- Business Market Research Collection (1986 - current)
- Canadian Business & Current Affairs Database
- Canadian Newsstream
- Career & Technical Education Database
- Consumer Health Database
- Continental Europe Database
- Criminal Justice Database (1981 - current)
- East & South Asia Database
- East Europe, Central Europe Database
- Education Database (1988 - current)
- Health & Medical Collection
- Healthcare Administration Database
- India Database (1998 - current)
- Latin America & Iberia Database
- Materials Science Database
- Middle East & Africa Database
- Military Database
- Nursing & Allied Health Database
- Political Science Database (1985 - current)
- Psychology Database
- Public Health Database
- Publicly Available Content Database
- Religion Database (1986 - current)
- Research Library
- Social Science Database
- Sociology Database (1985 - current)
- Telecommunications Database (1995 - current)

- Turkey Database information
- UK & Ireland Database

((noft((risk* OR vulnerabilit* OR recidiv* OR dangerous* OR threat* OR need*) NEAR/3 (assess* OR predict* OR evaluat* OR screen* OR tool* OR protocol* OR scale* OR instrument* OR calibrat* OR index* OR measur*)) AND noft(radicali* OR extremis* OR fundamentalis* OR terroris* OR "hate crime*" OR "religious violence*" OR "political violence*" OR "ideological violence*" OR "environmental violence*" OR "racist violence*" OR "separatist violence*" OR "far right" OR "right wing" OR "alt right" OR "radical right" OR "extreme right" OR "white supremac*" OR "neo nazi*" OR neonazi* OR "anti semiti*" OR antisemiti* OR "anti-semiti*" OR "left wing" OR "far left" OR "alt left" OR "anti fa" OR antifa* OR anarch* OR "anti capitalis*" OR anticapitalis* OR jihadis* OR islamis* OR salafis* OR "lone wolf*" OR "lone actor*" OR "lone offend*" OR "suicide bomb*" OR "suicide attack*" OR "mass shoot*" OR indoctrinat* OR "foreign fight*" OR martyr*)) NOT stype.exact("Newspapers" OR "Wire Feeds" OR "Trade Journals" OR "Other Sources" OR "Magazines" OR "Blogs, Podcasts, & Websites")) OR noft("Activism and Radicalism Intention Scales" OR "Building Resilience to Violent Extremism" OR "BRAVE 14" OR BRAVE14 OR "Extremism Monitoring Instrument" OR "EMI 20" OR EMI20 OR "Extremism Risk Guidance" OR "Extremism Risk Guidelines" OR "ERG 22+" OR ERG22+ OR "IAT 8" OR IAT8 OR "Identifying Vulnerable People Guidance" OR "Intratextual Fundamentalism Scale" OR "IR 46" OR IR46 OR "Militant Extremist Mindset" OR "Multi level Guidelines" OR "Multi-Dimensional Fundamentalism Inventory" OR "Radicalisation Risk Assessment in Prisons" OR "Référentiel des indicateurs de basculement dans la radicalisation" OR "Religious Fundamentalism Scale" OR "Significance Quest Assessment Tool" OR "Significance Quest Assessment Test" OR SyFoR OR "Terrorist Radicalization Assessment Protocol" OR "TRAP 18" OR TRAP18 OR "Violence Threat Risk Assessment" OR "Violent Extremism Beliefs Scale" OR "Violent Extremism Risk Assessment" OR "VERA 2" OR VERA2* OR "Vulnerability Assessment Framework")

Limited by:
Date: Before 31 December 2021
Excluded: Newspapers, Magazines, Wire Feeds, Trade Journals, Blogs, Other Sources

RESULTS: 2,557 (1,140 after duplicates)

Sociological Abstracts (ProQuest) – includes Social Services Abstracts)
Search performed August 4, 2022
NOTE: search updated until end of 2021, new strategy run so no start date set.

((noft((risk* OR vulnerabilit* OR recidiv* OR dangerous* OR threat* OR need*) NEAR/3 (assess* OR predict* OR evaluat* OR screen* OR tool* OR protocol* OR scale* OR instrument* OR calibrat* OR index* OR measur*)) AND noft(radicali* OR extremis* OR fundamentalis* OR terroris* OR "hate crime*" OR "religious violence*" OR "political violence*" OR "ideological violence*" OR "environmental violence*" OR "racist violence*" OR "separatist violence*" OR "far right" OR "right wing" OR "alt right" OR "radical right" OR "extreme right" OR "white supremac*" OR "neo nazi*" OR neonazi* OR "anti semiti*" OR

antisemiti* OR "anti-semiti*" OR "left wing" OR "far left" OR "alt left" OR "anti fa" OR antifa* OR anarch* OR "anti capitalis*" OR anticapitalis* OR jihadis* OR islamis* OR salafis* OR "lone wolf*" OR "lone actor*" OR "lone offend*" OR "suicide bomb*" OR "suicide attack*" OR "mass shoot*" OR indoctrinat* OR "foreign fight*" OR martyr*)) NOT stype.exact("Newspapers" OR "Wire Feeds" OR "Trade Journals" OR "Other Sources" OR "Magazines" OR "Blogs, Podcasts, & Websites")) OR noft("Activism and Radicalism Intention Scales" OR "Building Resilience to Violent Extremism" OR "BRAVE 14" OR BRAVE14 OR "Extremism Monitoring Instrument" OR "EMI 20" OR EMI20 OR "Extremism Risk Guidance" OR "Extremism Risk Guidelines" OR "ERG 22+" OR ERG22+ OR "IAT 8" OR IAT8 OR "Identifying Vulnerable People Guidance" OR "Intratextual Fundamentalism Scale" OR "IR 46" OR IR46 OR "Militant Extremist Mindset" OR "Multi level Guidelines" OR "Multi-Dimensional Fundamentalism Inventory" OR "Radicalisation Risk Assessment in Prisons" OR "Référentiel des indicateurs de basculement dans la radicalisation" OR "Religious Fundamentalism Scale" OR "Significance Quest Assessment Tool" OR "Significance Quest Assessment Test" OR SyFoR OR "Terrorist Radicalization Assessment Protocol" OR "TRAP 18" OR TRAP18 OR "Violence Threat Risk Assessment" OR "Violent Extremism Beliefs Scale" OR "Violent Extremism Risk Assessment" OR "VERA 2" OR VERA2* OR "Vulnerability Assessment Framework")

Limited by:
Date: Before 31 December 2021
Excluded: Magazines

RESULTS: 438 (85 after duplicates)

Dissertations & Theses Global (ProQuest)
Search performed August 4, 2022
NOTE: search updated until end of 2021, new strategy run so no start date set.

((noft((risk* OR vulnerabilit* OR recidiv* OR dangerous* OR threat* OR need*) NEAR/3 (assess* OR predict* OR evaluat* OR screen* OR tool* OR protocol* OR scale* OR instrument* OR calibrat* OR index* OR measur*)) AND noft(radicali* OR extremis* OR fundamentalis* OR terroris* OR "hate crime*" OR "religious violence*" OR "political violence*" OR "ideological violence*" OR "environmental violence*" OR "racist violence*" OR "separatist violence*" OR "far right" OR "right wing" OR "alt right" OR "radical right" OR "extreme right" OR "white supremac*" OR "neo nazi*" OR neonazi* OR "anti semiti*" OR antisemiti* OR "anti-semiti*" OR "left wing" OR "far left" OR "alt left" OR "anti fa" OR antifa* OR anarch* OR "anti capitalis*" OR anticapitalis* OR jihadis* OR islamis* OR salafis* OR "lone wolf*" OR "lone actor*" OR "lone offend*" OR "suicide bomb*" OR "suicide attack*" OR "mass shoot*" OR indoctrinat* OR "foreign fight*" OR martyr*)) NOT stype.exact("Newspapers" OR "Wire Feeds" OR "Trade Journals" OR "Other Sources" OR "Magazines" OR "Blogs, Podcasts, & Websites")) OR noft("Activism and Radicalism Intention Scales" OR "Building Resilience to Violent Extremism" OR "BRAVE 14" OR BRAVE14 OR "Extremism Monitoring Instrument" OR "EMI 20" OR EMI20 OR "Extremism Risk Guidance" OR "Extremism Risk Guidelines" OR "ERG 22+" OR ERG22+ OR "IAT 8" OR IAT8 OR "Identifying Vulnerable People Guidance" OR "Intratextual Fundamentalism Scale" OR "IR 46"

OR IR46 OR "Militant Extremist Mindset" OR "Multi level Guidelines" OR "Multi-Dimensional Fundamentalism Inventory" OR "Radicalisation Risk Assessment in Prisons" OR "Référentiel des indicateurs de basculement dans la radicalisation" OR "Religious Fundamentalism Scale" OR "Significance Quest Assessment Tool" OR "Significance Quest Assessment Test" OR SyFoR OR "Terrorist Radicalization Assessment Protocol" OR "TRAP 18" OR TRAP18 OR "Violence Threat Risk Assessment" OR "Violent Extremism Beliefs Scale" OR "Violent Extremism Risk Assessment" OR "VERA 2" OR VERA2* OR "Vulnerability Assessment Framework")

Limited by:
Date: Before 31 December 2021

RESULTS: 524 (255 after duplicates)

Medline (PubMed)
Search performed August 9, 2022
NOTE: search updated until end of 2021, new strategy run so no start date set.

Search: (((assess*[Title/Abstract] OR predict*[Title/Abstract] OR evaluat*[Title/Abstract] OR screen*[Title/Abstract] OR tool*[Title/Abstract] OR protocol*[Title/Abstract] OR scale*[Title/Abstract] OR instrument*[Title/Abstract] OR calibrat*[Title/Abstract] OR index*[Title/Abstract] OR measur*[Title/Abstract]) AND (risk*[Title/Abstract] OR vulnerabil*[Title/Abstract] OR recidiv*[Title/Abstract] OR dangerous*[Title/Abstract] OR threat*[Title/Abstract] OR need*[Title/Abstract])) AND (radicali*[Title/Abstract] OR extremis*[Title/Abstract] OR fundamentalis*[Title/Abstract] OR terroris*[Title/Abstract] OR "hate crime*"[Title/Abstract] OR "religious violence*"[Title/Abstract] OR "political violence*"[Title/Abstract] OR "ideological violence*"[Title/Abstract] OR "environmental violence*"[Title/Abstract] OR "racist violence*"[Title/Abstract] OR "separatist violence*"[Title/Abstract] OR "far right"[Title/Abstract] OR "right wing"[Title/Abstract] OR "alt right"[Title/Abstract] OR "radical right"[Title/Abstract] OR "extreme right"[Title/Abstract] OR "white supremac*"[Title/Abstract] OR "neo nazi*"[Title/Abstract] OR neonazi*[Title/Abstract] OR "anti semiti*"[Title/Abstract] OR antisemiti*[Title/Abstract] OR "anti-semiti*"[Title/Abstract] OR "left wing"[Title/Abstract] OR "far left"[Title/Abstract] OR "alt left"[Title/Abstract] OR "anti fa"[Title/Abstract] OR antifa*[Title/Abstract] OR anarch*[Title/Abstract] OR "anti capitalis*"[Title/Abstract] OR anticapitalis*[Title/Abstract] OR jihadis*[Title/Abstract] OR islamis*[Title/Abstract] OR salafis*[Title/Abstract] OR "lone wolf*"[Title/Abstract] OR "lone actor*"[Title/Abstract] OR "lone offend*"[Title/Abstract] OR "suicide bomb*"[Title/Abstract] OR "suicide attack*"[Title/Abstract] OR "mass shoot*"[Title/Abstract] OR indoctrinat*[Title/Abstract] OR "foreign fight*"[Title/Abstract] OR martyr*[Title/Abstract]) AND (fha[Filter])) OR ("Activism and Radicalism Intention Scales"[Title/Abstract] OR "Building Resilience to Violent Extremism"[Title/Abstract] OR "BRAVE 14"[Title/Abstract] OR BRAVE14[Title/Abstract] OR "Extremism Monitoring Instrument"[Title/Abstract] OR "EMI 20"[Title/Abstract] OR EMI20[Title/Abstract] OR "Extremism Risk Guidance"[Title/Abstract] OR "Extremism Risk Guidelines"[Title/Abstract] OR "ERG 22+"[Title/Abstract] OR ERG22+[Title/Abstract] OR "IAT 8"[Title/Abstract] OR IAT8[Title/Abstract] OR "Identifying Vulnerable People Guidance"[Title/Abstract] OR

"Intratextual Fundamentalism Scale"[Title/Abstract] OR "IR 46"[Title/Abstract] OR IR46[Title/Abstract] OR "Militant Extremist Mindset"[Title/Abstract] OR "Multi level Guidelines"[Title/Abstract] OR "Multi-Dimensional Fundamentalism Inventory"[Title/Abstract] OR "Radicalisation Risk Assessment in Prisons"[Title/Abstract] OR "Référentiel des indicateurs de basculement dans la radicalisation"[Title/Abstract] OR "Religious Fundamentalism Scale"[Title/Abstract] OR "Significance Quest Assessment Tool"[Title/Abstract] OR "Significance Quest Assessment Test"[Title/Abstract] OR SyFoR[Title/Abstract] OR "Terrorist Radicalization Assessment Protocol"[Title/Abstract] OR "TRAP 18"[Title/Abstract] OR TRAP18[Title/Abstract] OR "Violence Threat Risk Assessment"[Title/Abstract] OR "Violent Extremism Beliefs Scale"[Title/Abstract] OR "Violent Extremism Risk Assessment"[Title/Abstract] OR "VERA 2"[Title/Abstract] OR VERA2*[Title/Abstract] OR "Vulnerability Assessment Framework"[Title/Abstract] AND (fha[Filter])) Filters: Abstract Filters: Abstract, from 1974 - 2021

RESULTS: 3,277 (1,759 after duplicates)

Criminal Justice Abstracts (EBSCO)
Search performed August 9, 2022
NOTE: search updated until end of 2021, new strategy run so no start date set.

S1: TI ((risk* or vulnerabil* or recidiv* or dangerous* or threat* or need*) N3 (assess* or predict* or evaluat* or screen* or tool* or protocol* or scale* or instrument* or calibrat* or index* or measur*)) OR AB ((risk* or vulnerabil* or recidiv* or dangerous* or threat* or need*) N3 (assess* or predict* or evaluat* or screen* or tool* or protocol* or scale* or instrument* or calibrat* or index* or measur*)) OR KW ((risk* or vulnerabil* or recidiv* or dangerous* or threat* or need*) N3 (assess* or predict* or evaluat* or screen* or tool* or protocol* or scale* or instrument* or calibrat* or index* or measur*)) OR SU ((risk* or vulnerabil* or recidiv* or dangerous* or threat* or need*) N3 (assess* or predict* or evaluat* or screen* or tool* or protocol* or scale* or instrument* or calibrat* or index* or measur*))

S2: TI (radicali* or extremis* or fundamentalis* or terroris* or "hate crime*" or "religious violence*" or "political violence*" or "ideological violence*" or "environmental violence*" or "racist violence*" or "separatist violence*" or "far right" or "right wing" or "alt right" or "radical right" or "extreme right" or "white supremac*" or "neo nazi*" or neonazi* or "anti semiti*" or antisemiti* or "anti-semiti*" or "left wing" or "far left" or "alt left" or "anti fa" or antifa* or anarch* or "anti capitalis*" or anticapitalis* or jihadis* or islamis* or salafis* or "lone wolf*" or "lone actor*" or "lone offend*" or "suicide bomb*" or "suicide attack*" or "mass shoot*" or indoctrinat* or "foreign fight*" or martyr*) OR AB (radicali* or extremis* or fundamentalis* or terroris* or "hate crime*" or "religious violence*" or "political violence*" or "ideological violence*" or "environmental violence*" or "racist violence*" or "separatist violence*" or "far right" or "right wing" or "alt right" or "radical right" or "extreme right" or "white supremac*" or "neo nazi*" or neonazi* or "anti semiti*" or antisemiti* or "anti-semiti*" or "left wing" or "far left" or "alt left" or "anti fa" or antifa* or anarch* or "anti capitalis*" or anticapitalis* or jihadis* or islamis* or salafis* or "lone wolf*" or "lone actor*" or "lone offend*" or "suicide bomb*" or "suicide attack*" or "mass shoot*" or indoctrinat* or "foreign fight*" or martyr*) OR KW (radicali* or extremis* or fundamentalis* or terroris* or "hate crime*" or "religious violence*" or

"political violence*" or "ideological violence*" or "environmental violence*" or "racist violence*" or "separatist violence*" or "far right" or "right wing" or "alt right" or "radical right" or "extreme right" or "white supremac*" or "neo nazi*" or neonazi* or "anti semiti*" or antisemiti* or "anti-semiti*" or "left wing" or "far left" or "alt left" or "anti fa" or antifa* or anarch* or "anti capitalis*" or anticapitalis* or jihadis* or islamis* or salafis* or "lone wolf*" or "lone actor*" or "lone offend*" or "suicide bomb*" or "suicide attack*" or "mass shoot*" or indoctrinat* or "foreign fight*" or martyr*) OR SU (radicali* or extremis* or fundamentalis* or terroris* or "hate crime*" or "religious violence*" or "political violence*" or "ideological violence*" or "environmental violence*" or "racist violence*" or "separatist violence*" or "far right" or "right wing" or "alt right" or "radical right" or "extreme right" or "white supremac*" or "neo nazi*" or neonazi* or "anti semiti*" or antisemiti* or "anti-semiti*" or "left wing" or "far left" or "alt left" or "anti fa" or antifa* or anarch* or "anti capitalis*" or anticapitalis* or jihadis* or islamis* or salafis* or "lone wolf*" or "lone actor*" or "lone offend*" or "suicide bomb*" or "suicide attack*" or "mass shoot*" or indoctrinat* or "foreign fight*" or martyr*)

S3: S1 AND S2

S4: TI ("Activism and Radicalism Intention Scales" or "Building Resilience to Violent Extremism" or "BRAVE 14" or BRAVE14 or "Extremism Monitoring Instrument" or "EMI 20" or EMI20 or "Extremism Risk Guidance" or "Extremism Risk Guidelines" or "ERG 22+" or ERG22+ or "IAT 8" or IAT8 or "Identifying Vulnerable People Guidance" or "Intratextual Fundamentalism Scale" or "IR 46" or IR46 or "Militant Extremist Mindset" or "Multi level Guidelines" or "Multi-Dimensional Fundamentalism Inventory" or "Radicalisation Risk Assessment in Prisons" or "Référentiel des indicateurs de basculement dans la radicalisation" or "Religious Fundamentalism Scale" or "Significance Quest Assessment Tool" or "Significance Quest Assessment Test" or SyFoR or "Terrorist Radicalization Assessment Protocol" or "TRAP 18" or TRAP18 or "Violence Threat Risk Assessment" or "Violent Extremism Beliefs Scale" or "Violent Extremism Risk Assessment" or "VERA 2" or VERA2* or "Vulnerability Assessment Framework") OR SU ("Activism and Radicalism Intention Scales" or "Building Resilience to Violent Extremism" or "BRAVE 14" or BRAVE14 or "Extremism Monitoring Instrument" or "EMI 20" or EMI20 or "Extremism Risk Guidance" or "Extremism Risk Guidelines" or "ERG 22+" or ERG22+ or "IAT 8" or IAT8 or "Identifying Vulnerable People Guidance" or "Intratextual Fundamentalism Scale" or "IR 46" or IR46 or "Militant Extremist Mindset" or "Multi level Guidelines" or "Multi-Dimensional Fundamentalism Inventory" or "Radicalisation Risk Assessment in Prisons" or "Référentiel des indicateurs de basculement dans la radicalisation" or "Religious Fundamentalism Scale" or "Significance Quest Assessment Tool" or "Significance Quest Assessment Test" or SyFoR or "Terrorist Radicalization Assessment Protocol" or "TRAP 18" or TRAP18 or "Violence Threat Risk Assessment" or "Violent Extremism Beliefs Scale" or "Violent Extremism Risk Assessment" or "VERA 2" or VERA2* or "Vulnerability Assessment Framework") OR AB ("Activism and Radicalism Intention Scales" or "Building Resilience to Violent Extremism" or "BRAVE 14" or BRAVE14 or "Extremism Monitoring Instrument" or "EMI 20" or EMI20 or "Extremism Risk Guidance" or "Extremism Risk Guidelines" or "ERG 22+" or ERG22+ or "IAT 8" or IAT8 or "Identifying Vulnerable People Guidance" or "Intratextual Fundamentalism Scale" or "IR 46" or IR46 or "Militant Extremist Mindset" or "Multi level Guidelines" or "Multi-Dimensional Fundamentalism Inventory" or "Radicalisation Risk Assessment in Prisons" or "Référentiel des indicateurs de

basculement dans la radicalisation" or "Religious Fundamentalism Scale" or "Significance Quest Assessment Tool" or "Significance Quest Assessment Test" or SyFoR or "Terrorist Radicalization Assessment Protocol" or "TRAP 18" or TRAP18 or "Violence Threat Risk Assessment" or "Violent Extremism Beliefs Scale" or "Violent Extremism Risk Assessment" or "VERA 2" or VERA2* or "Vulnerability Assessment Framework") OR KW ("Activism and Radicalism Intention Scales" or "Building Resilience to Violent Extremism" or "BRAVE 14" or BRAVE14 or "Extremism Monitoring Instrument" or "EMI 20" or EMI20 or "Extremism Risk Guidance" or "Extremism Risk Guidelines" or "ERG 22+" or ERG22+ or "IAT 8" or IAT8 or "Identifying Vulnerable People Guidance" or "Intratextual Fundamentalism Scale" or "IR 46" or IR46 or "Militant Extremist Mindset" or "Multi level Guidelines" or "Multi-Dimensional Fundamentalism Inventory" or "Radicalisation Risk Assessment in Prisons" or "Référentiel des indicateurs de basculement dans la radicalisation" or "Religious Fundamentalism Scale" or "Significance Quest Assessment Tool" or "Significance Quest Assessment Test" or SyFoR or "Terrorist Radicalization Assessment Protocol" or "TRAP 18" or TRAP18 or "Violence Threat Risk Assessment" or "Violent Extremism Beliefs Scale" or "Violent Extremism Risk Assessment" or "VERA 2" or VERA2* or "Vulnerability Assessment Framework") OR AB ("Activism and Radicalism Intention Scales" or "Building Resilience to Violent Extremism" or "BRAVE 14" or BRAVE14 or "Extremism Monitoring Instrument" or "EMI 20" or EMI20 or "Extremism Risk Guidance" or "Extremism Risk Guidelines" or "ERG 22+" or ERG22+ or "IAT 8" or IAT8 or "Identifying Vulnerable People Guidance" or "Intratextual Fundamentalism Scale" or "IR 46" or IR46 or "Militant Extremist Mindset" or "Multi level Guidelines" or "Multi-Dimensional Fundamentalism Inventory" or "Radicalisation Risk Assessment in Prisons" or "Référentiel des indicateurs de basculement dans la radicalisation" or "Religious Fundamentalism Scale" or "Significance Quest Assessment Tool" or "Significance Quest Assessment Test" or SyFoR or "Terrorist Radicalization Assessment Protocol" or "TRAP 18" or TRAP18 or "Violence Threat Risk Assessment" or "Violent Extremism Beliefs Scale" or "Violent Extremism Risk Assessment" or "VERA 2" or VERA2* or "Vulnerability Assessment Framework") OR SU ("Activism and Radicalism Intention Scales" or "Building Resilience to Violent Extremism" or "BRAVE 14" or BRAVE14 or "Extremism Monitoring Instrument" or "EMI 20" or EMI20 or "Extremism Risk Guidance" or "Extremism Risk Guidelines" or "ERG 22+" or ERG22+ or "IAT 8" or IAT8 or "Identifying Vulnerable People Guidance" or "Intratextual Fundamentalism Scale" or "IR 46" or IR46 or "Militant Extremist Mindset" or "Multi level Guidelines" or "Multi-Dimensional Fundamentalism Inventory" or "Radicalisation Risk Assessment in Prisons" or "Référentiel des indicateurs de basculement dans la radicalisation" or "Religious Fundamentalism Scale" or "Significance Quest Assessment Tool" or "Significance Quest Assessment Test" or SyFoR or "Terrorist Radicalization Assessment Protocol" or "TRAP 18" or TRAP18 or "Violence Threat Risk Assessment" or "Violent Extremism Beliefs Scale" or "Violent Extremism Risk Assessment" or "VERA 2" or VERA2* or "Vulnerability Assessment Framework") OR AB ("Activism and Radicalism Intention Scales" or "Building Resilience to Violent Extremism" or "BRAVE 14" or BRAVE14 or "Extremism Monitoring Instrument" or "EMI 20" or EMI20 or "Extremism Risk Guidance" or "Extremism Risk Guidelines" or "ERG 22+" or ERG22+ or "IAT 8" or IAT8 or "Identifying Vulnerable People Guidance" or "Intratextual Fundamentalism Scale" or "IR 46" or IR46 or "Militant Extremist Mindset" or "Multi level Guidelines" or "Multi-Dimensional Fundamentalism Inventory" or "Radicalisation Risk Assessment in Prisons" or "Référentiel des indicateurs de basculement dans la radicalisation" or "Religious Fundamentalism Scale" or "Significance Quest

Assessment Tool" or "Significance Quest Assessment Test" or SyFoR or "Terrorist Radicalization Assessment Protocol" or "TRAP 18" or TRAP18 or "Violence Threat Risk Assessment" or "Violent Extremism Beliefs Scale" or "Violent Extremism Risk Assessment" or "VERA 2" or VERA2* or "Vulnerability Assessment Framework") OR KW ("Activism and Radicalism Intention Scales" or "Building Resilience to Violent Extremism" or "BRAVE 14" or BRAVE14 or "Extremism Monitoring Instrument" or "EMI 20" or EMI20 or "Extremism Risk Guidance" or "Extremism Risk Guidelines" or "ERG 22+" or ERG22+ or "IAT 8" or IAT8 or "Identifying Vulnerable People Guidance" or "Intratextual Fundamentalism Scale" or "IR 46" or IR46 or "Militant Extremist Mindset" or "Multi level Guidelines" or "Multi-Dimensional Fundamentalism Inventory" or "Radicalisation Risk Assessment in Prisons" or "Référentiel des indicateurs de basculement dans la radicalisation" or "Religious Fundamentalism Scale" or "Significance Quest Assessment Tool" or "Significance Quest Assessment Test" or SyFoR or "Terrorist Radicalization Assessment Protocol" or "TRAP 18" or TRAP18 or "Violence Threat Risk Assessment" or "Violent Extremism Beliefs Scale" or "Violent Extremism Risk Assessment" or "VERA 2" or VERA2* or "Vulnerability Assessment Framework") OR AB ("Activism and Radicalism Intention Scales" or "Building Resilience to Violent Extremism" or "BRAVE 14" or BRAVE14 or "Extremism Monitoring Instrument" or "EMI 20" or EMI20 or "Extremism Risk Guidance" or "Extremism Risk Guidelines" or "ERG 22+" or ERG22+ or "IAT 8" or IAT8 or "Identifying Vulnerable People Guidance" or "Intratextual Fundamentalism Scale" or "IR 46" or IR46 or "Militant Extremist Mindset" or "Multi level Guidelines" or "Multi-Dimensional Fundamentalism Inventory" or "Radicalisation Risk Assessment in Prisons" or "Référentiel des indicateurs de basculement dans la radicalisation" or "Religious Fundamentalism Scale" or "Significance Quest Assessment Tool" or "Significance Quest Assessment Test" or SyFoR or "Terrorist Radicalization Assessment Protocol" or "TRAP 18" or TRAP18 or "Violence Threat Risk Assessment" or "Violent Extremism Beliefs Scale" or "Violent Extremism Risk Assessment" or "VERA 2" or VERA2* or "Vulnerability Assessment Framework") OR SU ("Activism and Radicalism Intention Scales" or "Building Resilience to Violent Extremism" or "BRAVE 14" or BRAVE14 or "Extremism Monitoring Instrument" or "EMI 20" or EMI20 or "Extremism Risk Guidance" or "Extremism Risk Guidelines" or "ERG 22+" or ERG22+ or "IAT 8" or IAT8 or "Identifying Vulnerable People Guidance" or "Intratextual Fundamentalism Scale" or "IR 46" or IR46 or "Militant Extremist Mindset" or "Multi level Guidelines" or "Multi-Dimensional Fundamentalism Inventory" or "Radicalisation Risk Assessment in Prisons" or "Référentiel des indicateurs de basculement dans la radicalisation" or "Religious Fundamentalism Scale" or "Significance Quest Assessment Tool" or "Significance Quest Assessment Test" or SyFoR or "Terrorist Radicalization Assessment Protocol" or "TRAP 18" or TRAP18 or "Violence Threat Risk Assessment" or "Violent Extremism Beliefs Scale" or "Violent Extremism Risk Assessment" or "VERA 2" or VERA2* or "Vulnerability Assessment Framework") OR AB ("Activism and Radicalism Intention Scales" or "Building Resilience to Violent Extremism" or "BRAVE 14" or BRAVE14 or "Extremism Monitoring Instrument" or "EMI 20" or EMI20 or "Extremism Risk Guidance" or "Extremism Risk Guidelines" or "ERG 22+" or ERG22+ or "IAT 8" or IAT8 or "Identifying Vulnerable People Guidance" or "Intratextual Fundamentalism Scale" or "IR 46" or IR46 or "Militant Extremist Mindset" or "Multi level Guidelines" or "Multi-Dimensional Fundamentalism Inventory" or "Radicalisation Risk Assessment in Prisons" or "Référentiel des indicateurs de basculement dans la radicalisation" or "Religious Fundamentalism Scale" or "Significance Quest Assessment Tool" or "Significance Quest Assessment Test" or SyFoR or "Terrorist

Radicalization Assessment Protocol" or "TRAP 18" or TRAP18 or "Violence Threat Risk Assessment" or "Violent Extremism Beliefs Scale" or "Violent Extremism Risk Assessment" or "VERA 2" or VERA2* or "Vulnerability Assessment Framework") OR KW ("Activism and Radicalism Intention Scales" or "Building Resilience to Violent Extremism" or "BRAVE 14" or BRAVE14 or "Extremism Monitoring Instrument" or "EMI 20" or EMI20 or "Extremism Risk Guidance" or "Extremism Risk Guidelines" or "ERG 22+" or ERG22+ or "IAT 8" or IAT8 or "Identifying Vulnerable People Guidance" or "Intratextual Fundamentalism Scale" or "IR 46" or IR46 or "Militant Extremist Mindset" or "Multi level Guidelines" or "Multi-Dimensional Fundamentalism Inventory" or "Radicalisation Risk Assessment in Prisons" or "Référentiel des indicateurs de basculement dans la radicalisation" or "Religious Fundamentalism Scale" or "Significance Quest Assessment Tool" or "Significance Quest Assessment Test" or SyFoR or "Terrorist Radicalization Assessment Protocol" or "TRAP 18" or TRAP18 or "Violence Threat Risk Assessment" or "Violent Extremism Beliefs Scale" or "Violent Extremism Risk Assessment" or "VERA 2" or VERA2* or "Vulnerability Assessment Framework") OR SU ("Activism and Radicalism Intention Scales" or "Building Resilience to Violent Extremism" or "BRAVE 14" or BRAVE14 or "Extremism Monitoring Instrument" or "EMI 20" or EMI20 or "Extremism Risk Guidance" or "Extremism Risk Guidelines" or "ERG 22+" or ERG22+ or "IAT 8" or IAT8 or "Identifying Vulnerable People Guidance" or "Intratextual Fundamentalism Scale" or "IR 46" or IR46 or "Militant Extremist Mindset" or "Multi level Guidelines" or "Multi-Dimensional Fundamentalism Inventory" or "Radicalisation Risk Assessment in Prisons" or "Référentiel des indicateurs de basculement dans la radicalisation" or "Religious Fundamentalism Scale" or "Significance Quest Assessment Tool" or "Significance Quest Assessment Test" or SyFoR or "Terrorist Radicalization Assessment Protocol" or "TRAP 18" or TRAP18 or "Violence Threat Risk Assessment" or "Violent Extremism Beliefs Scale" or "Violent Extremism Risk Assessment" or "VERA 2" or VERA2* or "Vulnerability Assessment Framework") OR SU ("Activism and Radicalism Intention Scales" or "Building Resilience to Violent Extremism" or "BRAVE 14" or BRAVE14 or "Extremism Monitoring Instrument" or "EMI 20" or EMI20 or "Extremism Risk Guidance" or "Extremism Risk Guidelines" or "ERG 22+" or ERG22+ or "IAT 8" or IAT8 or "Identifying Vulnerable People Guidance" or "Intratextual Fundamentalism Scale" or "IR 46" or IR46 or "Militant Extremist Mindset" or "Multi level Guidelines" or "Multi-Dimensional Fundamentalism Inventory" or "Radicalisation Risk Assessment in Prisons" or "Référentiel des indicateurs de basculement dans la radicalisation" or "Religious Fundamentalism Scale" or "Significance Quest Assessment Tool" or "Significance Quest Assessment Test" or SyFoR or "Terrorist Radicalization Assessment Protocol" or "TRAP 18" or TRAP18 or "Violence Threat Risk Assessment" or "Violent Extremism Beliefs Scale" or "Violent Extremism Risk Assessment" or "VERA 2" or VERA2* or "Vulnerability Assessment Framework") OR AB ("Activism and Radicalism Intention Scales" or "Building Resilience to Violent Extremism" or "BRAVE 14" or BRAVE14 or "Extremism Monitoring Instrument" or "EMI 20" or EMI20 or "Extremism Risk Guidance" or "Extremism Risk Guidelines" or "ERG 22+" or ERG22+ or "IAT 8" or IAT8 or "Identifying Vulnerable People Guidance" or "Intratextual Fundamentalism Scale" or "IR 46" or IR46 or "Militant Extremist Mindset" or "Multi level Guidelines" or "Multi-Dimensional Fundamentalism Inventory" or "Radicalisation Risk Assessment in Prisons" or "Référentiel des indicateurs de basculement dans la radicalisation" or "Religious Fundamentalism Scale" or "Significance Quest Assessment Tool" or "Significance Quest Assessment Test" or SyFoR or "Terrorist Radicalization Assessment Protocol" or "TRAP 18" or TRAP18 or "Violence Threat Risk

Assessment" or "Violent Extremism Beliefs Scale" or "Violent Extremism Risk Assessment" or "VERA 2" or VERA2* or "Vulnerability Assessment Framework") OR KW ("Activism and Radicalism Intention Scales" or "Building Resilience to Violent Extremism" or "BRAVE 14" or BRAVE14 or "Extremism Monitoring Instrument" or "EMI 20" or EMI20 or "Extremism Risk Guidance" or "Extremism Risk Guidelines" or "ERG 22+" or ERG22+ or "IAT 8" or IAT8 or "Identifying Vulnerable People Guidance" or "Intratextual Fundamentalism Scale" or "IR 46" or IR46 or "Militant Extremist Mindset" or "Multi level Guidelines" or "Multi-Dimensional Fundamentalism Inventory" or "Radicalisation Risk Assessment in Prisons" or "Référentiel des indicateurs de basculement dans la radicalisation" or "Religious Fundamentalism Scale" or "Significance Quest Assessment Tool" or "Significance Quest Assessment Test" or SyFoR or "Terrorist Radicalization Assessment Protocol" or "TRAP 18" or TRAP18 or "Violence Threat Risk Assessment" or "Violent Extremism Beliefs Scale" or "Violent Extremism Risk Assessment" or "VERA 2" or VERA2* or "Vulnerability Assessment Framework")

S5: S3 OR S4

Opérateurs de restriction - Date de publication: -20211231
Filters: Magazines, Trade Publications, Book Reviews

RESULTS: 392 (34 after duplicates)

NCJRS (ProQuest)
Search performed August 9, 2022
NOTE: search updated until end of 2021, new strategy run so no start date set.

((noft((risk* OR vulnerabilit* OR recidiv* OR dangerous* OR threat* OR need*) NEAR/3 (assess* OR predict* OR evaluat* OR screen* OR tool* OR protocol* OR scale* OR instrument* OR calibrat* OR index* OR measur*)) AND noft(radicali* OR extremis* OR fundamentalis* OR terroris* OR "hate crime*" OR "religious violence*" OR "political violence*" OR "ideological violence*" OR "environmental violence*" OR "racist violence*" OR "separatist violence*" OR "far right" OR "right wing" OR "alt right" OR "radical right" OR "extreme right" OR "white supremac*" OR "neo nazi*" OR neonazi* OR "anti semiti*" OR antisemiti* OR "anti-semiti*" OR "left wing" OR "far left" OR "alt left" OR "anti fa" OR antifa* OR anarch* OR "anti capitalis*" OR anticapitalis* OR jihadis* OR islamis* OR salafis* OR "lone wolf*" OR "lone actor*" OR "lone offend*" OR "suicide bomb*" OR "suicide attack*" OR "mass shoot*" OR indoctrinat* OR "foreign fight*" OR martyr*)) NOT stype.exact("Newspapers" OR "Wire Feeds" OR "Trade Journals" OR "Other Sources" OR "Magazines" OR "Blogs, Podcasts, & Websites")) OR noft("Activism and Radicalism Intention Scales" OR "Building Resilience to Violent Extremism" OR "BRAVE 14" OR BRAVE14 OR "Extremism Monitoring Instrument" OR "EMI 20" OR EMI20 OR "Extremism Risk Guidance" OR "Extremism Risk Guidelines" OR "ERG 22+" OR ERG22+ OR "IAT 8" OR IAT8 OR "Identifying Vulnerable People Guidance" OR "Intratextual Fundamentalism Scale" OR "IR 46" OR IR46 OR "Militant Extremist Mindset" OR "Multi level Guidelines" OR "Multi-Dimensional Fundamentalism Inventory" OR "Radicalisation Risk Assessment in Prisons" OR "Référentiel des indicateurs de basculement dans la radicalisation" OR "Religious Fundamentalism Scale" OR "Significance Quest Assessment Tool" OR "Significance Quest Assessment Test" OR

SyFoR OR "Terrorist Radicalization Assessment Protocol" OR "TRAP 18" OR TRAP18 OR "Violence Threat Risk Assessment" OR "Violent Extremism Beliefs Scale" OR "Violent Extremism Risk Assessment" OR "VERA 2" OR VERA2* OR "Vulnerability Assessment Framework")

Limited by:
Date: Before 31 December 2021
Excluded: Newspapers, Magazines, Wire Feeds, Trade Journals, Blogs, Other Sources

RESULTS: 768 (572 after duplicates)

Violent Extremism Risk Assessment Tools Review – German Search (Phase 3)

Overview

Source	Initial results	Results after duplicates
Academic Search Complete	13	11
ERIC	0	0
Education Source	0	0
PsycINFO	0	0
Sociological Abstracts	2	2
ProQuest Central	69	68
ProQuest Dissertations	4	4
Medline (PubMed)	320	256
Web of Science	136	118
KrimDoc	43	43
**TOTAL**	**587**	**502**

Academic Search Complete (EBSCO)
Search performed November 21, 2022

S1 TI (((assess* or bewert* or evaluat* or beurteil* or tool* or protokoll* or instrument*) N3 (risk* or risik* or vulnerabil* or legalbewährung* or rückfall* or gefahr`* or bedrohung*)) ) OR SU ( ((assess* or bewert* or evaluat* or beurteil* or tool* or protokoll* or instrument*) N3 (risk* or risik* or vulnerabil* or legalbewährung* or rückfall* or gefahr`* or bedrohung*))) OR AB (((assess* or bewert* or evaluat* or beurteil* or tool* or protokoll* or instrument*) N3 (risk* or risik* or vulnerabil* or legalbewährung* or rückfall* or gefahr`* or bedrohung*)) ) OR KW ( ((assess* or bewert* or evaluat* or beurteil* or tool* or protokoll* or instrument*) N3 (risk* or risik* or vulnerabil* or legalbewährung* or rückfall* or gefahr`* or bedrohung*)))

S2 TI ((radikal* or extremis* or fundamentalis* or terroris* or "Hassverbrechen*" or "Hasskrimi*" or "politisch motivierte Gewalt*" "religiöse Gewalt*" or "politische Gewalt*" or "ideologische Gewalt*" or "rassistische Gewalt*" or "antisemitische Gewalt" or "separatistische Gewalt*" or "rechtsextrem*" or "extreme Rechte" or "Reichsbürger*" or "neonazi*" or "national*" or "antisemit*" or "demokratiefeindlich*" or "linksextremis*" or "antifa*" or "anarch*" or "antikapitalist*" or "dschihadist*" or "islamist*" or "salafist*" or "jihadist*" or "Einzeltäter*" or "Selbstmordansch*" or "Selbstmordattent*" or "Anschlag*" or "Anschläge" or "ausländische Kämpfer" or "foreign fight*" or "Märtyrer*" or "Online-Radikal*")) OR SU ((radikal* or extremis* or fundamentalis* or terroris* or "Hassverbrechen*" or "Hasskrimi*" or "politisch motivierte Gewalt*" "religiöse Gewalt*" or "politische Gewalt*" or "ideologische Gewalt*" or "rassistische Gewalt*" or "antisemitische Gewalt" or "separatistische Gewalt*" or "rechtsextrem*" or "extreme Rechte" or "Reichsbürger*" or "neonazi*" or "national*" or "antisemit*" or "demokratiefeindlich*" or "linksextremis*" or "antifa*" or "anarch*" or "antikapitalist*" or "dschihadist*" or "islamist*" or "salafist*" or "jihadist*" or "Einzeltäter*" or "Selbstmordansch*" or "Selbstmordattent*" or "Anschlag*" or "Anschläge" or "ausländische Kämpfer" or "foreign fight*" or "Märtyrer*" or "Online-Radikal*")) OR AB ((radikal* or extremis* or fundamentalis* or terroris* or "Hassverbrechen*" or "Hasskrimi*" or "politisch

motivierte Gewalt*" "religiöse Gewalt*" or "politische Gewalt*" or "ideologische Gewalt*" or "rassistische Gewalt*" or "antisemitische Gewalt" or "separatistische Gewalt*" or "rechtsextrem*" or "extreme Rechte" or "Reichsbürger*" or "neonazi*" or "national*" or "antisemit*" or "demokratiefeindlich*" or "linksextremis*" or "antifa*" or "anarch*" or "antikapitalist*" or "dschihadist*" or "islamist*" or "salafist*" or "jihadist*" or "Einzeltäter*" or "Selbstmordansch*" or "Selbstmordattent*" or "Anschlag*" or "Anschläge" or "ausländische Kämpfer" or "foreign fight*" or "Märtyrer*" or "Online-Radikal*")) OR KW ((radikal* or extremis* or fundamentalis* or terroris* or "Hassverbrechen*" or "Hasskrimi*" or "politisch motivierte Gewalt*" "religiöse Gewalt*" or "politische Gewalt*" or "ideologische Gewalt*" or "rassistische Gewalt*" or "antisemitische Gewalt" or "separatistische Gewalt*" or "rechtsextrem*" or "extreme Rechte" or "Reichsbürger*" or "neonazi*" or "national*" or "antisemit*" or "demokratiefeindlich*" or "linksextremis*" or "antifa*" or "anarch*" or "antikapitalist*" or "dschihadist*" or "islamist*" or "salafist*" or "jihadist*" or "Einzeltäter*" or "Selbstmordansch*" or "Selbstmordattent*" or "Anschlag*" or "Anschläge" or "ausländische Kämpfer" or "foreign fight*" or "Märtyrer*" or "Online-Radikal*"))

S3 S1 AND S2

S4 "Octagon Intervention" or "Radicalisation Profiling" or "Ra-Prof" or "Screener Islamismus" or "DyRIAS Screener Islamismus" or "radar-ite"

S5 S3 OR S4

Limiters - Language: German
Filtered: Magazines (2)

Results: 13 (11 after duplicates)

Education Source (EBSCO)
Search performed November 21, 2022

S1 TI (((assess* or bewert* or evaluat* or beurteil* or tool* or protokoll* or instrument*) N3 (risk* or risik* or vulnerabil* or legalbewährung* or rückfall* or gefahr`* or bedrohung*)) ) OR SU ( ((assess* or bewert* or evaluat* or beurteil* or tool* or protokoll* or instrument*) N3 (risk* or risik* or vulnerabil* or legalbewährung* or rückfall* or gefahr`* or bedrohung*))) OR AB (((assess* or bewert* or evaluat* or beurteil* or tool* or protokoll* or instrument*) N3 (risk* or risik* or vulnerabil* or legalbewährung* or rückfall* or gefahr`* or bedrohung*)) ) OR KW ( ((assess* or bewert* or evaluat* or beurteil* or tool* or protokoll* or instrument*) N3 (risk* or risik* or vulnerabil* or legalbewährung* or rückfall* or gefahr`* or bedrohung*)))

S2 TI ((radikal* or extremis* or fundamentalis* or terroris* or "Hassverbrechen*" or "Hasskrimi*" or "politisch motivierte Gewalt*" "religiöse Gewalt*" or "politische Gewalt*" or "ideologische Gewalt*" or "rassistische Gewalt*" or "antisemitische Gewalt" or "separatistische Gewalt*" or "rechtsextrem*" or "extreme Rechte" or "Reichsbürger*" or "neonazi*" or "national*" or "antisemit*" or "demokratiefeindlich*" or "linksextremis*" or "antifa*" or "anarch*" or "antikapitalist*" or "dschihadist*" or "islamist*" or "salafist*" or "jihadist*" or

"Einzeltäter*" or "Selbstmordansch*" or "Selbstmordattent*" or "Anschlag*" or "Anschläge" or "ausländische Kämpfer" or "foreign fight*" or "Märtyrer*" or "Online-Radikal*")) OR SU ((radikal* or extremis* or fundamentalis* or terroris* or "Hassverbrechen*" or "Hasskrimi*" or "politisch motivierte Gewalt*" "religiöse Gewalt*" or "politische Gewalt*" or "ideologische Gewalt*" or "rassistische Gewalt*" or "antisemitische Gewalt" or "separatistische Gewalt*" or "rechtsextrem*" or "extreme Rechte" or "Reichsbürger*" or "neonazi*" or "national*" or "antisemit*" or "demokratiefeindlich*" or "linksextremis*" or "antifa*" or "anarch*" or "antikapitalist*" or "dschihadist*" or "islamist*" or "salafist*" or "jihadist*" or "Einzeltäter*" or "Selbstmordansch*" or "Selbstmordattent*" or "Anschlag*" or "Anschläge" or "ausländische Kämpfer" or "foreign fight*" or "Märtyrer*" or "Online-Radikal*")) OR AB ((radikal* or extremis* or fundamentalis* or terroris* or "Hassverbrechen*" or "Hasskrimi*" or "politisch motivierte Gewalt*" "religiöse Gewalt*" or "politische Gewalt*" or "ideologische Gewalt*" or "rassistische Gewalt*" or "antisemitische Gewalt" or "separatistische Gewalt*" or "rechtsextrem*" or "extreme Rechte" or "Reichsbürger*" or "neonazi*" or "national*" or "antisemit*" or "demokratiefeindlich*" or "linksextremis*" or "antifa*" or "anarch*" or "antikapitalist*" or "dschihadist*" or "islamist*" or "salafist*" or "jihadist*" or "Einzeltäter*" or "Selbstmordansch*" or "Selbstmordattent*" or "Anschlag*" or "Anschläge" or "ausländische Kämpfer" or "foreign fight*" or "Märtyrer*" or "Online-Radikal*")) OR KW ((radikal* or extremis* or fundamentalis* or terroris* or "Hassverbrechen*" or "Hasskrimi*" or "politisch motivierte Gewalt*" "religiöse Gewalt*" or "politische Gewalt*" or "ideologische Gewalt*" or "rassistische Gewalt*" or "antisemitische Gewalt" or "separatistische Gewalt*" or "rechtsextrem*" or "extreme Rechte" or "Reichsbürger*" or "neonazi*" or "national*" or "antisemit*" or "demokratiefeindlich*" or "linksextremis*" or "antifa*" or "anarch*" or "antikapitalist*" or "dschihadist*" or "islamist*" or "salafist*" or "jihadist*" or "Einzeltäter*" or "Selbstmordansch*" or "Selbstmordattent*" or "Anschlag*" or "Anschläge" or "ausländische Kämpfer" or "foreign fight*" or "Märtyrer*" or "Online-Radikal*"))

S3 S1 AND S2

S4 "Octagon Intervention" or "Radicalisation Profiling" or "Ra-Prof" or "Screener Islamismus" or "DyRIAS Screener Islamismus" or "radar-ite"

S5 S3 OR S4

RESULTS: 0

ERIC (EBSCO)
Search performed November 21, 2022

S1 TI (((assess* or bewert* or evaluat* or beurteil* or tool* or protokoll* or instrument*) N3 (risk* or risik* or vulnerabil* or legalbewährung* or rückfall* or gefahr`* or bedrohung*)) ) OR SU ( ((assess* or bewert* or evaluat* or beurteil* or tool* or protokoll* or instrument*) N3 (risk* or risik* or vulnerabil* or legalbewährung* or rückfall* or gefahr`* or bedrohung*))) OR AB (((assess* or bewert* or evaluat* or beurteil* or tool* or protokoll* or instrument*) N3 (risk* or risik* or vulnerabil* or legalbewährung* or rückfall* or gefahr`* or bedrohung*))) OR

KW (((assess* or bewert* or evaluat* or beurteil* or tool* or protokoll* or instrument*) N3 (risk* or risik* or vulnerabil* or legalbewährung* or rückfall* or gefahr`* or bedrohung*)))

S2 TI ((radikal* or extremis* or fundamentalis* or terroris* or "Hassverbrechen*" or "Hasskrimi*" or "politisch motivierte Gewalt*" "religiöse Gewalt*" or "politische Gewalt*" or "ideologische Gewalt*" or "rassistische Gewalt*" or "antisemitische Gewalt" or "separatistische Gewalt*" or "rechtsextrem*" or "extreme Rechte" or "Reichsbürger*" or "neonazi*" or "national*" or "antisemit*" or "demokratiefeindlich*" or "linksextremis*" or "antifa*" or "anarch*" or "antikapitalist*" or "dschihadist*" or "islamist*" or "salafist*" or "jihadist*" or "Einzeltäter*" or "Selbstmordansch*" or "Selbstmordattent*" or "Anschlag*" or "Anschläge" or "ausländische Kämpfer" or "foreign fight*" or "Märtyrer*" or "Online-Radikal*")) OR SU ((radikal* or extremis* or fundamentalis* or terroris* or "Hassverbrechen*" or "Hasskrimi*" or "politisch motivierte Gewalt*" "religiöse Gewalt*" or "politische Gewalt*" or "ideologische Gewalt*" or "rassistische Gewalt*" or "antisemitische Gewalt" or "separatistische Gewalt*" or "rechtsextrem*" or "extreme Rechte" or "Reichsbürger*" or "neonazi*" or "national*" or "antisemit*" or "demokratiefeindlich*" or "linksextremis*" or "antifa*" or "anarch*" or "antikapitalist*" or "dschihadist*" or "islamist*" or "salafist*" or "jihadist*" or "Einzeltäter*" or "Selbstmordansch*" or "Selbstmordattent*" or "Anschlag*" or "Anschläge" or "ausländische Kämpfer" or "foreign fight*" or "Märtyrer*" or "Online-Radikal*")) OR AB ((radikal* or extremis* or fundamentalis* or terroris* or "Hassverbrechen*" or "Hasskrimi*" or "politisch motivierte Gewalt*" "religiöse Gewalt*" or "politische Gewalt*" or "ideologische Gewalt*" or "rassistische Gewalt*" or "antisemitische Gewalt" or "separatistische Gewalt*" or "rechtsextrem*" or "extreme Rechte" or "Reichsbürger*" or "neonazi*" or "national*" or "antisemit*" or "demokratiefeindlich*" or "linksextremis*" or "antifa*" or "anarch*" or "antikapitalist*" or "dschihadist*" or "islamist*" or "salafist*" or "jihadist*" or "Einzeltäter*" or "Selbstmordansch*" or "Selbstmordattent*" or "Anschlag*" or "Anschläge" or "ausländische Kämpfer" or "foreign fight*" or "Märtyrer*" or "Online-Radikal*")) OR KW ((radikal* or extremis* or fundamentalis* or terroris* or "Hassverbrechen*" or "Hasskrimi*" or "politisch motivierte Gewalt*" "religiöse Gewalt*" or "politische Gewalt*" or "ideologische Gewalt*" or "rassistische Gewalt*" or "antisemitische Gewalt" or "separatistische Gewalt*" or "rechtsextrem*" or "extreme Rechte" or "Reichsbürger*" or "neonazi*" or "national*" or "antisemit*" or "demokratiefeindlich*" or "linksextremis*" or "antifa*" or "anarch*" or "antikapitalist*" or "dschihadist*" or "islamist*" or "salafist*" or "jihadist*" or "Einzeltäter*" or "Selbstmordansch*" or "Selbstmordattent*" or "Anschlag*" or "Anschläge" or "ausländische Kämpfer" or "foreign fight*" or "Märtyrer*" or "Online-Radikal*"))

S3 S1 AND S2

S4 "Octagon Intervention" or "Radicalisation Profiling" or "Ra-Prof" or "Screener Islamismus" or "DyRIAS Screener Islamismus" or "radar-ite"

S5 S3 OR S4

RESULTS: 0

Sociological Abstracts (ProQuest)
Search performed November 21, 2022

(noft((assess* OR bewert* OR evaluat* OR beurteil* OR tool* OR protokoll* OR instrument*) NEAR/3 (risk* OR risik* OR vulnerabil* OR legalbewährung* OR rückfall* OR gefahr* OR bedrohung*)) AND noft(radikal* OR extremis* OR fundamentalis* OR terroris* OR "Hassverbrechen*" OR "Hasskrimi*" OR "politisch motivierte Gewalt*" "religiöse Gewalt*" OR "politische Gewalt*" OR "ideologische Gewalt*" OR "rassistische Gewalt*" OR "antisemitische Gewalt" OR "separatistische Gewalt*" OR "rechtsextrem*" OR "extreme Rechte" OR "Reichsbürger*" OR "neonazi*" OR "national*" OR "antisemit*" OR "demokratiefeindlich*" OR "linksextremis*" OR "antifa*" OR "anarch*" OR "antikapitalist*" OR "dschihadist*" OR "islamist*" OR "salafist*" OR "jihadist*" OR "Einzeltäter*" OR "Selbstmordansch*" OR "Selbstmordattent*" OR "Anschlag*" OR "Anschläge" OR "ausländische Kämpfer" OR "foreign fight*" OR "Märtyrer*" OR "Online-Radikal*")) OR noft("Octagon Intervention" OR "Radicalisation Profiling" OR "Ra-Prof" OR "Screener Islamismus" OR "DyRIAS Screener Islamismus" OR "radar-ite")

Limited by:
Language:German

RESULTS: 2 (2 after duplicates)

ProQuest Dissertations & Theses Global (ProQuest)
Search performed November 21, 2022

(noft((assess* OR bewert* OR evaluat* OR beurteil* OR tool* OR protokoll* OR instrument*) NEAR/3 (risk* OR risik* OR vulnerabil* OR legalbewährung* OR rückfall* OR gefahr* OR bedrohung*)) AND noft(radikal* OR extremis* OR fundamentalis* OR terroris* OR "Hassverbrechen*" OR "Hasskrimi*" OR "politisch motivierte Gewalt*" "religiöse Gewalt*" OR "politische Gewalt*" OR "ideologische Gewalt*" OR "rassistische Gewalt*" OR "antisemitische Gewalt" OR "separatistische Gewalt*" OR "rechtsextrem*" OR "extreme Rechte" OR "Reichsbürger*" OR "neonazi*" OR "national*" OR "antisemit*" OR "demokratiefeindlich*" OR "linksextremis*" OR "antifa*" OR "anarch*" OR "antikapitalist*" OR "dschihadist*" OR "islamist*" OR "salafist*" OR "jihadist*" OR "Einzeltäter*" OR "Selbstmordansch*" OR "Selbstmordattent*" OR "Anschlag*" OR "Anschläge" OR "ausländische Kämpfer" OR "foreign fight*" OR "Märtyrer*" OR "Online-Radikal*")) OR noft("Octagon Intervention" OR "Radicalisation Profiling" OR "Ra-Prof" OR "Screener Islamismus" OR "DyRIAS Screener Islamismus" OR "radar-ite")

Limited by:
Language:German

RESULTS: 4 (4 after duplicates)

ProQuest Central (ProQuest)
Search performed November 21, 2022

(noft((assess* OR bewert* OR evaluat* OR beurteil* OR tool* OR protokoll* OR instrument*) NEAR/3 (risk* OR risik* OR vulnerabil* OR legalbewährung* OR rückfall* OR gefahr* OR bedrohung*)) AND noft(radikal* OR extremis* OR fundamentalis* OR terroris* OR "Hassverbrechen*" OR "Hasskrimi*" OR "politisch motivierte Gewalt*" "religiöse Gewalt*" OR "politische Gewalt*" OR "ideologische Gewalt*" OR "rassistische Gewalt*" OR "antisemitische Gewalt" OR "separatistische Gewalt*" OR "rechtsextrem*" OR "extreme Rechte" OR "Reichsbürger*" OR "neonazi*" OR "national*" OR "antisemit*" OR "demokratiefeindlich*" OR "linksextremis*" OR "antifa*" OR "anarch*" OR "antikapitalist*" OR "dschihadist*" OR "islamist*" OR "salafist*" OR "jihadist*" OR "Einzeltäter*" OR "Selbstmordansch*" OR "Selbstmordattent*" OR "Anschlag*" OR "Anschläge" OR "ausländische Kämpfer" OR "foreign fight*" OR "Märtyrer*" OR "Online-Radikal*")) OR noft("Octagon Intervention" OR "Radicalisation Profiling" OR "Ra-Prof" OR "Screener Islamismus" OR "DyRIAS Screener Islamismus" OR "radar-ite")

Limited by:
Language:German
Filtered: Newspapers (10), Wire Feeds (15), Trade Journals (1)

RESULTS: 69 (68 after duplicates)

PsycINFO (APA PsycNet)
Search performed November 22, 2022

No matches found for your search: Any Field: radikal* *OR* **Any Field**: extremis* *OR* **Any Field**: fundamentalis* *OR* **Any Field**: terroris* *OR* **Any Field**: "Hassverbrechen*" *OR* **Any Field**: "Hasskrimi*" *OR* **Any Field**: "politisch motivierte Gewalt*" "religiöse Gewalt*" *OR* **Any Field**: "politische Gewalt*" *OR* **Any Field**: "ideologische Gewalt*" *OR* **Any Field**: "rassistische Gewalt*" *OR* **Any Field**: "antisemitische Gewalt" *OR* **Any Field**: "separatistische Gewalt*" *OR* **Any Field**: "rechtsextrem*" *OR* **Any Field**: "extreme Rechte" *OR* **Any Field**: "Reichsbürger*" *OR* **Any Field**: "neonazi*" *OR* **Any Field**: "national*" *OR* **Any Field**: "antisemit*" *OR* **Any Field**: "demokratiefeindlich*" *OR* **Any Field**: "linksextremis*" *OR* **Any Field**: "antifa*" *OR* **Any Field**: "anarch*" *OR* **Any Field**: "antikapitalist*" *OR* **Any Field**: "dschihadist*" *OR* **Any Field**: "islamist*" *OR* **Any Field**: "salafist*" *OR* **Any Field**: "jihadist*" *OR* **Any Field**: "Einzeltäter*" *OR* **Any Field**: "Selbstmordansch*" *OR* **Any Field**: "Selbstmordattent*" *OR* **Any Field**: "Anschlag*" *OR* **Any Field**: "Anschläge" *OR* **Any Field**: "ausländische Kämpfer" *OR* **Any Field**: "foreign fight*" *OR* **Any Field**: "Märtyrer*" *OR* **Any Field**: "Online-Radikal*" *AND* **Language**: German *AND* **Any Field**: (assess* or bewert* or evaluat* or beurteil* or tool* or protokoll* or instrument*) NEAR/3 (risk* or risik* or vulnerabil* or legalbewährung* or rückfall* or gefahr* or bedrohung*)

No matches found for your search: Any Field: "Octagon Intervention" *OR* **Any Field**: "Radicalisation Profiling" *OR* **Any Field**: "Ra-Prof" *OR* **Any Field**: "Screener Islamismus" *OR* **Any Field**: "DyRIAS Screener Islamismus" *OR* **Any Field**: "radar-ite"

Web of Science (A&HCI , ESCI , CPCI-SSH , SSCI)
Search performed November 22, 2022

(assess* or bewert* or evaluat* or beurteil* or tool* or protokoll* or instrument*) NEAR/3 (risk* or risik* or vulnerabil* or legalbewährung* or rückfall* or gefahr* or bedrohung*) (Topic) and radikal* or extremis* or fundamentalis* or terroris* or "Hassverbrechen*" or "Hasskrimi*" or "politisch motivierte Gewalt*" "religiöse Gewalt*" or "politische Gewalt*" or "ideologische Gewalt*" or "rassistische Gewalt*" or "antisemitische Gewalt" or "separatistische Gewalt*" or "rechtsextrem*" or "extreme Rechte" or "Reichsbürger*" or "neonazi*" or "national*" or "antisemit*" or "demokratiefeindlich*" or "linksextremis*" or "antifa*" or "anarch*" or "antikapitalist*" or "dschihadist*" or "islamist*" or "salafist*" or "jihadist*" or "Einzeltäter*" or "Selbstmordansch*" or "Selbstmordattent*" or "Anschlag*" or "Anschläge" or "ausländische Kämpfer" or "foreign fight*" or "Märtyrer*" or "Online-Radikal*" (Topic) and German (Language)

OR

"Octagon Intervention" or "Radicalisation Profiling" or "Ra-Prof" or "Screener Islamismus" or "DyRIAS Screener Islamismus" or "radar-ite" (Topic) and German (Language)

RESULTS: 136 (118 after duplicates)

Medline (PubMed)
Search performed November 22, 2022

Search: (((assess*[Title/Abstract] OR bewert*[Title/Abstract] OR evaluat*[Title/Abstract] OR beurteil*[Title/Abstract] OR tool*[Title/Abstract] OR protokoll*[Title/Abstract] OR instrument*[Title/Abstract]) AND (risk*[Title/Abstract] OR risik*[Title/Abstract] OR vulnerabil*[Title/Abstract] OR legalbewährung*[Title/Abstract] OR rückfall*[Title/Abstract] OR gefahr*[Title/Abstract] OR bedrohung*[Title/Abstract])) AND ((radikal*[Title/Abstract] OR extremis*[Title/Abstract] OR fundamentalis*[Title/Abstract] OR terroris*[Title/Abstract] OR "Hassverbrechen*"[Title/Abstract] OR "Hasskrimi*"[Title/Abstract] OR "politisch motivierte Gewalt*" "religiöse Gewalt*"[Title/Abstract] OR "politische Gewalt*"[Title/Abstract] OR "ideologische Gewalt*"[Title/Abstract] OR "rassistische Gewalt*"[Title/Abstract] OR "antisemitische Gewalt"[Title/Abstract] OR "separatistische Gewalt*"[Title/Abstract] OR "rechtsextrem*"[Title/Abstract] OR "extreme Rechte"[Title/Abstract] OR "Reichsbürger*"[Title/Abstract] OR "neonazi*"[Title/Abstract] OR "national*"[Title/Abstract] OR "antisemit*"[Title/Abstract] OR "demokratiefeindlich*"[Title/Abstract] OR "linksextremis*"[Title/Abstract] OR "antifa*"[Title/Abstract] OR "anarch*"[Title/Abstract] OR "antikapitalist*"[Title/Abstract] OR "dschihadist*"[Title/Abstract] OR "islamist*"[Title/Abstract] OR "salafist*"[Title/Abstract] OR "jihadist*"[Title/Abstract] OR "Einzeltäter*"[Title/Abstract] OR "Selbstmordansch*"[Title/Abstract] OR "Selbstmordattent*"[Title/Abstract] OR "Anschlag*"[Title/Abstract] OR "Anschläge"[Title/Abstract] OR "ausländische Kämpfer"[Title/Abstract] OR "foreign fight*"[Title/Abstract] OR "Märtyrer*"[Title/Abstract] OR "Online-Radikal*"[Title/Abstract]))) AND (German[Language]) Filters: Abstract

OR

Search: ("Octagon Intervention"[Title/Abstract] OR "Radicalisation Profiling"[Title/Abstract] OR "Ra-Prof"[Title/Abstract] OR "Screener Islamismus"[Title/Abstract] OR "DyRIAS Screener Islamismus"[Title/Abstract] OR "radar-ite"[Title/Abstract]) AND (German[Language])

RESULTS: 320 (256 after duplicates)

KrimDok
Search performed November 22, 2022

"(All Fields:radikal* or extremis* or fundamentalis* or terroris* or "Hassverbrechen*" or "Hasskrimi*" or "politisch motivierte Gewalt*" "religiöse Gewalt*" or "politische Gewalt*" or "ideologische Gewalt*" or "rassistische Gewalt*" or "antisemitische Gewalt" or "separatistische Gewalt*" or "rechtsextrem*" or "extreme Rechte" or "Reichsbürger*" or "neonazi*" or "national*" or "antisemit*" or "demokratiefeindlich*" or "linksextremis*" or "antifa*" or "anarch*" or "antikapitalist*" or "dschihadist*" or "islamist*" or "salafist*" or "jihadist*" or "Einzeltäter*" or "Selbstmordansch*" or "Selbstmordattent*" or "Anschlag*" or "Anschläge" or "ausländische Kämpfer" or "foreign fight*" or "Märtyrer*" or "Online-Radikal*" OR All Fields:assess* or bewert* or evaluat* or beurteil* or tool* or protokoll* or instrument* OR All Fields:risk* or risik* or vulnerabil* or legalbewährung* or rückfall* or gefahr* or bedrohung*)"

RESULTS: 37 (37 after duplicates)

"(All Fields:"Octagon Intervention" "Radicalisation Profiling" "Ra-Prof" "Screener Islamismus" "DyRIAS Screener Islamismus" "radar-ite")"

RESULTS: 6 (6 after duplicates)

TOTAL RESULTS: 43

Appendix B

Coding Manual and Coding Sheet

**Coding Manual**	
**Overall**	▪ If there is no info on a variable, indicate "N/A" instead of leaving the case empty<br>▪ Grey variables = sub-variables of a category<br>▪ Blue cases = possible answers in a menu<br>▪ "-" = do not write info there<br>▪ Some reliability and validity data will be impossible to report parsimoniously (i.e., factor analysis results). For such cases, report broad details and clearly indicate where to find the rest in the paper.
**Document ID**	The ID assigned by the library science expert to the manuscript.
**Authors**	List the authors in APA format. Here's a useful reference for APA formatting: https://owl.purdue.edu/owl/research_and_citation/apa_style/apa_style_introduction.html
**Year**	Year of publication of the study.
**V.R. tools studied**	List only tools related to violent radicalization and on which there are data in the study.
**Place published**	*(One choice only)*<br><br>▪ Journal article<br>▪ Book chapter<br>▪ Government report<br>▪ Organization report<br>▪ Thesis/dissertation<br>▪ Other (specify)
**Is the paper eligible for the S.R.?**	Based on reading the full text and scoring the quality of study assessment tool, should the paper be included in the systematic review? If not, you can stop the coding process.
If not, why?	Examples:<br>▪ Out of topic<br>▪ No empirical data<br>▪ Dismal methodological quality
**Data source**	*(Multiple choices possible)*<br><br>▪ Meetings with the participant (e.g., interviews)<br>▪ Private institutional records (e.g., mental health institution/prison records)<br>▪ Publicly available data (e.g., journal articles, biographies, public legal data)<br>▪ Other
**Quantitative study design**	Answer the following questions in relation to the quantitative study design:<br>▪ Is the data cross-sectional (only one time of measurement) or longitudinal (multiple points in time)?<br>▪ If the data is longitudinal, is it retrospective (data is collected about the past of individuals) or prospective (people are followed over time)?<br>▪ List other info about study design that you find relevant

**Sample characteristics**	List sample characteristics:<br>▪ Number of participants<br>▪ Male/female (indicate the split)<br>▪ Age (ideally, report the mean and standard deviation)<br>▪ Country where data was collected<br>▪ Ethno-racial group (indicate percentages or report relevant info in text format)<br>▪ Education (indicate percentages or report relevant info in text format)<br>▪ Employment status (indicate percentages or report relevant info in text format)<br>▪ Religious/ideological affiliation (indicate percentages or report relevant info in text format)<br>▪ List any other info about sampling that you find relevant
**Reliability**	Degree to which the measure of a construct is consistent or dependable.
Inter-rater agreement	Measure of consistency between two or more independent raters (observers) of the same construct.<br>*Usual measure*: Cohen's kappa
Internal consistency	Measure of consistency between different items of the same construct. If a multiple-item construct measure is administered to respondents, the extent to which respondents rate those items in a similar manner is a reflection of internal consistency.<br>*Usual measure*: Cronbach's alpha
**Validity**	Extent to which a measure adequately represents the underlying construct that it is supposed to measure.<br><br>A measure can be reliable but not valid if it is measuring something very consistently, but is consistently measuring the wrong construct. Likewise, a measure can be valid but not reliable if it is measuring the right construct, but not doing so in a consistent manner.
Content	Essentially, check the operationalization against the relevant content domain for the construct. This approach assumes that you have a good detailed description of the content domain, something that's not always true.<br>*Usual measures*: looking at it, literature reviews
Convergent	Closeness with which a measure relates to (or converges on) the construct that it is purported to measure.<br>*Usual measure*: correlation
Discriminant	Degree to which a measure does not measure (or discriminates from) other constructs that it is not supposed to measure. Basically the opposite of convergent validity.<br>*Usual measures*: between measures (correlations expected to be low), between groups (t-test, ANOVA)
Concurrent	How well one measure relates to another concrete criterion that is presumed to occur simultaneously. Basically, predictive validity but with two constructs measured at the same time.<br>*Usual measure*: correlation
Predictive	Degree to which a measure successfully predicts a future outcome that it is theoretically expected to predict.<br>*Usual measures*: regressions, survival analyses, longitudinal analyses

Construct	Even though construct validity is large and theoretically encompasses multiple types of validity mentioned above, we will be using it to denote analyses looking at how items organize into dimensions.<br>*Usual measures*: exploratory factor analysis, confirmatory factor analysis, multidimensional scaling
External	Check if a scale works in another language/country/setting.<br>*Usual measure*: all of the above, compared between group/setting
**Other validation methods**	Was there other relevant quantitative data contributing to the validation of risk scales, e.g., user satisfaction data? If yes, list the results briefly.
**Recommenda-tions**	*(Only list recommendations emerging from results of the study. For example, if, in the intro, the authors explain the objectives of the tool, do not list them in recommendations.)*<br><br>▪ Concerning the tool (e.g., on which groups the tool was predictive, for which outcomes, in which context)<br>▪ For practitioners (e.g., who should use the tool, how)<br>▪ For future research (e.g., next steps to further validate the tool)<br>▪ Other relevant recommendations
**Limitations (authors)**	Note that limitations can also be found outside the limitations section. Look for limitations in the whole paper, especially in the methods section. Ensure that the limitations reported are those of the study and not of the overall literature (e.g., limitations of the field reviewed in the intro).
**Coder ID**	Name/ID of the coder.
**Coding date**	Date where the coding sheet was completed.

Coding Sheet (Inverted for Readability)

Document ID	Study 1	Study 2	Study *n*
Title			
Authors			
Year			
V.R. tools studied			
Place published			
If "other", specify:			
Based on full-text reading, is the paper eligible for the S.R.?			
If not, why?			
Data source	-	-	-
Meetings with the participant			
Private institutional records			
Triangulation of publicly available data			
Other			
Quantitative study design	-	-	-
Cross-sectional vs. longitudinal			
If longitudinal, retrospective or prospective?			
Other relevant info about study design			
Sample characteristics	-	-	-
Number of participants			
Male/female			
Age			
Country where data was collected			
Ethno-racial group			
Education			
Employment status			
Religious/ ideological affiliation			
Any other relevant info			
Reliability	-	-	-
Inter-rater agreement			
Internal consistency			
Other			
Validity	-	-	-
Content			
Convergent			
Discriminant			
Concurrent			
Predictive			
Construct			
External			
Other			

	Study 1	Study 2	Study *n*
**Other validation methods**	-	-	-
User satisfaction			
Other			
**Recommen-dations**	-	-	-
Concerning the tool			
For practitioners			
For future research			
Other			
**Limitations mentioned by the authors**			
**Coder ID**			
**Coding date**			

Appendix C1 - Modified COSMIN Risk of Bias Checklist

Violent Extremism Risk Tools Systematic Review

Items	1 point		0 point		N/A
Risk tool presentation					
1) Is a clear description provided of the construct assessed by the tool?	Yes		No		N/A
2) Is a clear description provided of the target population for which the tool was developed?	Yes		No		N/A
3) Is a clear description provided of the tool's context of use?	Yes		No		N/A
Data analysis (general)					
4) Was an appropriate approach used to analyze the data?	A widely recognized or well justified approach was used	Assumable that the approach was appropriate but not clearly described	Not clear what approach was used or doubtful whether the approach was appropriate	Approach not appropriate	N/A
5) Was the sample size appropriate?	≥ 100	50-99	30-49	<30	N/A
6) Were there any important flaws in the design or statistical methods of the study?	No important methodological flaws		Minor methodolo-gical flaws	Important methodolo-gical flaws	
If there were inter-rater reliability analyses					
7) For dichotomous/nominal/ordinal scores: Was kappa calculated?*	Kappa calculated		No kappa calculated		N/A
8) For ordinal scores: Was a weighted kappa calculated?*	Weighted Kappa calculated		Unweighted Kappa calculated or not described		N/A
If there were internal consistency analyses					
9) Was an internal consistency statistic calculated for each unidimensional scale or subscale separately?	Internal consistency calculated for each unidimensional scale or subscale		Unclear whether scale or subscale is unidimen-sional	Internal consistency not calculated for each unidimen-sional scale or subscale	N/A
10) For continuous scores: Was Cronbach's alpha or omega calculated?*	Cronbach's alpha or omega calculated		Only item-total correlations calculated	No Cronbach's alpha/ omega and no item-total correlations calculated	N/A

11) For dichotomous scores: Was Cronbach's alpha or KR-20 calculated?*	Cronbach's alpha or KR-20 calculated		Only item-total correlations calculated	No Cronbach's alpha/KR-20 and no item-total correlations calculated	N/A
If there were face/content validity analyses					
12) Was each item tested in an appropriate number of participants?	≥ 50	≥ 30	< 30 or not clear		N/A
13) Was an appropriate method used to ask participants about the relevance of each item?	Widely recognized or well justified method used	Assumable that the method was appropriate but not clearly described	Not clear if participants were asked whether each item is relevant or doubtful whether the method was appropriate	Method used not appropriate or participants not asked about the relevance of all items	N/A
14) Was an appropriate method used to ask participants about the tool's comprehensiveness?	Widely recognized or well justified method used	Assumable that the method was appropriate but not clearly described	Doubtful whether the method was appropriate	Method used not appropriate	N/A
15) Was an appropriate method used to ask participants about the comprehensivity of the tool's instructions and items?	Widely recognized or well justified method used	Assumable that the method was appropriate but not clearly described	Doubtful whether the method was appropriate or not clear if participants were asked about the comprehen-sivity of the tool's instructions and items	Method used not appropriate or participants were not asked about the comprehen-sivity of the tool's instructions and items	N/A
If there were convergent validity analyses					
16) Is it clear what the comparator instrument(s) measure(s)?	The constructs measured by comparators are clear		The constructs measured by comparators are not clear		N/A
17) Was the statistical method appropriate for the hypotheses to be tested?	Statistical method was appropriate	Assumable that statistical method was appropriate	Statistical method applied not optimal	Statistical method applied not appropriate	N/A
If there were comparisons between groups (except cross-cultural validity)					
18) Was an adequate description provided of important characteristics of the subgroups?	Adequate description of the important characteris-tics of the subgroups	Adequate description of most of the important characteris-tics of the subgroups	Poor or no description of the important characteristics of the subgroups		N/A

19) Was the statistical method appropriate for the hypotheses to be tested?	Statistical method was appropriate	Assumable that statistical method was appropriate	Statistical method applied not optimal	Statistical method applied not appropriate	N/A
If there were concurrent/predictive validity analyses					
20) For continuous scores: Were correlations, or the area under the receiver operating curve calculated?*	Correlations or AUC calculated		Correlations or AUC not calculated		N/A
21) For dichotomous scores: Were sensitivity and specificity determined?*	Sensitivity and specificity calculated		Sensitivity and specificity not calculated		N/A
If there were factor analyses					
22) Type of factor analysis performed	Confirmatory factor analysis	Exploratory factor analysis	No exploratory or confirmatory factor analysis performed		N/A
23) Was the sample size appropriate for factor analysis?	7 participants per item and ≥ 100	5 participants per item and ≥ 100; OR 6 participants per item but < 100	5 participants per item but < 100	Less than 5 participants per item	N/A
If there were cross-cultural (external) validity analyses					
24) Were the samples similar for relevant characteristics except for the group variable?	Evidence provided that samples were similar for relevant characteris-tics except group variable	Stated (but no evidence provided) that samples were similar for relevant characteris-tics except group variable	Unclear whether samples were similar for relevant characteris-tics except group variable	Samples were not similar for relevant characteris-tics except group variable	N/A

**If the statistical analysis employed is not covered here but is still valid, circle the left choice.*

Number of valid items	
Number of points	
**Total score (%)**	

Appendix C2

COSMIN Checklist Results

Items / Study / Tool	Böckler et al. (2017) DS-I	Powis et al. (2019) ERG22+	Powis et al. (2021) ERG22+	Egan et al. (2016) IVP guidance	Hart et al. (2017) – Study 1 MLG-V2	Hart et al. (2017) – Study 2 MLG-V2	Böckler et al. (2020) TRAP-18
**Risk tool presentation**							
1) Is a clear description provided of the construct assessed by the tool?	Yes	Yes	Yes	Yes	Yes	Yes	Yes
2) Is a clear description provided of the target population for which the tool was developed?	Yes	Yes	Yes	Yes	Yes	Yes	Yes
3) Is a clear description provided of the tool's context of use?	Yes	Yes	Yes	Yes	Yes	Yes	Yes
**Data analysis (general)**							
4) Was an appropriate approach used to analyze the data?	No	Yes	Yes	Yes	Yes	Yes	Yes
5) Was the sample size appropriate?	No	Yes	Yes	Yes	No	No	Yes
6) Were the design and statistical methodology of the study free of any significant flaws?	No	Yes	Yes	No	No	No	No
**If there were inter-rater reliability analyses**							
7) For dichotomous/nominal/ordinal scores: Was kappa calculated?	Yes	Yes	N/A	Yes	No	N/A	N/A
8) For ordinal scores: Was a weighted kappa calculated?	Yes	Yes	N/A	N/A	N/A	N/A	N/A
**If there were internal consistency analyses**							
9) Was an internal consistency statistic calculated for each unidimensional scale or subscale separately?	N/A	N/A	Yes	No	N/A	N/A	N/A
10) For continuous scores: Was Cronbach’s alpha or omega calculated?	N/A	N/A	N/A	Yes	N/A	N/A	N/A
11) For dichotomous scores: Was Cronbach’s alpha or KR-20 calculated?	N/A	N/A	Yes	N/A	N/A	N/A	N/A
**If there were face/content validity analyses**							
12) Was each item tested in an appropriate number of participants?	Yes	N/A	N/A	N/A	N/A	No	N/A
13) Was an appropriate method used to ask participants about the relevance of each item?	Yes	N/A	N/A	N/A	N/A	Yes	N/A
14) Was an appropriate method used to ask participants about the tool's comprehensiveness?	Yes	N/A	N/A	N/A	N/A	N/A	N/A

15) Was an appropriate method used to ask participants about the comprehensibility of the tool's instructions and items?	Yes	N/A	N/A	N/A	N/A	N/A	N/A
**If there were convergent validity analyses**							
16) Is it clear what the comparator instrument(s) measure(s)?	N/A	N/A	N/A	N/A	N/A	Yes	N/A
17) Was the statistical method appropriate for the hypotheses to be tested?	N/A	N/A	N/A	N/A	N/A	Yes	N/A
**If there were comparisons between groups (except cross-cultural validity)**							
18) Was an adequate description provided of important characteristics of the subgroups?	N/A	N/A	N/A	Yes	N/A	N/A	N/A
19) Was the statistical method appropriate for the hypotheses to be tested?	N/A	N/A	N/A	Yes	N/A	N/A	N/A
**If there were concurrent/predictive validity analyses**							
20) For continuous scores: Were correlations or the area under the receiver operating curve calculated?	No	N/A	N/A	Yes	Yes	N/A	Yes
21) For dichotomous scores: Were sensitivity and specificity determined?	No	N/A	N/A	Yes	No	N/A	Yes
**If there were factor analyses**							
22) Was an exploratory or confirmatory factor analysis performed?	N/A	N/A	Yes	N/A	N/A	N/A	N/A
23) Was the sample size appropriate for factor analysis?	N/A	N/A	Yes	N/A	N/A	N/A	N/A
**If there were cross-cultural (external) validity analyses**							
24) Were the samples similar for relevant characteristics except for the group variable?	N/A	N/A	N/A	N/A	N/A	N/A	N/A
**Total score (%)**	64%	100%	100%	85%	56%	70%	88%

Appendix C

COSMIN Checklist Results

Items / Study / Tool	Brugh et al. (2023)	Challacombe & Lucas (2019)	Collins & Clark (2021)	Dmitrieva & Meloy (2022)	Erlandsson & Meloy (2018)	García-Andrade et al. (2019)	Goodwill & Meloy (2019)
**Tool**	TRAP-18	TRAP-18	TRAP-18	TRAP-18	TRAP-18	TRAP-18	TRAP-18
**Risk tool presentation**							
1) Is a clear description provided of the construct assessed by the tool?	Yes	Yes	Yes	Yes	Yes	Yes	Yes
2) Is a clear description provided of the target population for which the tool was developed?	Yes	Yes	Yes	Yes	Yes	No	Yes
3) Is a clear description provided of the tool's context of use?	Yes	Yes	Yes	Yes	Yes	Yes	Yes
**Data analysis (general)**							
4) Was an appropriate approach used to analyze the data?	Yes	Yes	Yes	Yes	Yes	Yes	Yes
5) Was the sample size appropriate?	Yes	Yes	No	No	No	No	Yes
6) Were the design and statistical methodology of the study free of any significant flaws?	No	No	No	No	No	No	Yes
**If there were inter-rater reliability analyses**							
7) For dichotomous/nominal/ordinal scores: Was kappa calculated?	Yes	Yes	N/A	N/A	N/A	N/A	N/A
8) For ordinal scores: Was a weighted kappa calculated?	N/A	No	N/A	N/A	N/A	N/A	N/A
**If there were internal consistency analyses**							
9) Was an internal consistency statistic calculated for each unidimensional scale or subscale separately?	N/A	N/A	N/A	N/A	N/A	N/A	N/A
10) For continuous scores: Was Cronbach's alpha or omega calculated?	N/A	N/A	N/A	N/A	N/A	N/A	N/A
11) For dichotomous scores: Was Cronbach's alpha or KR-20 calculated?	N/A	N/A	N/A	N/A	N/A	N/A	N/A
**If there were face/content validity analyses**							
12) Was each item tested in an appropriate number of participants?	N/A	N/A	N/A	N/A	N/A	N/A	N/A
13) Was an appropriate method used to ask participants about the relevance of each item?	N/A	N/A	N/A	N/A	N/A	N/A	N/A
14) Was an appropriate method used to ask participants about the tool's comprehensiveness?	N/A	N/A	N/A	N/A	N/A	N/A	N/A

15) Was an appropriate method used to ask participants about the comprehensibility of the tool's instructions and items?	N/A	N/A	N/A	N/A	N/A	N/A	N/A
**If there were convergent validity analyses**							
16) Is it clear what the comparator instrument(s) measure(s)?	N/A	N/A	N/A	N/A	Yes	N/A	N/A
17) Was the statistical method appropriate for the hypotheses to be tested?	N/A	N/A	N/A	N/A	No	N/A	N/A
**If there were comparisons between groups (except cross-cultural validity)**							
18) Was an adequate description provided of important characteristics of the subgroups?	N/A	Yes	N/A	N/A	N/A	No	Yes
19) Was the statistical method appropriate for the hypotheses to be tested?	N/A	Yes	N/A	N/A	N/A	Yes	Yes
**If there were concurrent/predictive validity analyses**							
20) For continuous scores: Were correlations or the area under the receiver operating curve calculated?	N/A	Yes	N/A	N/A	N/A	Yes	N/A
21) For dichotomous scores: Were sensitivity and specificity determined?	N/A	N/A	N/A	N/A	N/A	Yes	N/A
**If there were factor analyses**							
22) Was an exploratory or confirmatory factor analysis performed?	N/A	N/A	N/A	N/A	N/A	N/A	N/A
23) Was the sample size appropriate for factor analysis?	N/A	N/A	N/A	N/A	N/A	N/A	N/A
**If there were cross-cultural (external) validity analyses**							
24) Were the samples similar for relevant characteristics except for the group variable?	Yes	N/A	N/A	N/A	N/A	N/A	N/A
**Total score (%)**	88%	75%	67%	67%	63%	60%	100%

Appendix C

COSMIN Checklist Results

Study<br>Tool<br>Items	Kupper & Meloy (2021)<br>TRAP-18	Meloy & Gill (2016)<br>TRAP-18	Meloy et al. (2015)<br>TRAP-18	Meloy et al. (2019)<br>TRAP-18	Meloy et al. (2021)<br>TRAP-18	Beardsley & Beech (2013)<br>VERA
**Risk tool presentation**						
1) Is a clear description provided of the construct assessed by the tool?	Yes	Yes	Yes	Yes	Yes	Yes
2) Is a clear description provided of the target population for which the tool was developed?	Yes	Yes	Yes	Yes	Yes	Yes
3) Is a clear description provided of the tool's context of use?	Yes	Yes	Yes	Yes	Yes	Yes
**Data analysis (general)**						
4) Was an appropriate approach used to analyze the data?	Yes	Yes	Yes	Yes	Yes	Yes
5) Was the sample size appropriate?	No	Yes	No	Yes	Yes	No
6) Were the design and statistical methodology of the study free of any significant flaws?	Yes	No	Yes	No	Yes	No
**If there were inter-rater reliability analyses**						
7) For dichotomous/nominal/ordinal scores: Was kappa calculated?	N/A	N/A	Yes	N/A	N/A	Yes
8) For ordinal scores: Was a weighted kappa calculated?	N/A	N/A	N/A	N/A	N/A	N/A
**If there were internal consistency analyses**						
9) Was an internal consistency statistic calculated for each unidimensional scale or subscale separately?	N/A	N/A	N/A	N/A	N/A	N/A
10) For continuous scores: Was Cronbach’s alpha or omega calculated?	N/A	N/A	N/A	N/A	N/A	N/A
11) For dichotomous scores: Was Cronbach’s alpha or KR-20 calculated?	N/A	N/A	N/A	N/A	N/A	N/A
**If there were face/content validity analyses**						
12) Was each item tested in an appropriate number of participants?	N/A	N/A	No	N/A	Yes	N/A
13) Was an appropriate method used to ask participants about the relevance of each item?	N/A	N/A	N/A	N/A	N/A	N/A
14) Was an appropriate method used to ask participants about the tool's comprehensiveness?	N/A	N/A	N/A	N/A	N/A	N/A

15) Was an appropriate method used to ask participants about the comprehensibility of the tool's instructions and items?	N/A	N/A	N/A	N/A	N/A	N/A
**If there were convergent validity analyses**						
16) Is it clear what the comparator instrument(s) measure(s)?	N/A	N/A	N/A	N/A	N/A	N/A
17) Was the statistical method appropriate for the hypotheses to be tested?	N/A	N/A	N/A	N/A	N/A	N/A
**If there were comparisons between groups (except cross-cultural validity)**						
18) Was an adequate description provided of important characteristics of the subgroups?	Yes	No	Yes	Yes	N/A	N/A
19) Was the statistical method appropriate for the hypotheses to be tested?	Yes	Yes	Yes	Yes	N/A	N/A
**If there were concurrent/predictive validity analyses**						
20) For continuous scores: Were correlations or the area under the receiver operating curve calculated?	N/A	No	N/A	N/A	N/A	N/A
21) For dichotomous scores: Were sensitivity and specificity determined?	N/A	N/A	N/A	N/A	N/A	N/A
**If there were factor analyses**						
22) Was an exploratory or confirmatory factor analysis performed?	N/A	N/A	N/A	N/A	N/A	N/A
23) Was the sample size appropriate for factor analysis?	N/A	N/A	N/A	N/A	N/A	N/A
**If there were cross-cultural (external) validity analyses**						
24) Were the samples similar for relevant characteristics except for the group variable?	N/A	N/A	N/A	N/A	N/A	N/A
**Total score (%)**	88%	67%	80%	88%	100%	71%

Appendix D

Excluded Studies and Reasons for Exclusion

#	Authors (year)	Title	Reason for exclusion
1	Aguerri & Fernández Abad (2021)	*La orden de servicios 3/2018: ¿un instrumento para medir el riesgo de radicalismo violento en prisión?* [The Service Order 3/2018: An Instrument to Measure the Risk of Violent Radicalization in Prison?]	Non-empirical/theoretical paper
2	Ahearn et al. (2020)	What Factors Are Truly Associated with Risk for Radicalisation? A Secondary Data Analysis Within a UK Sample	Tool not operationally usable by clinicians for cases involving individuals
3	Al-Farajat & Al-sharah (2018)	The Ability of Maladaptive Schemas in Prediction of Intellectual Extremism	Not about a risk tool
4	Allely & Faccini (2019)	Clinical Profile, Risk, and Critical Factors and the Application of the "Path Toward Intended Violence" Model in the Case of Mass Shooter Dylann Roof	Not about a risk tool
5	Altemeyer & Hunsberger (2004)	A Revised Religious Fundamentalism Scale: The Short and Sweet of It	Tool not operationally usable by clinicians for cases involving individuals
6	Belanger et al. (2019)	Radicalization Leading to Violence: A Test of the 3N Model	Tool not operationally usable by clinicians for cases involving individuals
7	Besta & Błażek (2007)	*Polska Adaptacja Skali Fundamentalizmu Religijnego Autorstwa B. Altemeyera I B. Hunsbergera* [Polish Adaptation of Religious Fundamentalism Scale by B. Altemeyer and B. Hunsberger]	Tool not operationally usable by clinicians for cases involving individuals
8	Bhui et al. (2014)	Is Violent Radicalisation Associated with Poverty, Migration, Poor Self-Reported Health and Common Mental Disorders?	Tool not operationally usable by clinicians for cases involving individuals
9	Bhui et al. (2020)	Assessing Risks of Violent Extremism in Depressive Disorders: Developing and Validating a New Measure of Sympathies for Violent Protest and Terrorism	Tool not operationally usable by clinicians for cases involving individuals
10	Bhui et al. (2020)	Extremism and Common Mental Illness: Cross-Sectional Community Survey of White British and Pakistani Men and Women Living in England	Tool not operationally usable by clinicians for cases involving individuals
11	Böckler et al. (2015)	The Frankfurt Airport Attack: A Case Study on the Radicalization of a Lone-Actor Terrorist	Qualitative study
12	Bootsma & Harbers (2021)	Assessing Potentially Violent Extremists: Experiences from Dutch Investigative Psychologists	Non-empirical/theoretical paper
13	Brayton (2004)	The Measurement of Bias and Risk Assessment in Perpetrators of Bias Motivated Acts of Violence	Risk tool not for violent radicalization
14	Carlucci et al. (2013)	Factor Structure of the Italian Version of the Religious Fundamentalism Scale	Tool not operationally usable by clinicians for cases involving individuals

15	Carlucci et al. (2015)	Religious Fundamentalism and Psychological Well-Being: An Italian Study	Tool not operationally usable by clinicians for cases involving individuals
16	Clemmow (2020)	Risk Factors and Indicators for Engagement in Violent Extremism	Not about a risk tool
17	Clemmow et al. (2020)	Disaggregating Lone-actor Grievance-Fuelled Violence: Comparing Lone-actor Terrorists and Mass Murderers	Not about a risk tool
18	Conley (2019)	An Examination of Risk Assessment Tools Developed for Radicalized Individuals and Their Application in a Correctional Context	Secondary data
19	Cowan & Cole (2022)	The Pathway to Violence and Public Mass Shooters in Mental Health Treatment Before Attacks	Not about a risk tool
20	Cunningham (2018)	Differentiating Delusional Disorder from the Radicalization of Extreme Beliefs: A 17-Factor Model	Non-empirical/theoretical paper
21	da Silva et al. (2019)	Tracking Narrative Change in the Context of Extremism and Terrorism: Adapting the Innovative Moments Coding System	Not about a risk tool
22	Dean & Pettet (2017)	The 3 R's of Risk Assessment for Violent Extremism	Non-empirical/theoretical paper
23	Dehlin & Galliher (2019)	Young Women's Sexist Beliefs and Internalized Misogyny: Links with Psychosocial and Relational Functioning and Political Behavior	Tool not operationally usable by clinicians for cases involving individuals
24	Denovan et al. (2017)	Perception of Risk and Terrorism-related Behavior Change: Dual Influences of Probabilistic Reasoning and Reality Testing	Not about a risk tool
25	Dover et al. (2007)	The Nature and Structure of Muslim Religious Reflection	Tool not operationally usable by clinicians for cases involving individuals
26	Dunbar et al. (2005)	Assessment of Hate Crime Offenders: The Role of Bias Intent in Examining Violence Risk	Risk tool not for violent radicalization
27	Eisenman & Flavahan (2017)	Canaries in the Coal Mine: Interpersonal Violence, Gang Violence, and Violent Extremism Through a Public Health Prevention Lens	Not about a risk tool
28	Fiedler et al. (2019)	Student Crisis Prevention in Schools: The NETWorks Against School Shootings Program (NETWASS) – An Approach Suitable for the Prevention of Violent Extremism?	Not about a risk tool
29	Furnham et al. (2020)	Correlates of the Militant Extremist Mindset	Tool not operationally usable by clinicians for cases involving individuals
30	Gordon et al. (2017)	Potential Measures for the Pre-Detection of Terrorism	Not about a risk tool
31	Gottschalk & Gottschalk (2004)	Authoritarian and Pathological Hatred: A Social Psychological Profile of the Middle Eastern Terrorist	Tool not operationally usable by clinicians for cases involving individuals
32	Grossman et al. (2017)	Understanding Youth Resilience to Violent Extremism: A Standardised Research Measure	Tool not operationally usable by clinicians for cases involving individuals
33	Grossman et al. (2022)	Youth Resilience to Violent Extremism: Development and Validation of the BRAVE Measure	Tool not operationally usable by clinicians for cases involving individuals

34	Guldimann & Meloy (2020)	Assessing the Threat of Lone-actor Terrorism: The Reliability and Validity of the TRAP-18	Secondary data
35	Hammer & Lazar (2019)	Internal Structure and Criterion Relationships for Long and Brief Versions of the Intratextual Fundamentalism Scale (IFS) Among Israeli Jews	Tool not operationally usable by clinicians for cases involving individuals
36	Harder (2018)	Qualitative Case Study Analysis of Domestic Terrorist Data for Use in Community-Based Counternarrative Program Development	Qualitative study
37	Haroun (2003)	Psychiatric Evaluation of Suspected Terrorists	Not about a risk tool
38	Herzog-Evans (2018)	A Comparison of Two Structured Professional Judgment Tools for Violent Extremism and Their Relevance in the French Context	Non-empirical/theoretical paper
39	Ho et al. (2019)	Terrorism and Mental Illness: A Pragmatic Approach for the Clinician	Not about a risk tool
40	Hung (2017)	A Graph-Based, Systems Approach for Detecting Violent Extremist Radicalization Trajectories and Other Latent Behaviors	Tool not operationally usable by clinicians for cases involving individuals
41	Hung et al. (2018)	INSiGHT: A System to Detect Violent Extremist Radicalization Trajectories in Dynamic Graphs	Tool not operationally usable by clinicians for cases involving individuals
42	Hunsberger (1996)	Religious Fundamentalism, Right-wing Authoritarianism, and Hostility Toward Homosexuals in Non-Christian Religious Groups	Tool not operationally usable by clinicians for cases involving individuals
43	Janjua (2021)	What Predicts the Militant Extremist Mindset? An Investigation into the Relationship Between Violent Extremism, and Personality, Moral Disengagement, and Linguistic Markers	Non-empirical/theoretical paper
44	Kenyon (2020)	Exploring the Role of the Internet in the Radicalisation Process and Offending of Individuals Convicted of Extremist Offences	Does not comprise eligible outcome data
45	Kerodal et al. (2016)	Commitment to Extremist Ideology: Using Factor Analysis to Move Beyond Binary Measures of Extremism	Not about a risk tool
46	Klausen et al. (2016)	Toward a Behavioral Model of "Homegrown" Radicalization Trajectories	Not about a risk tool
47	Klausen et al. (2020)	Radicalization Trajectories: An Evidence-based Computational Approach to Dynamic Risk Assessment of "Homegrown" Jihadists	Not about a risk tool
48	Knudsen (2020)	Measuring Radicalisation: Risk Assessment Conceptualisations and Practice in England and Wales	Non-empirical/theoretical paper
49	Lemieux & Regens (2012)	Assessing Terrorist Risks: Developing an Algorithm-Based Model for Law Enforcement	Tool not operationally usable by clinicians for cases involving individuals
50	Liht et al. (2011)	Religious Fundamentalism: An Empirically Derived Construct and Measurement Scale	Tool not operationally usable by clinicians for cases involving individuals
51	Lloyd & Dean (2015)	The Development of Structured Guidelines for Assessing Risk in Extremist Offenders	Non-empirical/theoretical paper
52	Lloyd (2021)	Making Sense of Terrorist Violence and Building Psychological Expertise	Qualitative study
53	Logan & Sellers (2020)	Risk Assessment and Management in Violent Extremism: A Primer for Mental Health Practitioners	Non-empirical/theoretical paper

54	Lone Jr. (2002)	Right-Wing Authoritarianism and Religious Fundamentalism as Related to Universal-Diverse Orientation	Tool not operationally usable by clinicians for cases involving individuals
55	Loza (2010)	The Prevalence of Middle Eastern Extremist Ideologies Among Some Canadian Offenders	Tool not operationally usable by clinicians for cases involving individuals
56	Loza et al. (2011)	The Prevalence of Extreme Middle Eastern Ideologies Around the World	Tool not operationally usable by clinicians for cases involving individuals
57	Manganelli Rattazzi et al. (2007)	A Short Version of the Right-wing Authoritarianism (RWA) Scale	Tool not operationally usable by clinicians for cases involving individuals
58	Meloy & Genzman (2016)	The Clinical Threat Assessment of the Lone-Actor Terrorist	Qualitative study
59	Meloy & Hoffmann (2021)	International Handbook of Threat Assessment (2nd ed.)	Secondary data
60	Meloy (2018)	The Operational Development and Empirical Testing of the Terrorist Radicalization Assessment Protocol (TRAP-18)	Secondary data
61	Meyer (1976)	Multivariate Analyses of Social and Religious Attitudes	Tool not operationally usable by clinicians for cases involving individuals
62	Morris & Nicoletti (2018)	Kinetic Insider Violence and Mass Shootings	Not about a risk tool
63	Moskalenko & McCauley (2009)	Measuring Political Mobilization: The Distinction Between Activism and Radicalism	Tool not operationally usable by clinicians for cases involving individuals
64	Mourad (2018)	Tailoring Violent Extremism Prevention: A Targeted Intervention Method	Non-empirical/theoretical paper
65	Muluk & Sumaktoyo (2010)	Intratextual Fundamentalism and the Desire for Simple Cognitive Structure: The Moderating Effect of the Ability to Achieve Cognitive Structure	Tool not operationally usable by clinicians for cases involving individuals
66	Pathé et al. (2015)	A Model for Managing the Mentally Ill Fixated Person at Major Events	Not about a risk tool
67	Pendley (2018)	The Cloudy Crystal Ball: Detecting and Disrupting Homegrown Violent Extremism	Qualitative study
68	Petrov et al. (2019)	The Assessment of Tolerance of Military Personnel to Extremist Ideology	Tool not operationally usable by clinicians for cases involving individuals
69	Pressman & Flockton (2012)	Calibrating Risk for Violent Political Extremists and Terrorists: The VERA 2 Structured Assessment	Non-empirical/theoretical paper
70	Pressman & Ivan (2019)	Internet Use and Violent Extremism: A Cyber-VERA Risk Assessment Protocol	Qualitative study
71	Pressman (2009)	Risk Assessment Decisions for Violent Political Extremism	Non-empirical/theoretical paper
72	Pressman (2016)	The Complex Dynamic Causality of Violent Extremism: Applications of the VERA-2 Risk Assessment Method to CVE Initiatives	Non-empirical/theoretical paper
73	Richards (2018)	High Risk or Low Risk: Screening for Violent Extremists in DDR Programmes	Non-empirical/theoretical paper
74	Shaffer & Hastings (2007)	Authoritarianism and Religious Identification: Response to Threats on Religious Beliefs	Tool not operationally usable by clinicians for cases involving individuals

75	Shrestha et al. (2020)	Extreme Adopters in Digital Communities	Tool not operationally usable by clinicians for cases involving individuals
76	Stankov (2018)	Psychological Processes Common to Social Conservatism and Terrorism	Tool not operationally usable by clinicians for cases involving individuals
77	Stankov et al. (2010)	Contemporary Militant Extremism: A Linguistic Approach to Scale Development	Tool not operationally usable by clinicians for cases involving individuals
78	Stankov et al. (2018)	Militant Extremist Mindset and the Assessment of Radicalization in the General Population	Tool not operationally usable by clinicians for cases involving individuals
79	Torregrosa & Panizo (2018)	Risktrack: Assessing the Risk of Jihadi Radicalization on Twitter Using Linguistic Factors	Tool not operationally usable by clinicians for cases involving individuals
80	Trip et al. (2019)	Irrational Beliefs and Personality Traits as Psychological Mechanisms Underlying the Adolescents' Extremist Mind-Set	Tool not operationally usable by clinicians for cases involving individuals
81	Trujillo et al. (2016)	Psychometric Properties of the Spanish Version of the Activism and Radicalism Intention Scale	Tool not operationally usable by clinicians for cases involving individuals
82	Unterrainer et al. (2016)	Vulnerable Dark Triad Personality Facets Are Associated with Religious Fundamentalist Tendencies	Tool not operationally usable by clinicians for cases involving individuals
83	Van Brunt et al. (2017)	An Exploration of the Risk, Protective, and Mobilization Factors Related to Violent Extremism in College Populations	Not about a risk tool
84	Warren et al. (2018)	Operationalizing Theory: A Moral-Situational Action Model for Extremist Violence	Not about a risk tool
85	Warren et al. (2020)	Using the Moral-Situational Action Violence Risk Model for Assessing Women Involved in Extremist Violence: An Empirical Study	Not about a risk tool
86	Weems (1999)	Religiosity and Religious Attitudes as They Relate to Mysticism and Sexual Permissiveness	Tool not operationally usable by clinicians for cases involving individuals
87	Williamson & Ahmad (2007)	Survey Research and Islamic Fundamentalism: A Question About Validity	Qualitative study
88	Williamson et al. (2010)	The Intratextual Fundamentalism Scale: Cross-Cultural Application, Validity Evidence, and Relationship with Religious Orientation and the Big 5 Factor Markers	Tool not operationally usable by clinicians for cases involving individuals
89	Williamson et al. (2014)	Religious Fundamentalism and Racial Prejudice: A Comparison of Implicit and Explicit Approaches	Tool not operationally usable by clinicians for cases involving individuals
90	Wong et al. (2018)	Understanding Youth Activism and Radicalism: Chinese Values and Socialization	Tool not operationally usable by clinicians for cases involving individuals
91	Zierhoffer (2014)	Threat Assessment: Do Lone Terrorists Differ from Other Lone Offenders?	Qualitative study
